# Hydroxy-Substituted Azacalix[4]Pyridines: Synthesis, Structure, and Construction of Functional Architectures

**DOI:** 10.3389/fchem.2019.00553

**Published:** 2019-08-16

**Authors:** En-Xuan Zhang, De-Xian Wang, Mei-Xiang Wang

**Affiliations:** ^1^Beijing National Laboratory for Molecular Sciences, CAS Key Laboratory of Molecular Recognition and Function, Institute of Chemistry, Chinese Academy of Sciences, Beijing, China; ^2^School of Chemical Sciences, University of Chinese Academy of Sciences, Beijing, China; ^3^The Key Laboratory of Bioorganic Phosphorus Chemistry and Chemical Biology (Ministry of Education), Department of Chemistry, Tsinghua University, Beijing, China

**Keywords:** heteracalixaromatics, azacalix[4]pyridine, fragment coupling, functionalization, self-assembly

## Abstract

A number of hydroxyl-substituted azacalix[4]pyridines were synthesized using Pd-catalyzed macrocyclic “2+2” and “3+1” coupling methods and the protection–deprotection strategy of hydroxyl group. While the conformation of the these hydroxyl-substituted azacalix[4]pyridines is fluxional in solution, in the solid state, they adopted shape-persistent 1,3-alternate conformations. Besides, X-ray analysis revealed that the existence of hydroxy groups on the *para*-position of pyridine facilitated the formation of solvent-bridged intermolecular hydrogen bonding for mono-hydroxyl-substituted while partial tautomerization for four-hydroxyl-substituted macrocycles, respectively. Taking the hydroxyl-substituted azacalix[4]pyridines as molecular platforms, multi-macrocycle-containing architectures and functional building blocks were constructed. The self-assembly behavior of the resulting building blocks was investigated in crystalline state.

## Introduction

Design of ingenious macrocyclic molecules has been one of the driving forces to promote the major advances of supramolecular chemistry, which has been manifested by examples of crownether, cyclodextrin, calixarene, resorcinarene, cucurbituril, calixpyrrole, pillarenes, etc. (Lehn et al., 1996). Indeed, macrocyclic compounds provide unique models in the study of non-covalent interactions, and they have been serving as building blocks in the construction of high-level supramolecular architectures. Typical examples such as by anchoring derivative groups on the macrocycles, versatile building blocks, have been prepared and widely applied to the fabrication of molecular devices and smart materials (Chen and Liu, 2010; Guo and Liu, 2014; Ma and Tian, 2014; Strutt et al., 2014; Caricato et al., 2015; Le Poul et al., 2015; Parisi et al., 2016; Murray et al., 2017; Pazos et al., 2018; Wang, 2018; Ogoshi et al., 2019).

Heteracalixaromatics, or heteroatom-bridged calix(het)arenes, are a new type of macrocyclic host molecules (König and Fonseca, 2000; Lhoták, 2004; Morohashi et al., 2006; Maes and Dehaen, 2008; Wang, 2008, 2012; Thomas et al., 2012; Ma and Chen, 2014; Chen and Han, 2018). In comparison with the classical calix[n]arenes in which the phenol moieties are linked by methylene units, heteracalixaromatics enjoy much richer molecular diversity and complexity as the different combinations of various heteroatoms and heteroaromatic rings afford almost limitless macrocyclic compounds. Because of the electronic nature of heteroatoms are different from that of carbon and they are able to conjugate differently with their adjacent aromatics, the incorporation of heteroatoms into the bridging positions and aromatic rings endows heteracalixaromatics unique conformational structures and versatile recognition properties. In particular, heteracalixaromatics show unique association property toward ionic species including cations (Gong et al., 2006a; Ma et al., 2009; Zhang et al., 2009; Fang et al., 2012; Wu et al., 2012, 2013), metal clusters (Gao et al., 2011, Gao et al., 2012; Zhang and Zhao, 2018), anions (Wang et al., 2008, 2010; Wang and Wang, 2013; Luo et al., 2018), and neutral molecules (Wang et al., 2004; Gong et al., 2007; Hu and Chen, 2010). Despite the powerful ability as host molecules, surprisingly, the application of heteracalixaromatics as functional building blocks is obviously underexplored. Herein, we report the facile synthesis and structure of a number of hydroxy-substituted azacalix[4]pyridines. These functionalized macrocycles as molecular platform to construct high-level architectures and functional building blocks were also demonstrated.

## Results and Discussion

### Synthesis

**Scheme 1:**
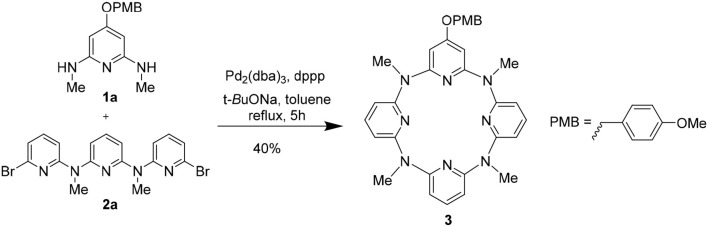
Synthesis of mono-PMB-protected macrocycle **3**.

We attempted to synthesize the hydroxyl-substituted macrocycles from deprotection of the 4-methoxyphenyl (PMB) protected macrocycles. The PMB-protected macrocycle could be obtained from a Pd-catalyzed 3 + 1 coupling method. To access the target macrocycles, the mono-PMB-protected macrocycle **3** was initially examined (Scheme 1). **1a**, which was prepared from nucleophilic substitution reaction between 4-(methoxyphenl)oxy-substituted 2,6-dibromopyridine **1a′** and CH_3_NH_2_ (Figure S1), was applied as the monomeric fragment and reacted with a nitrogen-linked linear trimeric aromatic fragment **2a** (Gong et al., 2006b). The effects of catalyst, ligand and solvent, temperature, and concentration of the substrate were carefully examined (Table S1). It was found that Pd_2_(dba)_3_ (dba = trans,trans-dibenzylideneacetone) showed higher catalytic efficiency than PdCl_2_ and Pd(OAc)_2_ (entries 1–3, Table S1). Dppp [1,3-bis(diphenyphosphino)propane] was shown a better ligand than dppe [1,2-bis(diphenylphosphino)ethane], P(c-Hex)_3_ (tricyclohexylphosphine), and DPEphos [bis(2-dicyclohexylphosphinophenyl)ether] (entries 3–6, Table S1). Among the tested solvents including THF, 1,4-dioxane, o-xylene, and toluene, toluene turned out to be the best to facilitate the macrocyclization (entries 3 and 7–9, Table S1). Reaction temperature is crucial to the cross-coupling reaction. While lower temperature (70°C and 90°C) had a detrimental effect on the reaction, reaction in refluxing toluene could give the macrocyclic product **3** in chemical yield of 32% (entries 3, 10, and 11, Table S1). When 10% mol Pd_2_(dba)_3_, 20% mol dppp, and monomer **1a** at a concentration of 10 mM were employed in refluxing toluene, the macrocyclization gave the highest chemical yield of 40% (entries 12–20, Table S1).

Encouraged by the synthesis of **3**, the synthesis of other PMB-protected macrocycles was then attempted. We envisioned that the 3 + 1 and 2 + 2 coupling strategy could be applicable to obtain these macrocycles. Based on such hypothesis, we prepared different 4-(methoxyphenl)oxy-substituted di-bromopyridine and di-methylaminopyridine fragments, respectively (Figure S1). Pleasantly, both 3 + 1 and 2 + 2 cross-coupling methods worked equally well. Under the optimized conditions for synthesis **3**, the macrocycles **6–9** bearing different numbers (*n* = 2–4) of (4-methoxybenzyl)oxy groups were obtained in acceptable yields (30–34%) (Table 1).

**Table 1:** Synthesis of (4-methoxybenzyl)oxy-protected macrocycles 6–9.

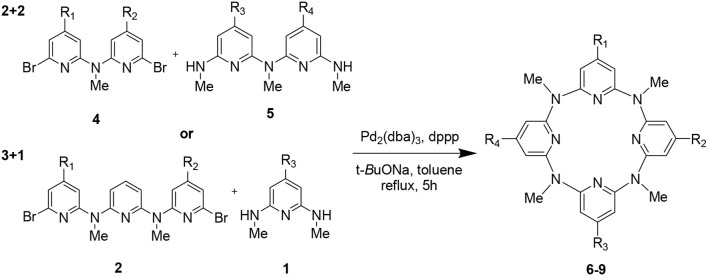			
**Entry**	**Method**	**Product**	**Yield (%)**^**a**^
1	3+1	**6:** R_1_ = R_3_ = OPMB, R_2_ = R_4_ = H	33
2	2+2	**7:** R_1_ = R_2_ = OPMB, R_3_ = R_4_ = H	30
3	3+1	**8:** R_1_ = R_2_ = R_3_ = OPMB, R_4_ = H	34
4	2+2	**9:** R_1_ = R_2_ = R_3_ = R_4_ = OPMB	32

a *Isolated yields*.

**Scheme 2:**
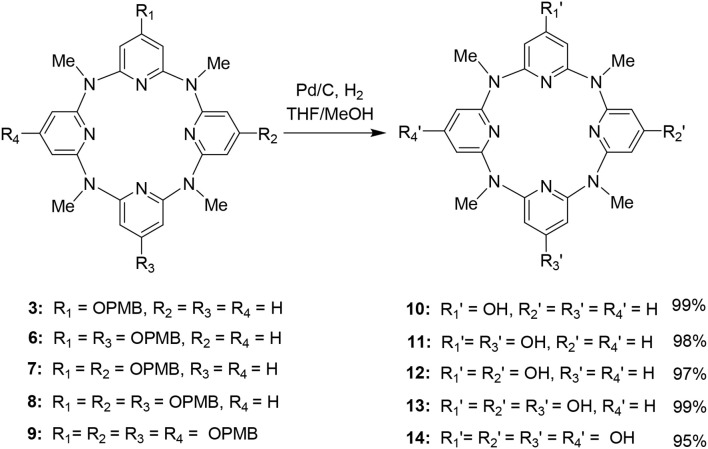
Synthesis of hydroxyl-substituted azacalix[4]pyridines **10–14**.

For the synthesis of hydroxyl-substituted azacalix[4]pyridines **10–14**, the Pb/C-catalyzed hydrogenation reactions were performed on the different protected macrocycles. As shown in Scheme 2, all the reactions proceeded with high efficiency to afford the desired products in the yields ranging from 95 to 99%.

### Structure

**Figure 1:**
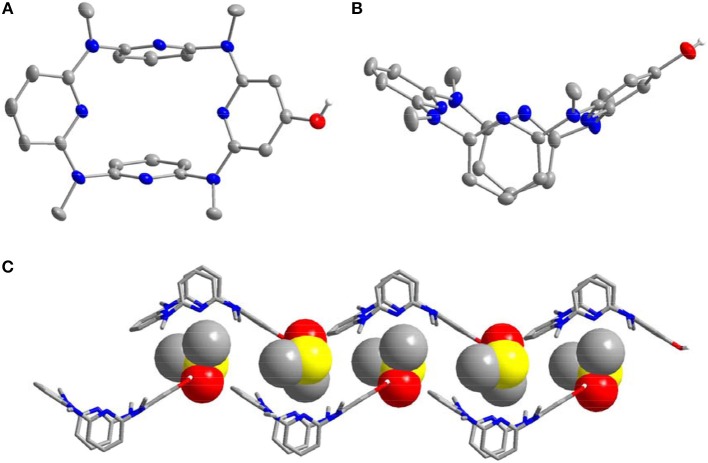
Crystal structure of **10**, top view **(A)** and side view **(B)**, DMSO-separated layer structure through hydrogen bonding **(C)**. Probability is 25%, parts of the hydrogens are omitted for clarity.

**Figure 2:**
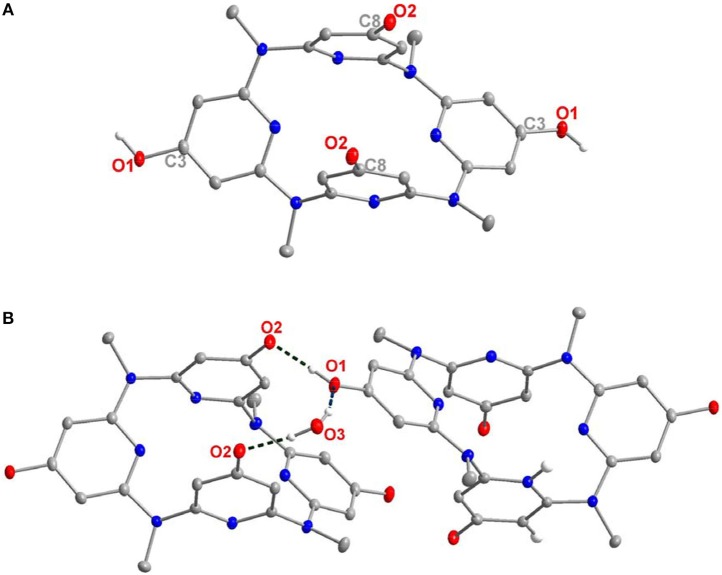
Crystal structure of **14′**, top view **(A)** and dimer structure linked by hydrogen bonding network **(B)**. Selected bond length (Å): C8–O2 1.291, C3–O1 1.349. Selected distance (Å): O2–O1 2.574, O2–O3 2.700, O3–O1 2.887.

The characterization of **10–14** was established on spectroscopic data and elemental analysis. In solution, all the macrocyclic compounds gave one set of ^1^H and ^13^C NMR signals, indicating that they are very fluxional at room temperature and the various conformational structures most probably interconvert rapidly relative to the NMR time scale (Figures S2, S3). Under decreased temperatures (from 298 to 178 K), the conformational interconversion became slow and coexistence of different conformations was clearly observed at 178 K (Figure S4). To probe the structure in solid state, single crystals were cultivated and analyzed by X-ray diffraction method. Pleasantly, slow evaporation of the solutions of **10** (Supplementary Data Sheet 2) and **14** (Supplementary Data Sheet 3) produced single crystals with high quality; the structural details are demonstrated in Figures 1, 2, and Table S2 respectively. In the case of **10**, the molecule shows a similar 1,3-alternate conformation with other azacalix[4]pyridines (Figures 1A,B). While the existence of hydroxyl group on the *para*-position of one pyridine does not affect the conformation of the macrocyclic backbone; it leads to interesting hydrogen-bonded packing. For example, each hydroxyl group as hydrogen bond donor interacts with the oxygen of DMSO; an infinite DMSO-separated layer structure is then produced (Figure 1C).

**Scheme 3:**
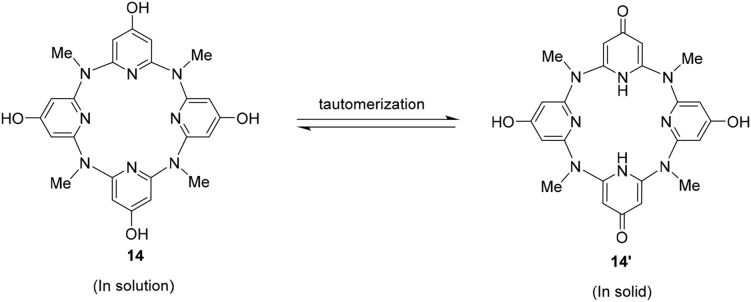
Tautomerization of **14**.

Surprisingly, the structure crystallized from **14** might not be this compound itself. Representative parameters such as two of the C–O distances (d_C8−O2_ = 1.291 Å) is shorter than the other pair (d_C3−O1_ = 1.349 Å). The former distance is typical of C = O double bond, while the latter is C–O single bond as expected (Figure 2). Besides, a dimer structure linked by an O2–O1–O3–O2 hydrogen bonding network could be observed. Here, O2 serves as a hydrogen bond acceptor while the hydroxyl group (O1) or a water molecule (O3) serves as a hydrogen bond donor (Figure 2B). The function of O2 in the hydrogen bonding network is consistent with the nature of carbonyl oxygen. These structural features therefore indicate that the obtained structure is a partially tautomerized compound **14′**, i.e., two of the 4-hydroxyl pyridine of **14** turn to pyridine-4-one moieties. As in solution, **14** gives one set of NMR signals, and the partial tautomerization product is most probably facilitated in solid state (Scheme 3).

### Application of the Hydroxyl-Substituted Azacalix[4]pyridines

**Scheme 4:**
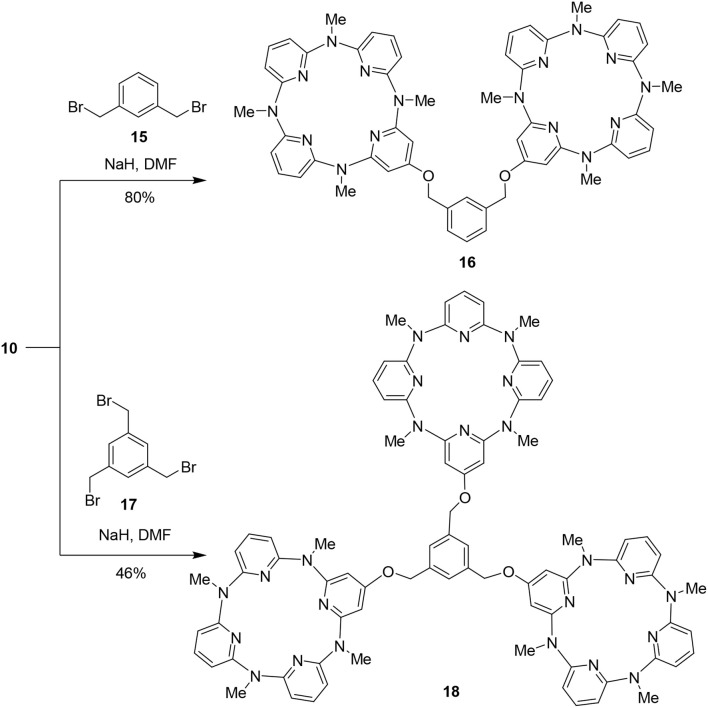
Synthesis of di- and tri-cavity architectures **17** and **18**.

We took the mono- and tetrahydroxy-substituted azacalix[4]pyridines as representative molecular platforms and tested the possibility to construct high-level architectures and functional building blocks. As illustrated in Scheme 4, treatment of **10** with 1,3-bis(bromomethyl)benzene **15** in the presence of NaH in DMF proceeded smoothly to afford a di-cavity compound **16** in 80% yield. When a linker compound 1,3,5-tris(bromomethyl)benzene **17** was applied under the same reaction condition, the tri-cavity compound **18** was obtained in 46% yield (Scheme 4).

**Scheme 5:**
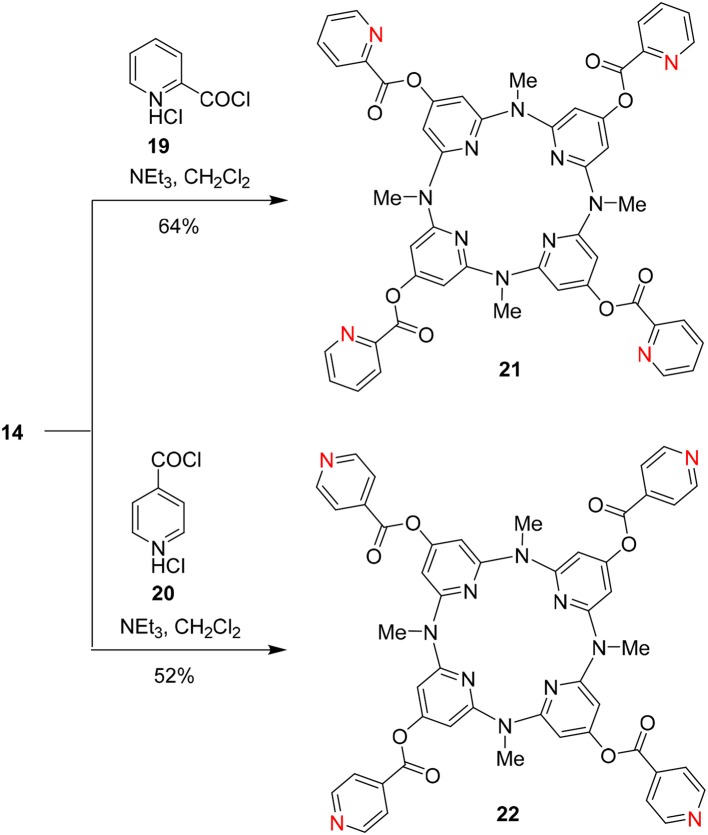
Construction of functional building blocks **21** and **22**.

On the other hand, we applied **14** as the starting materials to react with pyridine-2-acylchloride hydrochloride **19** and pyridine-4-acylchloride hydrochloride **20**, respectively. The reactions in the presence of trimethylammonium in CH_2_Cl_2_ resulted in two pyridine-contained functional building blocks **21** and **22** in 64 and 52% yields, respectively (Scheme 5).

**Figure 3:**
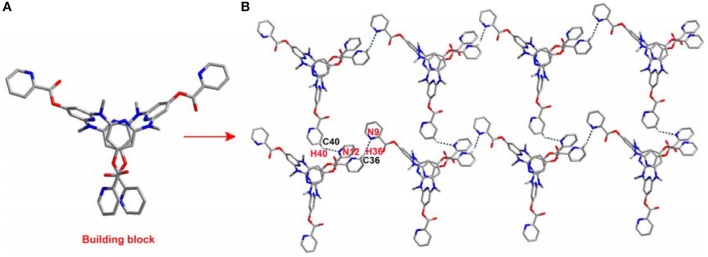
Self-assembly of **21**, **(A)** side view of building block and **(B)** self-assembly structure. Selected hydrogen bonding distance (Å): N9^**…**^H36 2.721, N12^**…**^H40 2.626. Selected hydrogen bonding angle (°): N9–H36–C36 159.6, N12–H40–C40 139.5.

**Figure 4:**
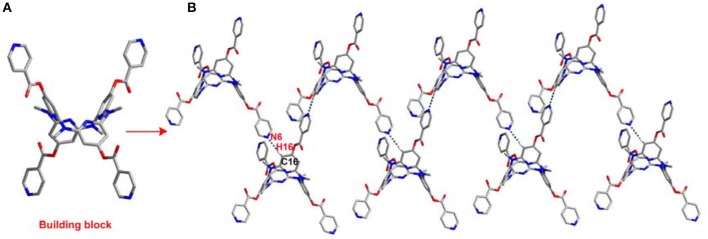
Self-assembly of **22**, **(A)** side view of building block and **(B)** self-assembly structure. Selected hydrogen bonding distance (Å): N6^**…**^H16 2.555. Selected hydrogen bonding angle (°): N6–H16–C16 172.3.

The introduction of the pyridine substituents on azacalix[4]pyridine provides diverse binding sites to facilitate intermolecular self-assembly. To demonstrate the application of the functional building blocks, the self-assembly of **21** and **22** in crystalline state was investigated (Table S3). It is worth addressing that the different pyridine substituents caused significant changes in the conformations. In the case of **21**, the azacalix[4]pyridine backbone maintains the typical 1,3-alternate conformation, i.e., two of the pyridines tend to be edge-to-edge flattened while the other two pyridines tend to be face-to-face paralleled (Figure 3A). For **22**, the molecule exhibits a non-typical orthorhombic 1,3-alternate conformation (Figure 4A). Moreover, due to the different shapes of the building block and different position of nitrogen on the substituent pyridines, the intermolecular hydrogen bonding between pyridine-N and pyridine-H yielded different 2D networks for **21** (Supplementary Data Sheet 4) and **22** (Supplementary Data Sheet 5), respectively. For building block **21**, hydrogen bonding is formed between the substituent pyridines; the interaction of pyridine-N with pyridine-2-H or pyridine-4-H contributes to the formation of hydrogen bond network (Figure 3B), while for **22**, the substituent pyridine-N forms hydrogen bond with the aryl hydrogen of pyridine on the backbone, which produces network with rhombic porosity (Figure 4B).

## Conclusion

In summary, we have synthesized hydroxyl-substituted azacalix[4]pyridines using an efficient protection–deprotection strategy and Pd-catalyzed macrocyclic “2+2” and “3+1” coupling methods. The unique structure and tautomerization of the macrocycle in solid state were revealed by X-ray analysis. This work demonstrated that the synthesized macrocycles could be useful molecular platforms for highly efficient construction of multi-macrocycle-containing architectures and functional building blocks. The high-level architectures and the functional building blocks could find the potential application in fabricating supramolecular or metal-organic porous framework in the future.

## Experimental

### General Information

Chemical shifts are reported in parts per million vs. tetramethylsilane with either tetramethylsilane or the residual solvent resonance used as an internal standard. Melting points are uncorrected. Elemental analyses, mass spectrometry, and X-ray crystallography were performed at the Analytical Laboratory of the Institute. All solvents were dried according to standard procedures prior to use. All other major chemicals were obtained from commercial sources and used without further purification.

#### General Procedure for the Synthesis of (4-methoxybenzyl)Oxy-substituted Macrocycles 3, 6–9

Under argon protection, a mixture of di-methylaminopyridine fragment (2 mmol) and di-bromopyridine fragment (2.2 mmol), Pd_2_(dba)_3_ (184 mg, 0.2 mmol), dppp (164 mg, 0.2 mmol), and sodium tert-butoxide (576 mg, 3 mmol) in anhydrous toluene (400 ml) was heated at reflux for 5 h. The reaction mixture was cooled down to room temperature and filtered through a Celite pad. The filtrate was concentrated under vacuum to remove toluene and the residue was dissolved in dichloromethane (50 ml) and washed with brine (3 × 15 ml). The aqueous phase was re-extracted with dichloromethane (3 × 20 ml), and the combined organic phase was dried over anhydrous Na_2_SO_4_. After removal of solvent, the residue was chromatographed on a silica gel column (100–200) with a mixture of petroleum ether and acetone as the mobile phase to give the products.

#### (4-Methoxybenzyl)Oxy-Substituted Macrocycle 3

A white solid (449 mg, 40% yield); mp 191–193°C; ^1^H NMR (300 MHz, CDCl_3_) δ7.39–7.32 (m, 5H), 6.94 (d, *J* = 8.7 *Hz*, 2H), 6.39 (d, *J* = 8.7 *Hz*, 2H), 6.34–6.30 (m, 4H), 6.04 (s, 2H), 4.97 (s, 2H), 3.82 (s, 3H), 3.20 (s, 6H), 3.17 (s, 6H); ^13^C NMR (75 MHz, CDCl_3_) δ 167.2, 160.2, 159.6, 159.2, 159.0, 138.3, 137.8, 129.2, 128.5, 114.1, 109.5, 107.5, 96.9, 69.4, 55.3, 36.6; IR (KBr) v 1,578, 1,559, 1,515, and 1,473 cm^−1^; MS (MALDI-TOF) *m*/*z* (%) 599 [M+K]^+^ (28), 583 [M+Na]^+^ (50), 561 [M+H]^+^ (100). Anal. Calcd. for C_32_H_32_N_8_O_2_: C, 68.55; H, 5.75; N, 19.99. Found: C, 68.49; H, 5.83; N, 19.91.

#### (4-Methoxybenzyl)Oxy-substituted Macrocycle 6

A white solid (457 mg, 33% yield); mp 273–274°C; ^1^H NMR (300 MHz, CDCl_3_) 7.35 (t, *J* = 7.8 *Hz*, 2H), 7.30 (d, *J* = 8.6 *Hz*, 4H), 6.86 (d, *J* = 8.6 *Hz*, 4H), 6.42 (d, *J* = 7.8 *Hz*, 4H), 5.96 (s, 4H), 4.96 (s, 4H), 3.81 (s, 6H), 3.18 (s, 12H); ^13^C NMR (75 MHz, CDCl_3_) δ 167.7, 160.2, 159.5, 159.1, 138.0, 129.2, 128.7, 114.0, 111.9, 92.4, 69.4, 55.2, 36.6; IR (KBr) v 1,580, 1,559, and 1,515 cm^−1^; MS (MALDI-TOF) *m*/*z* (%) 719 [M+Na]^+^ (40), 697 [M+H]^+^ (100). Anal. Calcd. for C_40_H_40_N_8_O_4_: C, 68.95; H, 5.79; N, 16.08. Found: C, 68.77; H, 5.49; N, 16.28.

#### (4-Methoxybenzyl)Oxy-substituted Macrocycle 7

A white solid (415 mg, 30% yield); mp 98–99°C; ^1^H NMR (300 MHz, CDCl_3_) 7.38 (d, *J* = 8.5 *Hz*, 4H), 7.35 (t, *J* = 7.8 *Hz*, 2H), 6.94 (d, *J* = 8.5 *Hz*, 4H), 6.37 (d, *J* = 7.8 *Hz*, 2H), 6.36 (d, *J* = 7.8 *Hz*, 2H), 6.01 (s, 2H), 6.00 (s, 2H), 4.97 (s, 4H), 3.82 (s, 6H), 3.21 (s, 3H), 3.18 (s, 6H), 3.15 (s, 3H); δ 167.3, 160.1, 159.6, 159.1, 137.9, 129.3, 128.4, 114.1, 108.6, 95.9, 69.4, 55.4, 36.6; IR (KBr) v 1,581, 1,560, 1,514, and 1,468 cm^−1^; MS (MALDI-TOF) *m*/*z* (%) 735 [M+K]^+^ (6), 719 [M+Na]^+^ (14), 697 [M+H]^+^ (100). Anal. Calcd. for C_40_H_40_N_8_O_4_: C, 68.95; H, 5.79; N, 16.08. Found: C, 68.75; H, 5.70; N, 16.21.

#### (4-Methoxybenzyl)Oxy-substituted Macrocycle 8

A white solid (566 mg, 34% yield); mp 101–102°C; ^1^H NMR (300 MHz, CDCl_3_) 7.37 (d, *J* = 8.6 *Hz*, 2H), 7.30 (t, *J* = 7.8 *Hz*, 1H), 7.29 (d, *J* = 8.6 *Hz*, 4H), 6.94 (d, *J* = 8.6 *Hz*, 2H), 6.86 (d, *J* = 8.6 *Hz*, 4H), 6.45 (d, *J* = 7.8 *Hz*, 2H), 6.10 (s, 2H), 5.93 (s, 2H), 5.92 (s, 2H), 4.96 (s, 6H), 3.82 (s, 3H), 3.80 (s, 6H), 3.18 (s, 6H), 3.14 (s, 6H); ^13^C NMR (75 MHz, CDCl_3_) δ 167.7, 166.8, 160.2, 160.1, 159.7, 159.4, 159.1, 137.5, 129.3, 129.2, 128.6, 128.4, 114.2, 114.0, 112.6, 100.0, 91.7, 69.4, 55.3(4), 55.2(8), 36.6; IR (KBr) v 1,583, 1,559, and 1,514 cm^−1^; MS (MALDI-TOF) *m*/*z* (%) 871 [M+K]^+^ (1), 855 [M+Na]^+^ (26), 833 [M+H]^+^ (100). Anal. Calcd. for C_48_H_48_N_8_O_6_: C, 69.21; H, 5.81; N, 13.45. Found: C, 69.19; H, 5.93; N, 13.45.

#### (4-Methoxybenzyl)Oxy-substituted Macrocycle 9

A white solid (620 mg, 32% yield); mp 203–204°C; ^1^H NMR (300 MHz, CDCl_3_) 7.26 (d, *J* = 8.6 *Hz*, 8H), 6.83 (d, *J* = 8.6 *Hz*, 8H), 6.03 (s, 8H), 4.92 (s, 8H), 3.79 (s, 12H), 3.16 (s, 12H); ^13^C NMR (75 MHz, CDCl_3_) δ 167.4, 160.1, 159.4, 129.3, 128.5, 113.9, 95.7, 69.4, 55.2, 36.8; IR (KBr) v 1,583, 1,559, and 1,514 cm^−1^; MS (MALDI-TOF) *m*/*z* (%) 991 [M+Na]^+^ (39), 969 [M+H]^+^ (100). Anal. Calcd. for C_56_H_56_N_8_O_8_: C, 69.41; H, 5.82; N, 11.56. Found: C, 69.29; H, 5.89; N, 11.63.

#### General Procedure for the Synthesis of Hydroxyl-Substituted Azacalix[4]pyridines 10–14

Under nitrogen protection, Pd/C (150 mg, 10 wt%) was added rapidly in a 100-ml round bottom flask with a mixture of PMB-protected macrocycles (2 mmol), THF (20 ml), and methanol (20 ml). The flask was switched three times with hydrogen balloon. The reaction was stopped after reacting at room temperature for 24 h. The reaction mixture was worked up in two ways. Method A: After filtration of the catalyst and removal of the solvent, the residue was chromatographed on a silica gel column (100–200) with a mixture of dichloromethane and methanol as the mobile phase to give the product. Method B: Before filtration of catalyst, the concentrated aqueous ammonia solution was added to the reaction mixture to dissolve the precipitated product. After filtration of the catalyst and removal of the solvent, acetone was added to slurry the residue. The solid was filtered out and washed with a small amount of acetone and dried to give the product.

#### Hydroxyl-Substituted Azacalix[4]pyridine 10

Workup by method A, the product was a white solid (872 mg, 99% yield): 260–262°C; ^1^H NMR (300 MHz, *d*_6_-DMSO) 10.05 (s, 1H), 7.49–7.40 (m, 3H), 6.40–6.34 (m, 6H), 5.82 (s, 2H), 3.10 (s, 6H), 3.03 (s, 6H); ^13^C NMR (75 MHz, *d*_6_-DMSO) δ 166.2, 159.4, 158.4, 158.3, 158.2, 138.6, 138.3, 108.4, 108.2, 96.5, 36.2; IR (KBr) v 3,388, 1,578, and 1,470 cm^−1^; MS (MALDI-TOF) *m*/*z* (%) 479 [M+K]^+^ (5), 463 [M+Na]^+^ (45), 441 [M+H]^+^ (100). Anal. Calcd. for C_24_H_24_N_8_O: C, 65.44; H, 5.49; N, 25.44. Found: C, 65.21; H, 5.54; N, 25.34.

#### Hydroxyl-Substituted Azacalix[4]pyridine 11

Workup by method A, the product was obtained as a white solid (904 mg, 99% yield): > 300°C; ^1^H NMR (300 MHz, *d*_6_-DMSO) 9.93 (s, 2H), 7.39 (t, *J* = 7.7 *Hz*, 2H), 6.48 (d, *J* = 7.7 *Hz*, 4H), 5.70 (s, 4H), 3.03 (s, 12H); ^13^C NMR (75 MHz, *d*_6_-DMSO) δ 166.6, 159.4, 158.5, 138.0, 116.2, 88.6, 36.3; IR (KBr) v 3,368, 3,260, 1,588, and 1,475 cm^−1^; MS (MALDI-TOF) *m*/*z* (%) 495 [M+K]^+^ (5), 479 [M+Na]^+^ (20), 457 [M+H]^+^ (100). exact mass (HRESI) found 457.2092, C_24_H_25_N_8_O_2_ requires: 457.2095.

#### Hydroxyl-Substituted Azacalix[4]pyridine 12

Workup by method A, the product was obtained as a white solid (886 mg, 97% yield): 239–241°C; ^1^H NMR (300 MHz, *d*_6_-DMSO) 10.08 (s, 2H), 7.46 (t, *J* = 7.8 *Hz*, 2H), 6.41 (d, *J* = 7.8 *Hz*, 2H), 6.38 (d, *J* = 7.8 *Hz*, 2H), 5.85 (s, 4H), 3.12 (s, 3H), 3.05 (s, 6H), 2.99 (s, 3H); ^13^C NMR (75 MHz, *d*_6_-DMSO) δ 158.1, 138.3, 108.3, 96.8, 36.3, 36.2; IR (KBr) v 3,401, 1,573, and 1,477 cm^−1^; MS (MALDI-TOF) *m*/*z* (%) 479 [M+Na]^+^ (25), 457 [M+H]^+^ (100). Exact mass (HRESI) found 457.2094, C_24_H_25_N_8_O_2_ requires: 457.2095.

#### Hydroxyl-Substituted Azacalix[4]pyridine 13

Workup by method A, the product was obtained as a white solid (936 mg, 99% yield): > 300°C; ^1^H NMR (300 MHz, *d*_6_-DMSO) 10.08 (s, 1H), 9.90 (s, 2H), 7.41 (t, *J* = 7.8 *Hz*, 1H), 6.49 (d, *J* = 7.8 *Hz*, 2H), 5.92 (s, 2H), 5.67 (s, 2H), 5.64 (s, 2H), 3.06 (s, 6H), 2.96 (s, 6H); ^13^C NMR (75 MHz, *d*_6_-DMSO) δ 166.6, 165.4, 159.5, 159.4, 159.3, 158.4, 137.3, 116.2, 104.5, 88.5, 36.3; IR (KBr) v 3,259, 1,586, and 1,477 cm^−1^; MS (MALDI-TOF) *m*/*z* (%) 495 [M+Na]^+^ (100), 473 [M+H]^+^ (20). Exact mass (HRESI) found 473.2033, C_24_H_25_N_8_O_3_ requires: 473.2044.

#### Hydroxyl-Substituted Azacalix[4]pyridine 14

Workup by method B, the product was obtained as a white solid (928 mg, 95% yield): > 300°C; ^1^H NMR (300 MHz, *d*_6_-DMSO) 9.90 (s, 4H), 5.79 (s, 8H), 2.96 (s, 12H); ^13^C NMR (75 MHz, *d*_6_-DMSO) δ 166.0, 159.4, 95.9, 36.6; IR (KBr) v 3,512, 3,398, 1,578, and 1,490 cm^−1^; MS (MALDI-TOF) *m*/*z* (%) 511 [M+Na]^+^ (74), 489 [M+H]^+^ (100). Exact mass (HRESI) found 489.1983, C_24_H_25_N_8_O_4_ requires: 489.1993.

#### Preparation of Di-Cavity Compound 17

To a solution of **10** (92.5 mg, 0.21 mmol) in dry DMF (2 ml) at room temperature was added NaH (7.2 mg, 0.3 mmol) slowly and the mixture was agitated for 1 h. 1,3-Bis(bromomethyl)benzene **15** (26 mg, 0.1 mmol) was added to the mixture slowly and the reaction mixture was agitated for another 4 h, and then water (20 mL) was added and extracted by ethyl acetate (3 × 20 ml). The organic phase was washed by saturated brine (2 × 20 ml) and dried over anhydrous Na_2_SO_4_. After removal of solvent, the residue was chromatographed on a silica gel column (100–200) with a mixture of dichloromethane and ethyl acetate as the mobile phase to give pure **16** (79 mg, 80%) as a white solid: 253–254°C; ^1^H NMR (300 MHz, CDCl_3_) 7.56 (s, 1H), 7.46–7.45 (m, 3H), 7.35 (t, *J* = 7.8 *Hz*, 6H), 6.38 (d, *J* = 7.8 *Hz*, 4H), 6.35 (d, *J* = 7.8 *Hz*, 4H), 6.33 (d, *J* = 7.8 *Hz*, 4H), 6.04 (s, 4H), 5.10 (s, 4H), 3.20 (s, 12H), 3.17 (s, 12H); ^13^C NMR (75 MHz, CDCl_3_) δ 167.1, 160.2, 159.1, 159.0, 158.9, 138.4, 137.9, 137.0, 129.1, 127.2, 126.4, 108.8, 108.2, 96.1, 69.3, 36.6; IR (KBr) v 1,583 cm^−1^; MS (MALDI-TOF) *m*/*z* (%) 1,005 [M+Na]^+^ (7), 983 [M+H]^+^ (100). Anal. Calcd. for C_56_H_54_N_16_O_2_: C, 68.41; H, 5.54; N, 22.80. Found: C, 68.44; H, 5.55; N, 22.71.

#### Preparation of Tri-Cavity Compound 18

Compound **18** was prepared from **10** and 1,3,5-tribromomesitylene **17** by a similar procedure to the synthesis of **16**. Quantities: **10** (132 mg, 0.3 mmol), NaH (10.8 mg, 0.45 mmol), 1,3,5-tribromomesitylene **17** (35.7 mg, 0.1 mmol), and DMF (2 mL). The product was obtained as a light yellow solid (66 mg, 46%): 208–210°C; ^1^H NMR (300 MHz, CDCl_3_) 7.54 (s, 3H), 7.38–7.33 (m, 9H), 6.39–6.35 (m, 18H), 6.04 (s, 6H), 5.14 (s, 6H), 3.20 (s, 18H), 3.18 (s, 18H); ^13^C NMR (75 MHz, CDCl_3_) δ 167.1, 160.1, 159.1, 159.0, 158.9, 138.4, 138.0, 137.6, 125.9, 108.8(1), 108.7(6), 108.4, 95.6, 69.1, 36.6(4), 36.6(2); IR (KBr) v 1,582 cm^−1^; MS (MALDI-TOF) *m*/*z* (%) 1,473 [M+K]^+^ (2), 1,457 [M+Na]^+^ (9), 1,435 [M+H]^+^ (100). Anal. Calcd. for C_81_H_78_N_24_O_3_:C, 67.77; H, 5.48; N, 23.42. Found: C, 67.60; H, 5.58; N, 23.02.

#### Preparation of Functional Building Block 21

To a solution of **14** (98 mg, 0.2 mmol) in dry dicholormethane (20 ml) at room temperature was added pyridine-2-acylchloride hydrochloride **19** (156 mg, 0.88 mmol) and triethylamine (0.55 ml). After reacting for 24 h, the reaction mixture was washed by saturated Na_2_CO_3_ solution (10 ml) and saturated brine (3 × 20 ml) and then dried over anhydrous Na_2_SO_4_. After removal of solvent, the residue was crystallized by dichloromethane and ethyl acetate to give pure **21** as a light yellow solid (117 mg, 64%): > 300°C; ^1^H NMR (300 MHz, CDCl_3_) 8.68 (d, *J* = 4.6 *Hz*, 4H), 8.13 (d, *J* = 7.8 *Hz*, 4H), 7.76–7.70 (m, 4H), 7.44–7.39 (m, 4H), 6.50 (s, 8H), 3.25 (s, 12H); ^13^C NMR (75 MHz, CDCl_3_) δ 162.5, 160.1, 159.8, 150.0, 147.2, 137.0, 127.2, 125.8, 102.2, 36.8; IR (KBr) v 1,763, 1,744, and 1,581 cm^−1^; MS (ESI) *m*/*z* (%) 931 [M+Na]^+^ (100), 909 [M+H]^+^ (86). Exact mass (HRESI) found 909.2828, C_48_H_37_N_12_O_8_ requires: 909.2852.

#### Preparation of Functional Building Block 22

Compound **22** was prepared from **14** and pyridine-4-acylchloride hydrochloride **20** by a similar procedure to the synthesis of **21**. Quantities: **14** (195 mg, 0.4 mmol), **20** (331 mg, 1.76 mmol), dichloromethane (20 ml), and triethylamine (1.11 ml). The product was obtained as a light yellow solid (190 mg, 52%): 289–291°C; ^1^H NMR (300 MHz, CDCl_3_) 8.74 (d, *J* = 6.0 *Hz*, 8H), 7.84 (d, *J* = 6.0 *Hz*, 8H), 6.46 (s, 8H), 3.26 (s, 12H); ^13^C NMR (75 MHz, CDCl_3_) δ 162.5, 159.8, 159.7, 150.7, 136.4, 123.0, 101.9, 36.8; IR (KBr) v 1,748, 1,607, and 1,571 cm^−1^; MS (ESI) *m*/*z* (%) 931 [M+Na]^+^ (100), 909 [M+H]^+^ (40). Exact mass (HRESI) found 909.2848, C_48_H_37_N_12_O_8_ requires: 909.2852.

## Data Availability

The raw data supporting the conclusions of this manuscript will be made available by the authors, without undue reservation, to any qualified researcher.

## Author Contributions

E-XZ performed the experiments and participated in manuscript preparation. D-XW and M-XW prepared the manuscript.

### Conflict of Interest Statement

The authors declare that the research was conducted in the absence of any commercial or financial relationships that could be construed as a potential conflict of interest.

## References

[B1] Caricato M. Delforge A. Bonifazi D. Dondi D. Mazzanti A. Pasini D. (2015). Chiral nanostructuring multivalent macrocycles in solution and on surfaces. Org. Biomol. Chem. 13, 3593–3601. 10.1039/c4ob02643h 25621466

[B2] Chen C.-F. Han Y. (2018). Triptycene-derived macrocyclic arenes: from calixarenes to helicarenes. Acc. Chem. Res. 51, 2093–2106. 10.1021/acs.accounts.8b00268 30136586

[B3] Chen Y. Liu Y. (2010). Cyclodextrin-based bioactive supramolecular assemblies. Chem. Soc. Rev. 39, 495–505. 10.1039/b816354p 20111774

[B4] Fang Y.-X. Zhao L. Wang D.-X. Wang M.-X. (2012). Synthesis, structure and metal binding property of internally 1,3-arylene-bridged azacalix[6]aromatics. J. Org. Chem. 77, 10073–10082. 10.1021/jo301528f 23101878

[B5] Gao C.-Y. Zhao L. Wang M.-X. (2011). Designed synthesis of metal cluster-centered pseudo-rotaxane supramolecular architectures. J. Am. Chem. Soc. 133, 8448–8451. 10.1021/ja202294v 21561074

[B6] Gao C.-Y. Zhao L. Wang M.-X. (2012). Stabilization of a reactive polynuclear silver carbide cluster through the encapsulation within a supramolecular cage. J. Am. Chem. Soc. 134, 824–827. 10.1021/ja209729h 22185201

[B7] Gong H.-Y. Wang D.-X. Xiang J.-F. Zheng Q.-Y. Wang M.-X. (2007). Highly selective recognition of diols by a self-regulating fine-tunable methylazacalix[4]pyridine cavity: guest-dependent formation of molecular-sandwich and molecular-capsule complexes in solution and the solid state. Chem. Eur. J. 13, 7791–7802. 10.1002/chem.200700498 17583551

[B8] Gong H.-Y. Zhang X.-H. Wang D.-X. Ma H.-W. Zheng Q.-Y. Wang M.-X. (2006a). Methylazacalixpyridines: remarkable bridging nitrogen-tuned conformations and cavities with unique recognition properties. Chem. Eur. J. 212, 9262–9275. 10.1002/chem.200600377 16847994

[B9] Gong H.-Y. Zheng Q.-Y. Zhang X.-H. Wang D.-X. Wang M.-X. (2006b). Methylazacalix[4]pyridine: en route to Zn^2+^-specific fluorescence sensors. Org. Lett. 8, 4895–4898. 10.1021/ol061928k 17020330

[B10] Guo D.-S. Liu Y. (2014). Supramolecular chemistry of *p*-sulfonatocalix[n]arenes and its biological applications. Acc. Chem. Res. 47, 1925–1934. 10.1021/ar500309g 24666259

[B11] Hu S.-Z. Chen C.-F. (2010). Triptycene-derived oxacalixarene with expanded cavity: synthesis, structure and its complexation with fullerenes C_60_ and C_70_. Chem. Commun. 46, 4199–4201. 10.1039/c002944k 20458410

[B12] König B. Fonseca M. H. (2000). Heteroatom-bridged calixarenes. Eur. J. Inorg. Chem. 2000, 2303–2310. 10.1002/1099-0682(200011)2000:11<2303::AID-EJIC2303>3.0.CO;2-Y

[B13] Le Poul N. Le Mest Y. Jabin I. Reinaud O. (2015). Supramolecular modeling of mono-copper enzyme active sites with calix[6]arene-based funnel complexes. Acc. Chem. Res. 48, 2097–2106. 10.1021/acs.accounts.5b00152 26103534

[B14] Lehn J.-M. Atwood J. L. Davies J. D. Macnicol D. D. Vögtle F. (1996). Comprehensive Supramolecular Chemistry. Oxford: Pergamon.

[B15] Lhoták P. (2004). Chemistry of thiacalixarenes. Eur. J. Org. Chem. 2004, 1675–1692. 10.1002/ejoc.200300492

[B16] Luo J. Ao Y.-F. Wang Q.-Q. Wang D.-X. (2018). Diversity-oriented construction and interconversion of multicavity supermacrocycles for cooperative anion-π binding. Angew. Chem. Int. Ed. 57, 15827–15831. 10.1002/anie.201810836 30295403

[B17] Ma M.-L. Li X.-Y. Wen K. (2009). Coordination-driven self-Assembly of a discrete molecular cage and an infinite chain of coordination cages based on *ortho*-linked oxacalix[2]benzene[2]pyrazine and oxacalix[2]arene[2]pyrazine. J. Am. Chem. Soc. 131, 8338–8339. 10.1021/ja900291w 19489554

[B18] Ma X. Tian H. (2014). Stimuli-responsive supramolecular polymers in aqueous solution. Acc. Chem. Res. 47, 1971–1981. 10.1021/ar500033n 24669851

[B19] Ma Y.-X. Chen C.-F. (2014). Triptycene-derived calixarenes. Incl. Phenom. Macrocycl. Chem. 79, 261–281. 10.1007/s10847-013-0372-4

[B20] Maes W. Dehaen W. (2008). Oxacalix[n](het)arenes. Chem. Soc. Rev. 37, 2393–2402. 10.1039/b718356a 18949112

[B21] Morohashi N. Narumi F. Iki N. Hattori T. Miyano S. (2006). Thiacalixarenes. Chem. Rev. 106, 5291–5316. 10.1021/cr050565j 17165689

[B22] Murray J. Kim K. Ogoshi T. Yao W. Gibb B. C. (2017). The aqueous supramolecular chemistry of cucurbit[n]urils, pillar[n]arenes and deep-cavity cavitands. Chem. Soc. Rev. 46, 2479–2496. 10.1039/c7cs00095b 28338130 PMC5462124

[B23] Ogoshi T. Kakuta T. Yamagishi T.-A. (2019). Applications of pillar[n]arene-based supramolecular assemblies. Angew. Chem. Int. Ed. 58, 2197–2206. 10.1002/anie.201805884 29900642

[B24] Parisi M. F. Gattuso G. Notti A. Pisagatti H. Pappalardo S. (2016). Calix[5]arene: From Capsules to Polymers in Calixarenes and Beyond. eds Neri P. Sessler J. L. Wang M.-X. Oxford: Springer.

[B25] Pazos E. Novo P. Peinador C. Kaifer A. E. García M. D. (2018). Cucurbit[8]uril (CB[8])-based supramolecular switches. Angew. Chem. Int. Ed. 57, 2–16. 10.1002/ange.201806575 29978946

[B26] Strutt N. L. Zhang H. Schneebeli S. T. Stoddart J. F. (2014). Functionalizing pillar[n]renes. Acc. Chem. Res. 47, 2630–2642. 10.1021/ar500177d 24999824

[B27] Thomas J. Van Rossom W. Van Hecke K. Van Meervelt L. Smet M. Maes W.. (2012). Selenacalix[3]triazines: synthesis and host–guest chemistry. Chem. Commun. 48, 43–45. 10.1039/c1cc15473g 22005731

[B28] Wang D.-X. Wang M.-X. (2013). Anion–π interactions: generality, binding strength, and structure. J. Am. Chem. Soc. 135, 892–897. 10.1021/ja310834w 23244296

[B29] Wang D.-X. Wang Q.-Q. Han Y. Wang Y. Huang Z.-T. Wang M.-X. (2010). Versatile anion–π interactions between halides and a conformationally rigid bis(tetraoxacalix[2]arene[2]triazine) cage and their directing effect on molecular assembly. Chem. Eur. J. 16, 13053–13057. 10.1002/chem.201002307 20967911

[B30] Wang D.-X. Zheng Q.-Y. Wang Q.-Q. Wang M.-X. (2008). Halide recognition by tetraoxacalix[2]arene[2]triazine receptors: concurrent noncovalent halide-π and lone pair–π interactions in host–halide–water ternary complexes. Angew. Chem. Int. Ed. 47, 7485–7488. 10.1002/anie.200801705 18756571

[B31] Wang M.-X. (2008). Heterocalixaromatics, new generation macrocyclic host molecules in supramolecular chemistry. Chem. Commun. 2008, 4541–4551. 10.1039/b809287g 18815679

[B32] Wang M.-X. (2012). Nitrogen and oxygen bridged calixaromatics: synthesis, structure, functionalization, and molecular recognition. Acc. Chem. Res. 45, 182–195. 10.1021/ar200108c 21834499

[B33] Wang M.-X. (2018). Coronarenes: recent advances and perspectives on macrocyclic and supramolecular chemistry. Sci. China Chem. 61, 993–1003. 10.1007/s11426-018-9328-8

[B34] Wang M.-X. Zhang X.-H. Zheng Q.-Y. (2004). Synthesis, structure, and [60]fullerene complexation properties of azacalix[*m*]arene[*n*]pyridines. Angew. Chem. Int. Ed. 43, 838–842. 10.1002/anie.200351975 14767953

[B35] Wu J.-C. Zhao L. Wang D.-X. Wang M.-X. (2012). Structural diversity in coordination self-assembled networks of a multimodal ligand azacalix[4]pyrazine. Inorg. Chem. 51, 3860–3867. 10.1021/ic3000679 22385357

[B36] Wu J.-C. Zhao L. Wang D.-X. Wang M.-X. (2013). Synthesis, structure and coordination self-assembly of azacalix[4-*n*]pyridine[*n*]pyrazines (n-1-3). Chin. J. Chem. 31, 589–597. 10.1002/cjoc.201300078

[B37] Zhang E.-X. Wang D.-X. Huang Z.-T. Wang M.-X. (2009). Synthesis of (NH)_m_(NMe)_4−m_-bridged calix[4]pyridines and the effect of NH bridge on structure and properties. J. Org. Chem. 74, 8595–8603. 10.1021/jo901609u 19856898

[B38] Zhang S. Zhao L. (2018). Macrocycle-encircled polynuclear metal clusters: controllable synthesis, reactivity studies, and applications. Acc. Chem. Res. 51, 2535–2545. 10.1021/acs.accounts.8b00283 30199219

