# Supplementary material for: Hydroxy-Substituted Azacalix[4]Pyridines: Synthesis, Structure, and Construction of Functional Architectures
Source: Front Chem. 2019 Aug 16;7:553. doi: 10.3389/fchem.2019.00553 (PMC6707087; doi:10.3389/fchem.2019.00553)
Supplement: Supplementary file 1 [file Data_Sheet_1.PDF]

Supplementary Material

Hydroxy-Substituted Azacalix[4]pyridines: Synthesis, Structure and Construction of Functional Architectures

En-Xuan Zhang,[1] De-Xian Wang [1,3,*] Mei-Xiang Wang[2,*]

[1] Beijing National Laboratory for Molecular Sciences, CAS Key Laboratory of Molecular Recognition and Function, Institute of Chemistry, Chinese Academy of Sciences, Beijing, 100190, China

[2] The Key Laboratory of Bioorganic Phosphorus Chemistry & Chemical Biology (Ministry of Education), Department of Chemistry, Tsinghua University, Beijing 100084, China

[c] University of Chinese Academy of Sciences, Beijing 100049, China

dxwang@iccas.ac.cn; wangmx@mail.tsinghua.edu.cn

Contents	Figure, Table	Page
1. Synthesis of (4-methoxybenzyl)oxy-protected dimethylaminopyridine and (4-methoxybenzyl)oxy-protected di-bromopyridine fragments	**Figure S1**	S3
2. Condition optimization for the synthesis of mono-(4-methoxybenzyl)oxyprotected macrocycle **3**	**Table S1**	S4
3. Experimental details and characterizations of products.		S5-S11
4. ^{1}H NMR spectra of hydroxyl-substituted azacalix[4]pyridines **10-14** in d^{6}-DMSO at 298K.	**Figure S2**	S11
5. ^{13}C NMR spectra of hydroxyl-substituted azacalix[4]pyridines **10-14** in d^{6}-DMSO at 298K.	**Figure S3**	S12
6. VT-NMR of **11**	**Figure S4**	S13
7. Copies of ^{1}H and ^{13}C NMR Spectra of products		S14-S55
8. Crystallographic data	**Table S2, S3**	S57

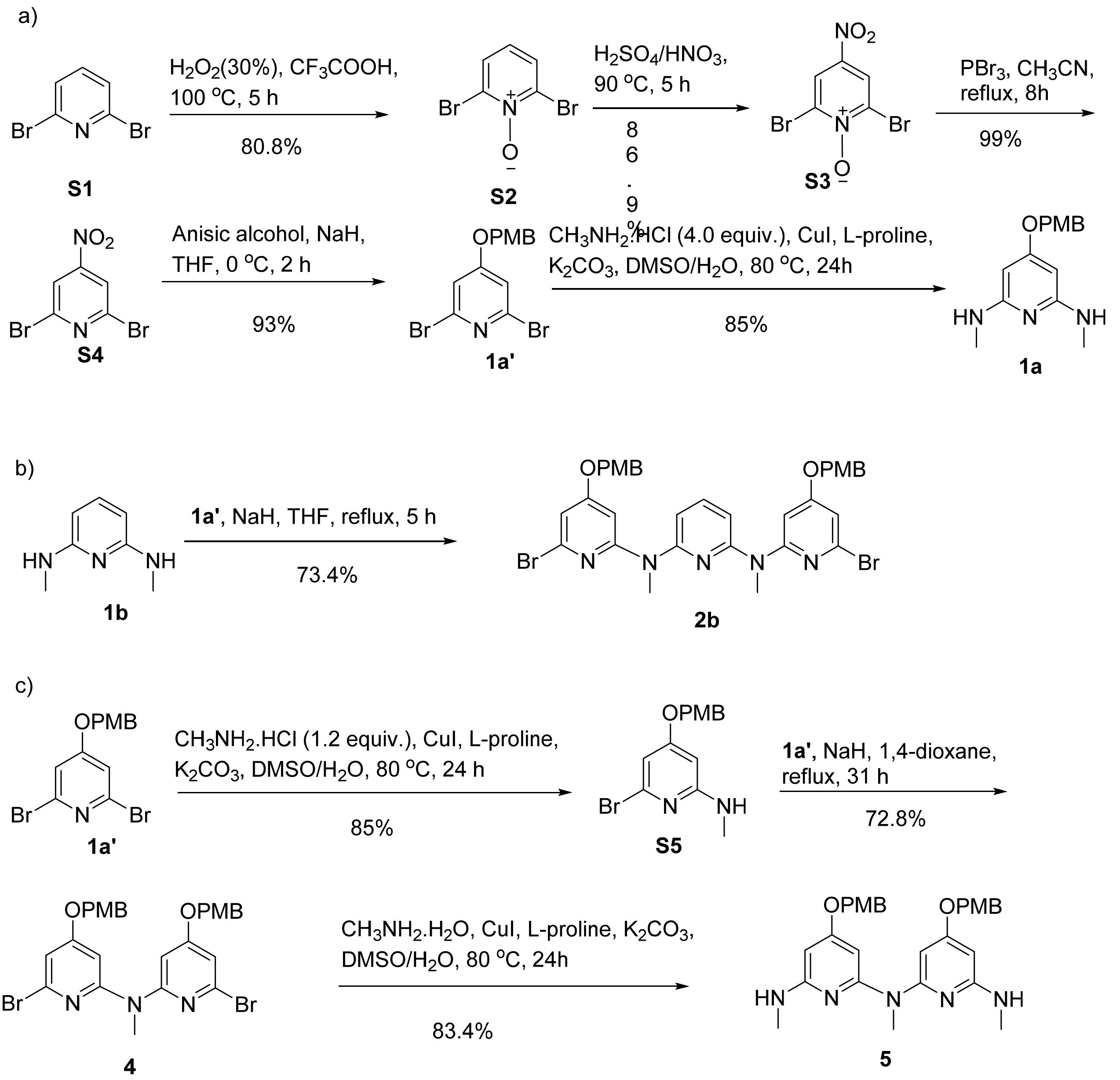

Figure S1. Synthesis of (4-methoxybenzyl)oxy-protected dimethylaminopyridine and (4-methoxybenzyl)oxy-protected di-bromopyridine fragments

Table S1. Condition optimization for the synthesis of mono-(4-methoxybenzyl)oxy-protected macrocycle **3**.[a]

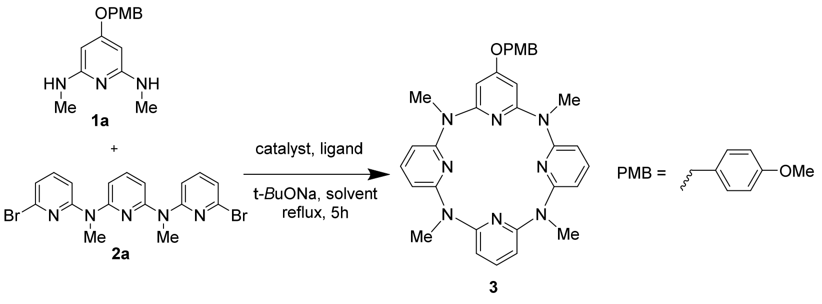

Entry	Catal. (mol %)	Ligand (mol %)	Solvent	Yield (%)[h]
1	$PdCl_2$ (20)	dppp (20)	toluene	18
2	$Pd(OAc)_2$ (20)	dppp (20)	toluene	18
3	$Pd_2(dba)_3$ (10)	dppp (20)	toluene	32
4	$Pd_2(dba)_3$ (10)	dppe (20)	toluene	7
5	$Pd_2(dba)_3$ (10)	P(*c*-Hex)$_3$ (40)	toluene	---
6	$Pd_2(dba)_3$ (10)	DPEphos (20)	toluene	14
7	$Pd_2(dba)_3$ (10)	dppp (20)	THF	---
8	$Pd_2(dba)_3$ (10)	dppp (20)	1,4-dioxane	12
9	$Pd_2(dba)_3$ (10)	dppp (20)	o-xylene	18
10[b]	$Pd_2(dba)_3$ (10)	dppp (20)	toluene	---
11[c]	$Pd_2(dba)_3$ (10)	dppp (20)	toluene	8.6
12[d]	$Pd_2(dba)_3$ (10)	dppp (20)	toluene	32
13[e]	$Pd_2(dba)_3$ (10)	dppp (20)	toluene	38
14[f]	$Pd_2(dba)_3$ (10)	dppp (20)	toluene	40
15[g]	$Pd_2(dba)_3$ (10)	dppp (20)	toluene	29
16	$Pd_2(dba)_3$ (2)	dppp (4)	toluene	19
17	$Pd_2(dba)_3$ (5)	dppp (10)	toluene	32
18	$Pd_2(dba)_3$ (15)	dppp (30)	toluene	31
19	$Pd_2(dba)_3$ (20)	dppp (40)	toluene	21
20	$Pd_2(dba)_3$ (30)	dppp (60)	toluene	15

[a] 3 equiv. *t*-BuONa was used, The ratio of the reactants **1a** and **2a** was 1:1.1, and concentration of Substrate **1a** was 5 × 10^{-3} M. [b] Reaction temperature was 70 °C and reaction time was 10 h. [c] Reaction temperature was 90 °C and reaction time was 10 h. [d-g] Concentration of Substrate **1a** was 2.5, 7.5, 10 and 15 × 10^{-3} M, respectively. [h] Isolated chemical yield. "**---**" indicates that a trace amount or none of the product was isolated.

Experimental details and characterizations of products.

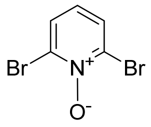

S2

2,6-Dibromopyridine 1-oxide S2: To a solution of 2,6-Dibromopyridine **S1** (14.22 g, 60 mmol) in trifluoroacetic acid (100mL) at room temperature was added 30wt% H_2O_2 (10 mL) slowly and the mixture was heated to 100 °C. After 5 h, the solvent was removed under reduced pressure, and the residue was dissolved in dichloromethane (200 mL). The organic solution was washed with saturated Na_2CO_3 solution (3×50 mL), brine (2×50 mL) and dried over anhydrous Na_2SO_4. After removal of solvent, the residue was chromatographed on a silica gel column with a mixture of petroleum ether and ethyl acetate as the mobile phase to give pure **S2** (12.26g, 81%) as a colorless block: mp 191-193 °C; ^{1}H NMR (300 MHz, $CDCl_3$) δ 7.68 (d *J* = 8.1 Hz, 1H), 6.98 (t, *J* = 8.1Hz, 2H); IR (KBr) ν 3102, 3078, 1439 cm^{-1}.

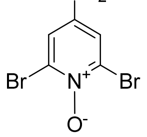

S3

2,6-Dibromo-4-nitropyridine 1-oxide S3: To a solution of 2,6-Dibromopyridine 1-oxide **S2** (22.77 g, 90 mmol) in fuming nitric acid (50 mL) cooled by mixture of ice and water was added concentrated sulfuric acid (150 mL) slowly and the mixture was heated to 90 °C. After 5 h, the mixture was cooled to room temperature, and then was poured into a mixture of 400 mL ice-water. A light

solid was precipitated, filtered, washed with distilled water and dried with infrared light to give pure **S3** (25.29g, 87%) as a light yellow solid: mp 228-229 ºC; ^{1}H NMR (300 MHz, $CDCl_3$) δ 8.49 (s, 1H); IR (KBr) ν 3090, 1519 cm^{-1}.

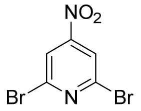

S4

2,6-Dibromo-4-nitropyridine S4: To a solution of 2,6-Dibromo-4-nitropyridine 1-oxide **S3** (11.92 g, 40 mmol) in dry acetonitrile (100 mL) at room temperature was added PBr_3 (21.68 g, 80 mmol) slowly and the mixture was heated to reflux. After refluxing for 8 h, the mixture was cooled to room temperature, and then was poured into a mixture of 400 mL ice-water. A light solid was precipitated, filtered and washed with distilled water. The solid was dissolved by dichloromethane and chromatographed on a basic alumina column with a mixture of petroleum ether and dichloromethane as the mobile phase to give pure **S4** (11.25 g, 99%) as a light yellow block: mp 128-129 ºC; ^{1}H NMR (300 MHz, $CDCl_3$) δ 8.19 (s, 1H); IR (KBr) ν 1541 cm^{-1}.

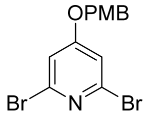

1a'

2,6-dibromo-4-((4-methoxybenzyl)oxy)pyridine 1a': To a solution of *p*-methoxybenzyl alcohol (8.28 g, 60 mmol) in dry THF (50 mL) at room temperature was added NaH (1.44 g, 60 mmol) slowly and the mixture was agitated for 1h and then was cooled to 0 ºC. 2,6-Dibromo-4-nitropyridine **S4** (14 g, 50 mmol) in dry THF (50 mL) was added to the mixture slowly. After another 1 h, the reaction mixture was quenched by saturated NH_4Cl solution (50 mL) slowly and extracted by ethyl acetate (3 × 200 mL). The combined organic solution was dried over anhydrous Na_2SO_4. After

removal of solvent, the residue was chromatographed on a silica gel column with a mixture of petroleum ether and ethyl acetate as the mobile phase to give pure **1a'** (17.38 g, 93%) as a white solid: mp 66-67 °C; ^{1}H NMR (300 MHz, $CDCl_3$) δ 7.30 (dd, *J* = 8.7 Hz, 2H), 7.01 (s, 2H), 6.92 (d, *J* = 8.7 Hz, 2H), 4.98 (s, 2H), 3.80 (s, 3H); ^{13}C NMR (75 MHz, $CDCl_3$) δ 166.8, 160.1, 141.1, 129.6, 126.4, 114.3, 114.1, 70.8, 55.4; IR (KBr) ν 1571, 1535, 1515 cm^{-1}; MS (EI) *m/z* (%): 375 (1), 373 (2), 371 $[M]^+$ (1), 255 (2), 253 (4), 251 (2), 122 (5), 121 (100). Anal. Calcd. for $C_{13}H_{11}Br_2NO_2$: C, 41.86; H, 2.97; N, 3.75. Found: C, 42.01; H, 3.04; N, 3.69.

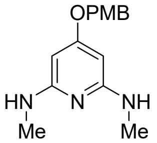

1a

4-((4-Methoxybenzyl)oxy)- 2,6-Bis(methylamino)pyridine 1a: An autoclave equipped with a magnetic stir bar was charged with 2,6-dibromo-4-((4-methoxybenzyl)oxy)pyridine **1a'** (3.73 g, 10 mmol), methylamine hydrochloride (2.7 g, 40 mmol), CuI (380 mg, 2 mmol), L-proline (460 mg, 2 mmol), K_2CO_3 (8.28 g, 60 mmol)， DMSO (30 mL) and water (3 mL). Then it was heated to 80 °C for 24 h, and then cooled to room temperature. The mixture was poured into a mixture of 100 mL water and extracted by ethyl acetate (3 × 200 mL). The combined organic solution was washed with brine (3 × 250mL) and dried over anhydrous Na_2SO_4. After removal of solvent, the residue was chromatographed on a basic alumina column with a mixture of dichloromethane and ethyl acetate as the mobile phase to give pure **1a** (2.32g, 85%) as a white solid: mp 105-106 °C; ^{1}H NMR (300 MHz, $CDCl_3$) δ 7.33 (d, *J* = 8.6 Hz, 2H), 6.90 (d, *J* = 8.6 Hz, 2H), 5.39 (s, 2H), 4.96 (s, 2H), 4.29 (brs, 2H), 3.80 (s, 3H), 2.80 (d, *J* = 5.2 Hz, 6H); ^{13}C NMR (75 MHz, $CDCl_3$) δ 168.4, 160.5, 159.5, 129.3, 128.8, 114.0, 81.3, 69.2, 55.3, 29.2; IR (KBr) ν 3312, 1591, 1518 cm^{-1};

MS (EI) *m/z* (%): 274 (6), 273 $[M]^+$ (41), 258 (7), 124 (10), 121 (100). Anal. Calcd. for $C_{15}H_{19}N_3O_2$: C, 65.91; H, 7.01; N, 15.37. Found: C, 65.92; H, 6.99; N, 15.38.

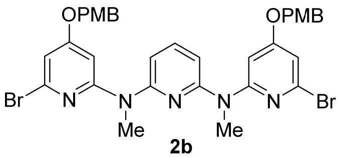

2b

N2,N6-bis(6-bromo-4-((4-methoxybenzyl)oxy)pyridin-2-yl)-N2,N6-dimethylpyridine-2,6-diamine 2b: To a solution of 2,6-Bis(methylamino)pyridine **1b** (274 mg, 2 mmo) in dry THF (50 mL) at room temperature was added NaH (0.96 g, 40 mmol) slowly and the mixture was heated to reflux. After refluxing for 5 h, **1a'** (1.64 g, 4.4 mmol) was added to the mixture slowly and the reaction mixture was refluxed for another 36 h. After the mixture was cooled to room temperature, a few drops of water was added slowly. The solvent was removed under reduced pressure, and the residue was dissolved in dichloromethane (200 mL). The organic solution was washed with brine (3×150 mL) and dried over anhydrous Na_2SO_4. After removal of solvent, the residue was chromatographed on a silica gel column with a mixture of petroleum ether and ethyl acetate as the mobile phase to give pure **2b**(1.06 g, 73%) as a white solid: mp 105-106 °C; ^{1}H NMR (300 MHz, $CDCl_3$) δ 7.44 (t, *J* = 8.0 Hz, 1H), 7.25 (d, *J* = 8.6 Hz, 4H), 6.90 (d, *J* = 8.6 Hz, 4H), 6.76 (d, *J* = 4.6 Hz, 2H), 6.74 (d, *J* = 1.6 Hz, 2H), 6.65 (d, *J* = 1.6 Hz, 2H), 4.98 (s, 4H), 3.80 (s, 6H), 3.55 (s, 6H); ^{13}C NMR (75 MHz, $CDCl_3$) δ 166.6, 159.8, 158.3, 155.5, 140.2, 138.9, 129.3, 127.4, 114.2, 108.0, 107.9, 98.8, 70.0, 55.3, 36.1; IR (KBr) ν 1576, 1543, 1418 cm^{-1}; MS (EI) *m/z* (%): 724 (3), 723 (5), 722 (5), 721 (8), 720 (4), 719 $[M]^+$ (5), 603 (1), 602 (5), 601 (2), 600 (10) 598 (5), 122 (9), 121 (100). Anal. Calcd. for $C_{33}H_{31}Br_2N_5O_4$: C, 54.94; H, 4.33; N, 9.71. Found: C, 54.73; H, 4.29; N, 9.51.

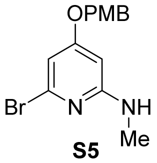

S5

4-((4-Methoxybenzyl)oxy)-6-Bromo-2-methylaminopyridine S5: An autoclave equipped with a magnetic stir bar was charged with 2,6-dibromo-4-((4-methoxybenzyl)oxy)pyridine **1a'** (3.73 g, 10 mmol), methylamine hydrochloride (0.81 g, 12 mmol), CuI (380 mg, 2 mmol), L-proline (460 mg, 2 mmol), K_2CO_3 (8.28 g, 60 mmol), DMSO (30 mL) and water (3 mL). Then it was heated to 50 °C for 48 h, and then cooled to room temperature. The mixture was poured into a mixture of 100 mL water and extracted by ethyl acetate (3 × 200 mL). The combined organic solution was washed with brine (3 × 250mL) and dried over anhydrous Na_2SO_4. After removal of solvent, the residue was chromatographed on a silica gel column with a mixture of dichloromethane and ethyl acetate as the mobile phase to give pure **1a** (2 g, 62%) as a white solid: mp 130-131 °C; ^{1}H NMR (300 MHz, $CDCl_3$) δ 7.31 (d, J = 8.6 Hz, 2H), 6.91 (d, J = 8.6 Hz, 2H), 6.43 (d, J = 1.8 Hz, 1H), 5.78 (d, J = 1.8 Hz, 1H), 4.96 (brs, 3H), 3.81 (s, 3H), 2.84 (d, J = 4.6 Hz, 3H); ^{13}C NMR (75 MHz, $CDCl_3$) δ 167.5, 160.9, 159.8, 140.9, 129.4, 127.7, 114.2, 104.0, 89.2, 69.9, 55.3, 29.2; IR (KBr) ν 3302, 1609, 1566, 1517, 1413 cm^{-1}; MS (EI) m/z (%): 325 (1), 324 (10), 323 (2), 322 [M]$^+$ (10), 122 (10), 121 (100). Anal. Calcd. for $C_{14}H_{15}BrN_2O_2$: C, 52.03; H, 4.68; N, 8.67. Found: C, 51.97; H, 4.68; N, 8.56.

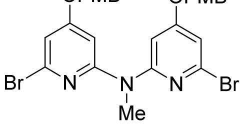

4

***N*-Methyl-bis(6-bromo-4-((4-methoxybenzyl)oxy)pyridin-2-yl)amine 4:** To a solution of **4-((4-**Methoxybenzyl)oxy)-6-Bromo-2-methylaminopyridine **S5** (1.29 g, 4 mmol)) in dry 1,4-dioxane (50

mL) at room temperature was added NaH (0.48 g, 20 mmol) slowly and the mixture was heated to reflux. After refluxing for 5 h, **1a'** (1.64 g, 4.4 mmol) was added to the mixture slowly and the reaction mixture was refluxed for another 36 h. After the mixture was cooled to room temperature, a few drops of water was added slowly. The solvent was removed under reduced pressure, and the residue was dissolved in dichloromethane (200 mL). The organic solution was washed with brine (3×150 mL) and dried over anhydrous Na_2SO_4. After removal of solvent, the residue was chromatographed on a silica gel column with a mixture of petroleum ether and ethyl acetate as the mobile phase to give pure **4** (1.79 g, 73%) as a white solid: mp 96-97 °C; ^{1}H NMR (300 MHz, $CDCl_3$) δ 7.30 (d, *J* = 8.6 Hz, 4H), 6.90 (d, *J* = 8.6 Hz, 4H), 6.78 (d, *J* = 1.7 Hz, 2H), 6.72 (d, *J* = 1.7 Hz, 2H), 4.97 (s, 4H), 3.80 (s, 6H), 3.52 (s, 3H); ^{13}C NMR (75 MHz, $CDCl_3$) δ 167.0, 159.9, 157.7, 140.2, 129.5, 127.3, 114.2, 108.8, 99.4, 70.3, 55.3, 36.3; IR (KBr) ν 1577, 1546 cm^{-1}; MS (EI) *m*/*z* (%): 617 (2), 615 (4), 613 $[M]^+$ (1), 496 (5), 494 (10), 492 (5), 122 (9), 121 (100). Anal. Calcd. for $C_{27}H_{25}Br_2N_3O_4$: C, 52.70; H, 4.10; N, 6.83. Found: C, 52.71; H, 4.19; N, 7.05.

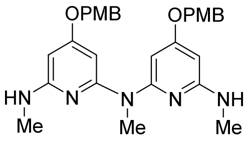

5

***N*-Methyl-bis(4-((4-methoxybenzyl)oxy)-6-methylaminopyridin-2-yl)amine 5:** An autoclave equipped with a magnetic stir bar was charged with *N*-Methyl-bis(6-bromo-4-((4-methoxybenzyl)oxy)pyridin-2-yl)amine **4** (3.08 g, 5 mmol), methylamine hydrochloride (1.35 g, 40 mmol), CuI (380 mg, 2 mmol), L-proline (460 mg, 2 mmol), K_2CO_3 (4.16 g, 30 mmol)，DMSO (30 mL) and water (3 mL). Then it was heated to 80 °C for 24 h, and then cooled to room temperature. The mixture was poured into a mixture of 100 mL water and extracted by ethyl acetate (3 × 200 mL). The combined organic solution was washed with brine (3 × 250mL) and dried over

anhydrous Na_2SO_4. After removal of solvent, the residue was chromatographed on a basic alumina column with a mixture of dichloromethane and ethyl acetate as the mobile phase to give pure **1a** (2.15g, 83%) as a white solid: mp 139-140 °C; ^{1}H NMR (300 MHz, $CDCl_3$) δ 7.30 (d, *J* = 8.6 Hz, 4H), 6.88 (d, *J* = 8.6 Hz, 4H), 6.17 (d, *J* = 1.5 Hz, 2H), 5.56 (d, *J* = 1.5 Hz, 2H), 4.90 (s, 4H), 4.43 (brs, 2H), 3.77 (s, 6H), 3.45 (s, 3H), 2.84 (d, *J* = 5.2Hz, 6H); ^{13}C NMR (75 MHz, $CDCl_3$) δ 167.6, 160.1, 159.5, 158.4, 129.3, 128.6, 114.0, 91.1, 84.6, 69.4, 55.3, 35.9, 29.3; IR (KBr) ν 3450, 3425, 1607, 1513 cm^{-1}; MS (MALDI-TOF) *m/z* (%) 516 $[M+H]^+$ (100). Anal. Calcd. for $C_{29}H_{33}N_5O_4$: C, 67.55; H, 6.45; N, 13.58. Found: C, 67.45; H, 6.51; N, 13.55.

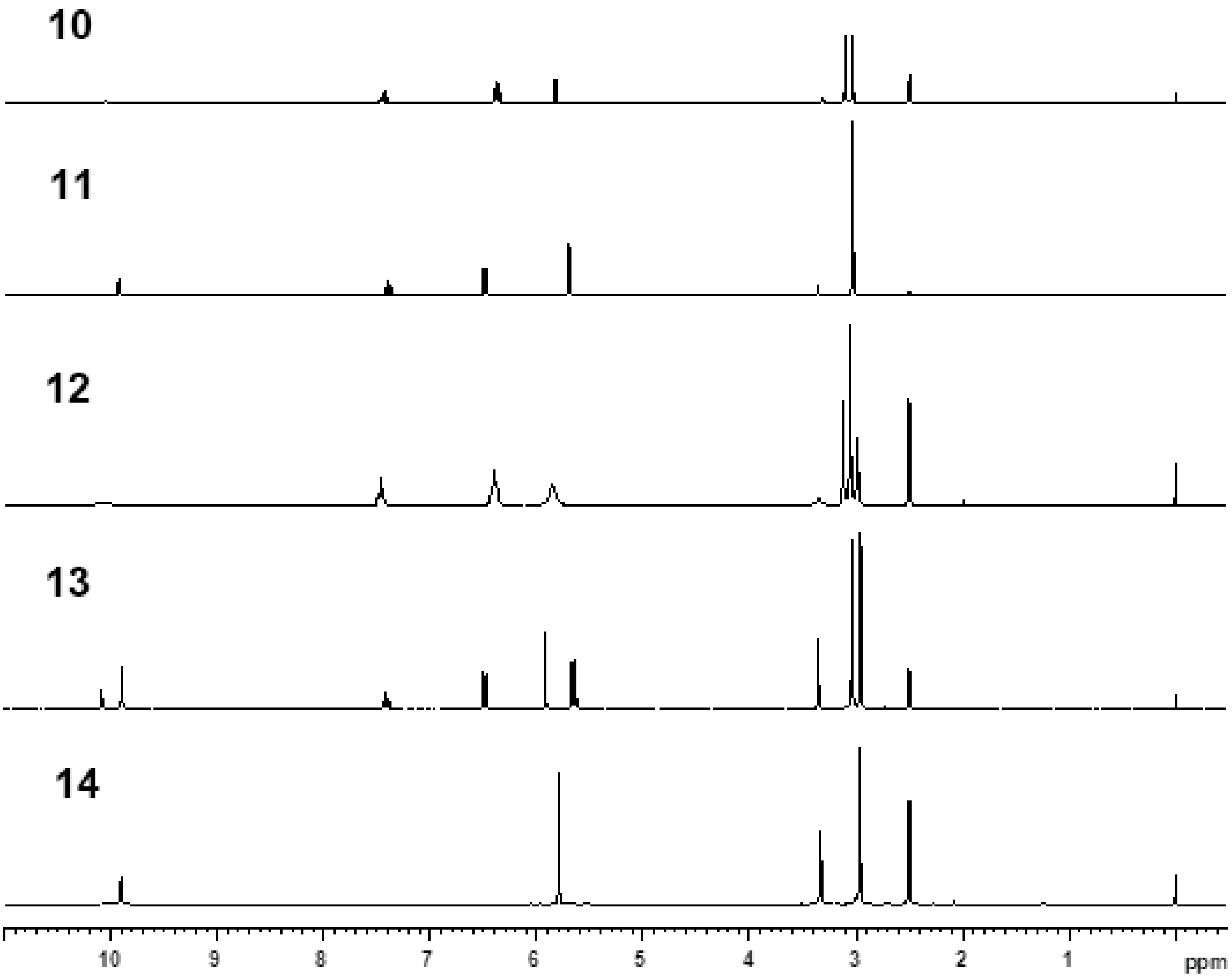

Figure S2. ^{1}H NMR spectra of hydroxyl-substituted azacalix[4]pyridines **10-14** in *d*^{6}-DMSO at 298K.

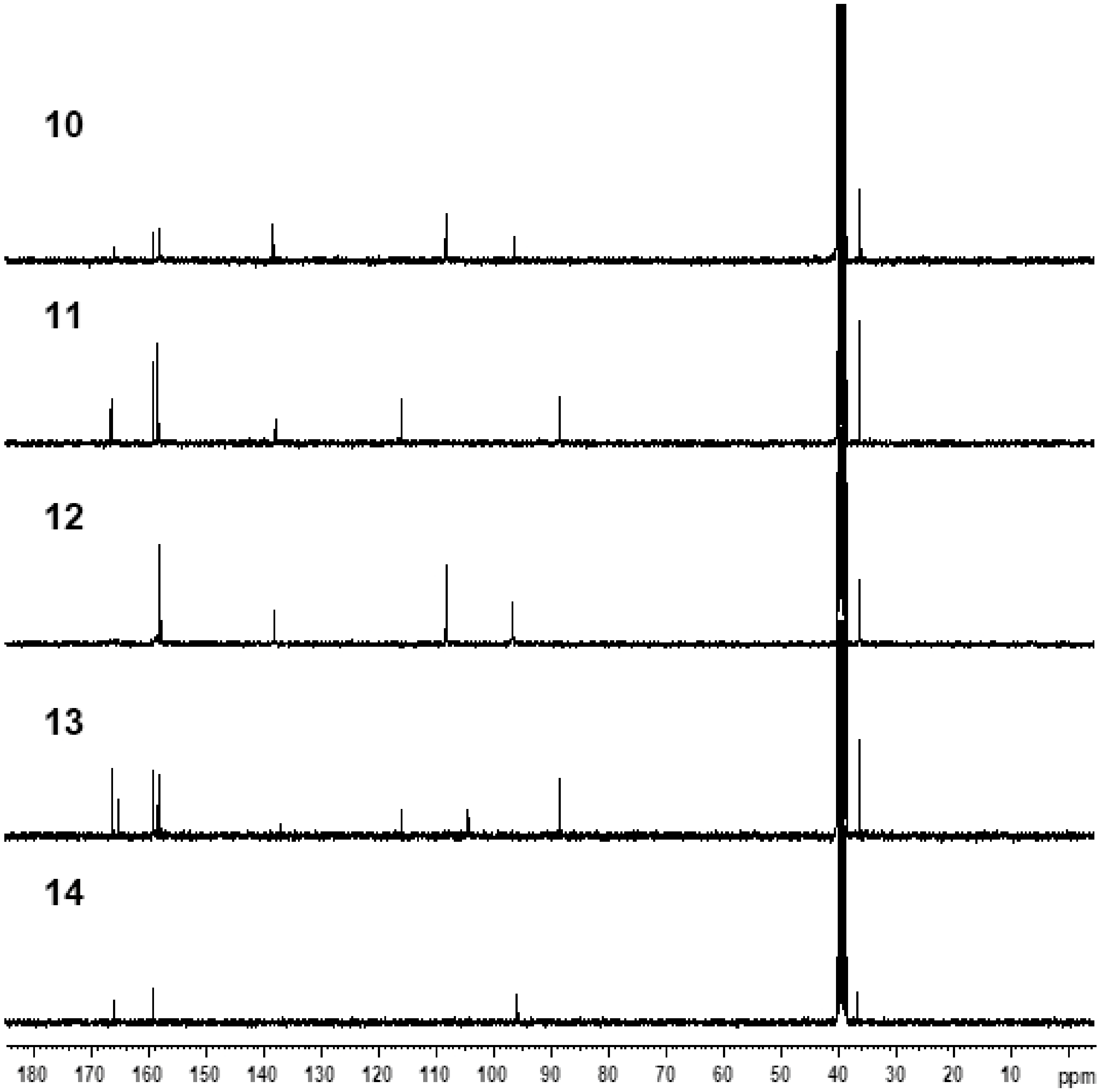

Figure S3. ^{13}C NMR spectra of hydroxyl-substituted azacalix[4]pyridines **10-14** in d^6-DMSO at 298K.

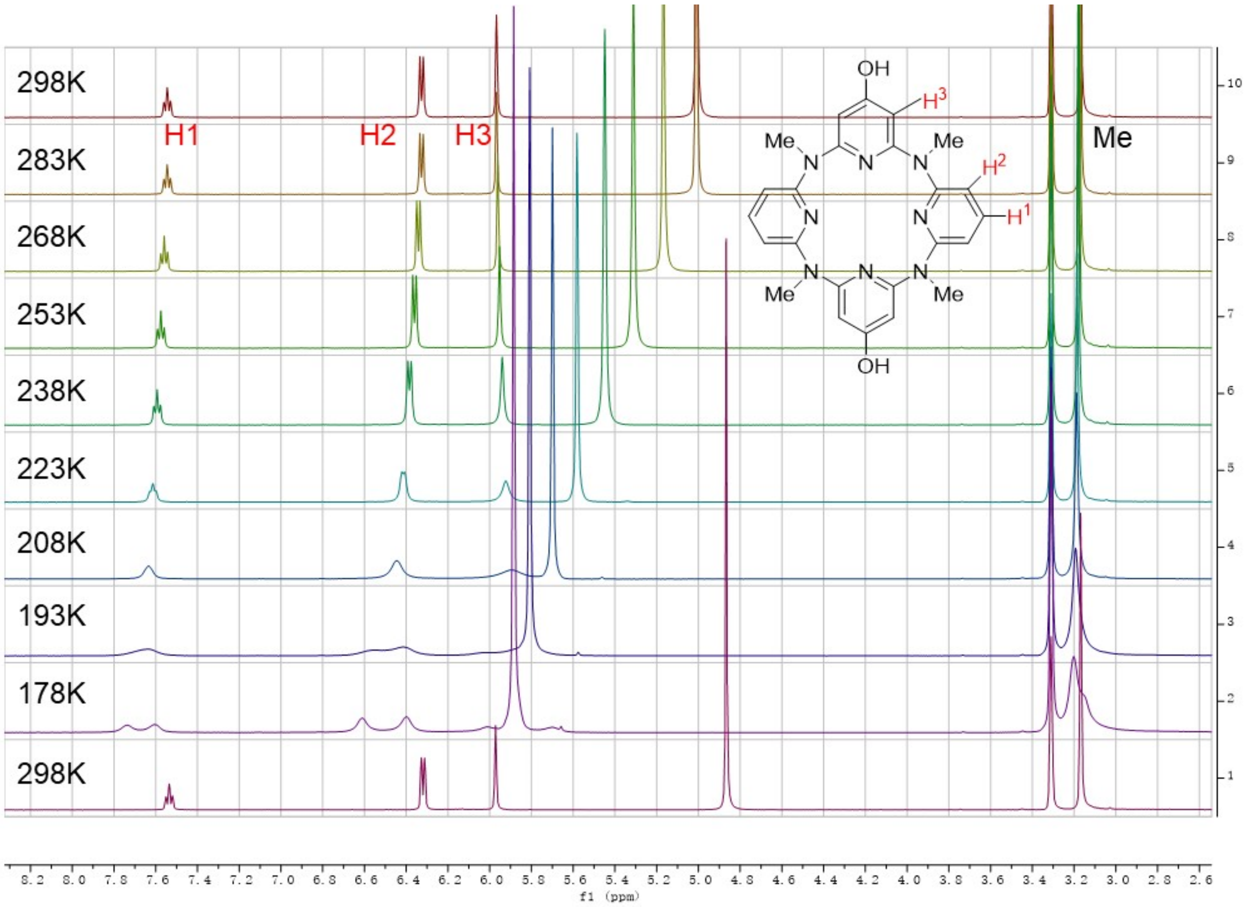

Figure S4. ^{1}H NMR of **11** at variable temperature.

Copies of ^{1}H and ^{13}C NMR Spectra of products

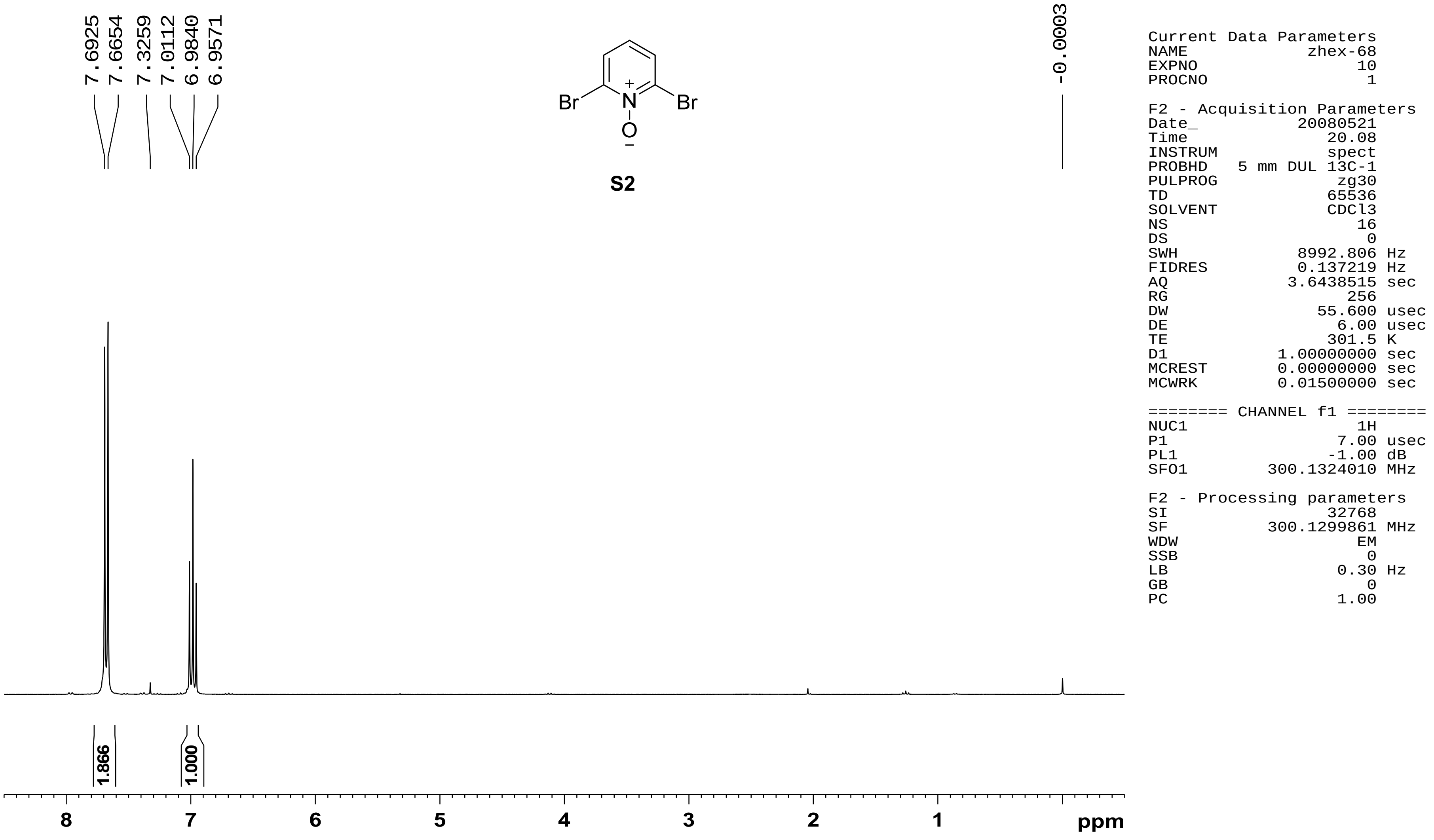

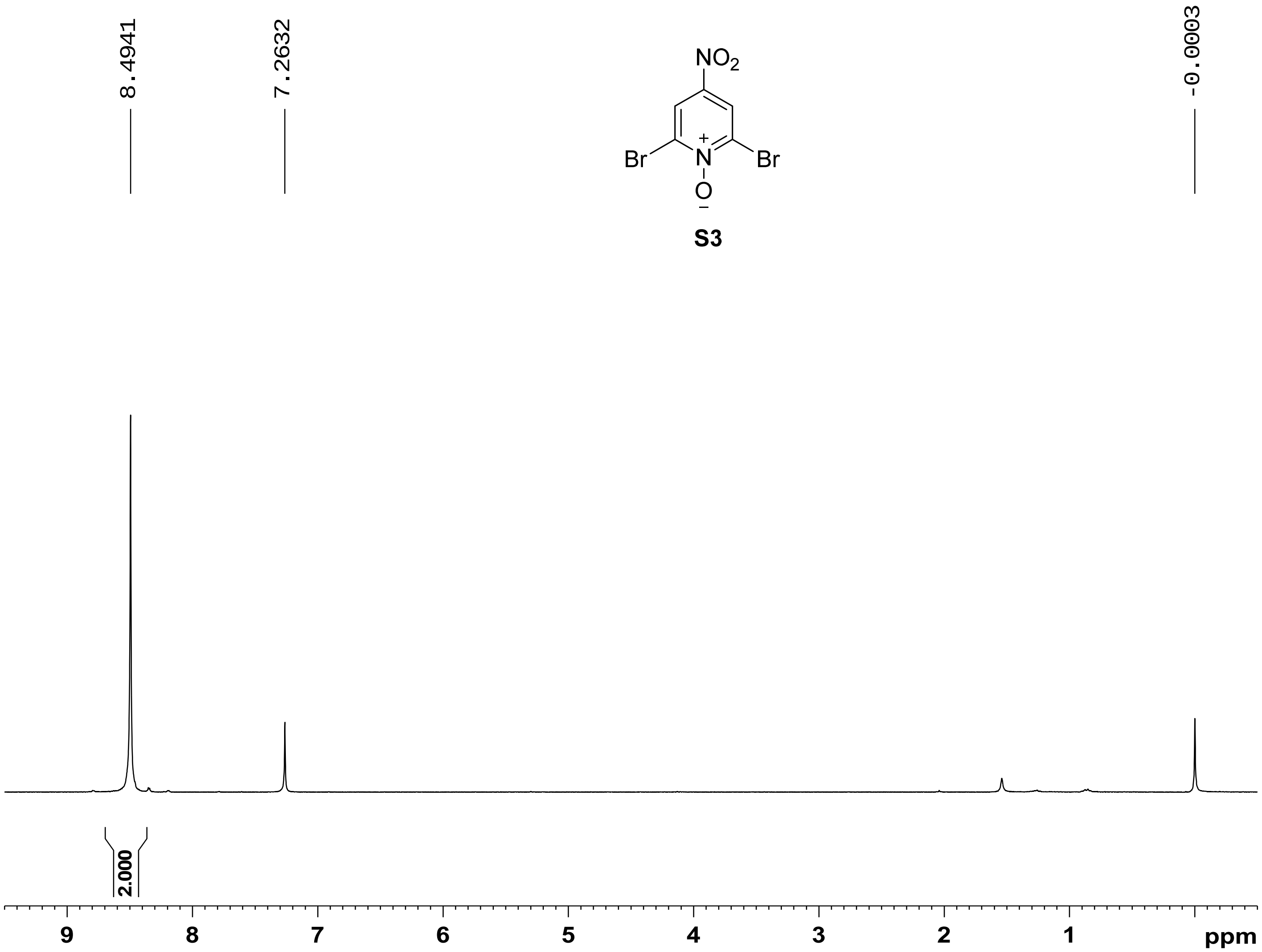


```
Current Data Parameters
NAME              zhex-70
EXPNO                  10
PROCNO                  1

F2 - Acquisition Parameters
Date_            20080521
Time                19.58
INSTRUM             spect
PROBHD   5 mm DUL 13C-1
PULPROG              zg30
TD                  65536
SOLVENT             CDCl3
NS                     16
DS                      0
SWH              8992.806 Hz
FIDRES           0.137219 Hz
AQ              3.6438515 sec
RG                  574.7
DW                 55.600 usec
DE                   6.00 usec
TE                  301.5 K
D1             1.00000000 sec
MCREST         0.00000000 sec
MCWRK          0.01500000 sec

======== CHANNEL f1 ========
NUC1                   1H
P1                   7.00 usec
PL1                 -1.00 dB
SFO1          300.1324010 MHz

F2 - Processing parameters
SI                  32768
SF            300.1300048 MHz
WDW                    EM
SSB                     0
LB                   0.30 Hz
GB                      0
PC                   1.00
```

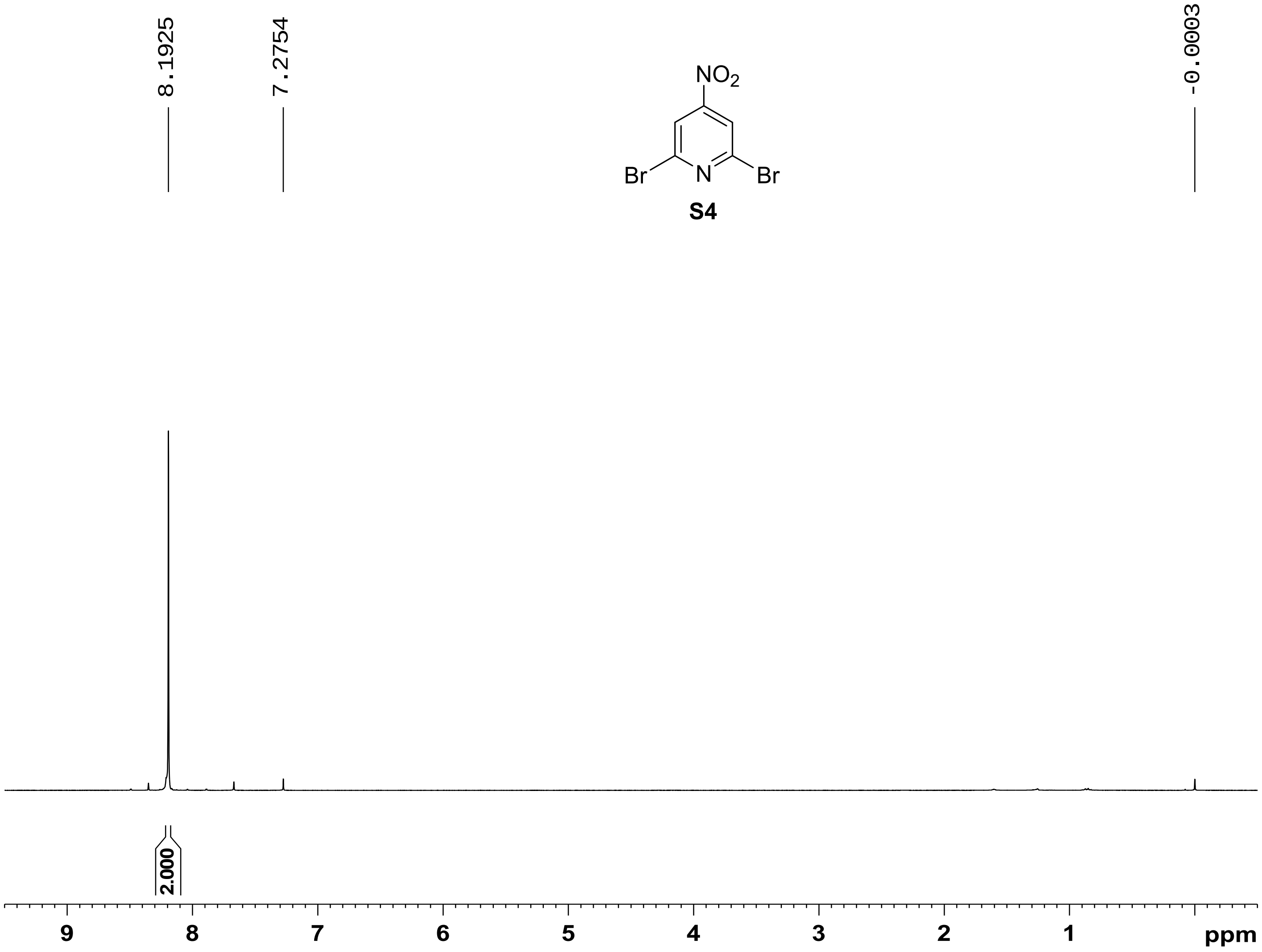

S4

```
Current Data Parameters
NAME            zhex-66
EXPNO                10
PROCNO                1

F2 - Acquisition Parameters
Date_          20080521
Time              19.40
INSTRUM           spect
PROBHD   5 mm DUL 13C-1
PULPROG            zg30
TD                65536
SOLVENT           CDCl3
NS                   16
DS                    0
SWH            8992.806 Hz
FIDRES         0.137219 Hz
AQ            3.6438515 sec
RG                  362
DW               55.600 usec
DE                 6.00 usec
TE                300.6 K
D1           1.00000000 sec
MCREST       0.00000000 sec
MCWRK        0.01500000 sec

======== CHANNEL f1 ========
NUC1                 1H
P1                 7.00 usec
PL1               -1.00 dB
SFO1        300.1324010 MHz

F2 - Processing parameters
SI                32768
SF          300.1300013 MHz
WDW                  EM
SSB                   0
LB                 0.30 Hz
GB                    0
PC                 1.00
```

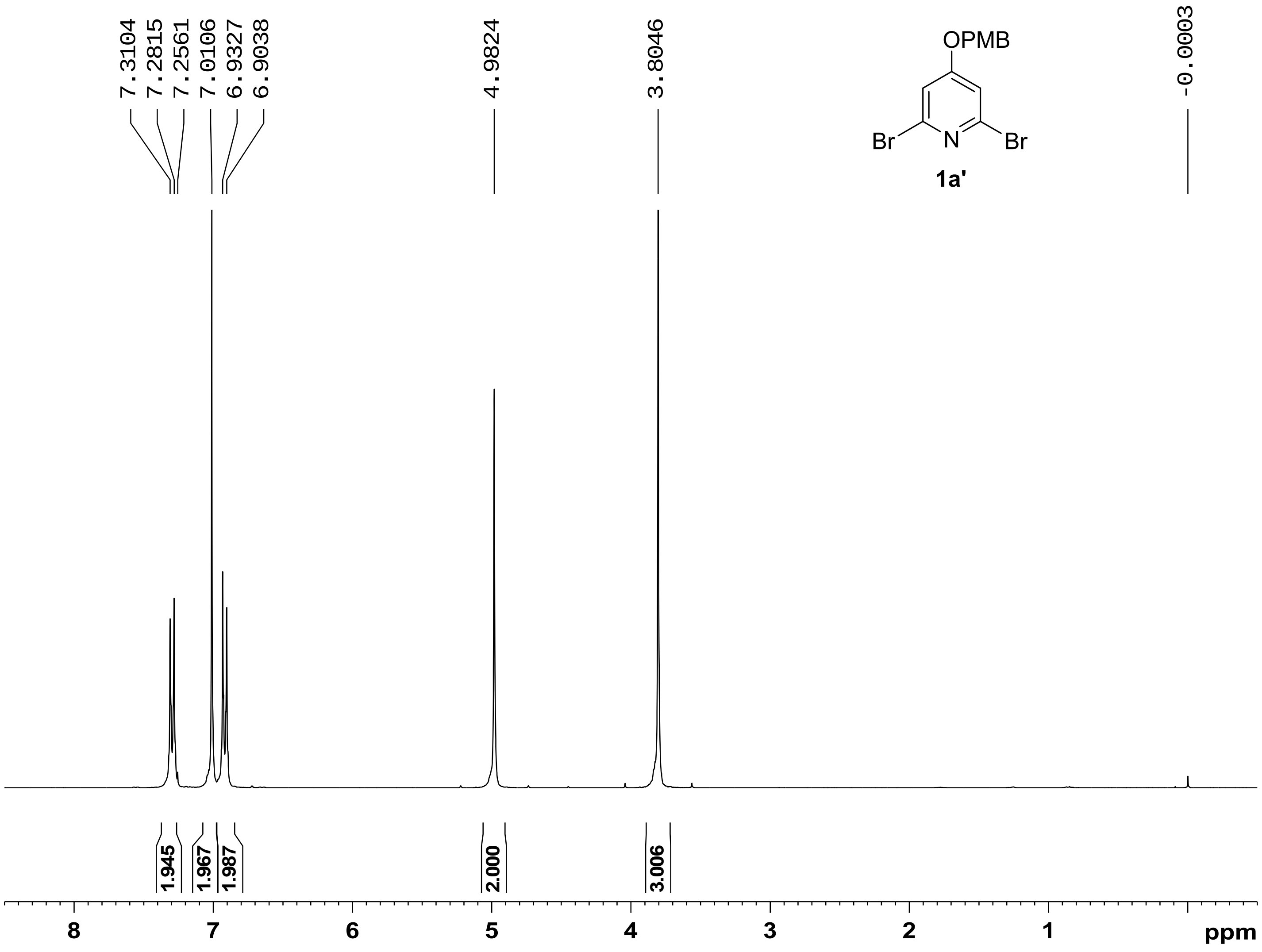


```
Current Data Parameters
NAME             zhex-179
EXPNO                  30
PROCNO                  1

F2 - Acquisition Parameters
Date_            20081003
Time                20.46
INSTRUM             spect
PROBHD   5 mm DUL 13C-1
PULPROG              zg30
TD                  65536
SOLVENT             CDCl3
NS                      9
DS                      0
SWH              8992.806 Hz
FIDRES           0.137219 Hz
AQ              3.6438515 sec
RG                   71.8
DW                 55.600 usec
DE                   6.00 usec
TE                  299.8 K
D1             1.00000000 sec
MCREST         0.00000000 sec
MCWRK          0.01500000 sec

======== CHANNEL f1 ========
NUC1                   1H
P1                   7.00 usec
PL1                 -1.00 dB
SFO1          300.1324010 MHz

F2 - Processing parameters
SI                  32768
SF            300.1300071 MHz
WDW                    EM
SSB                     0
LB                   0.30 Hz
GB                      0
PC                   1.00
```

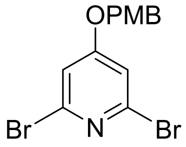

1a'

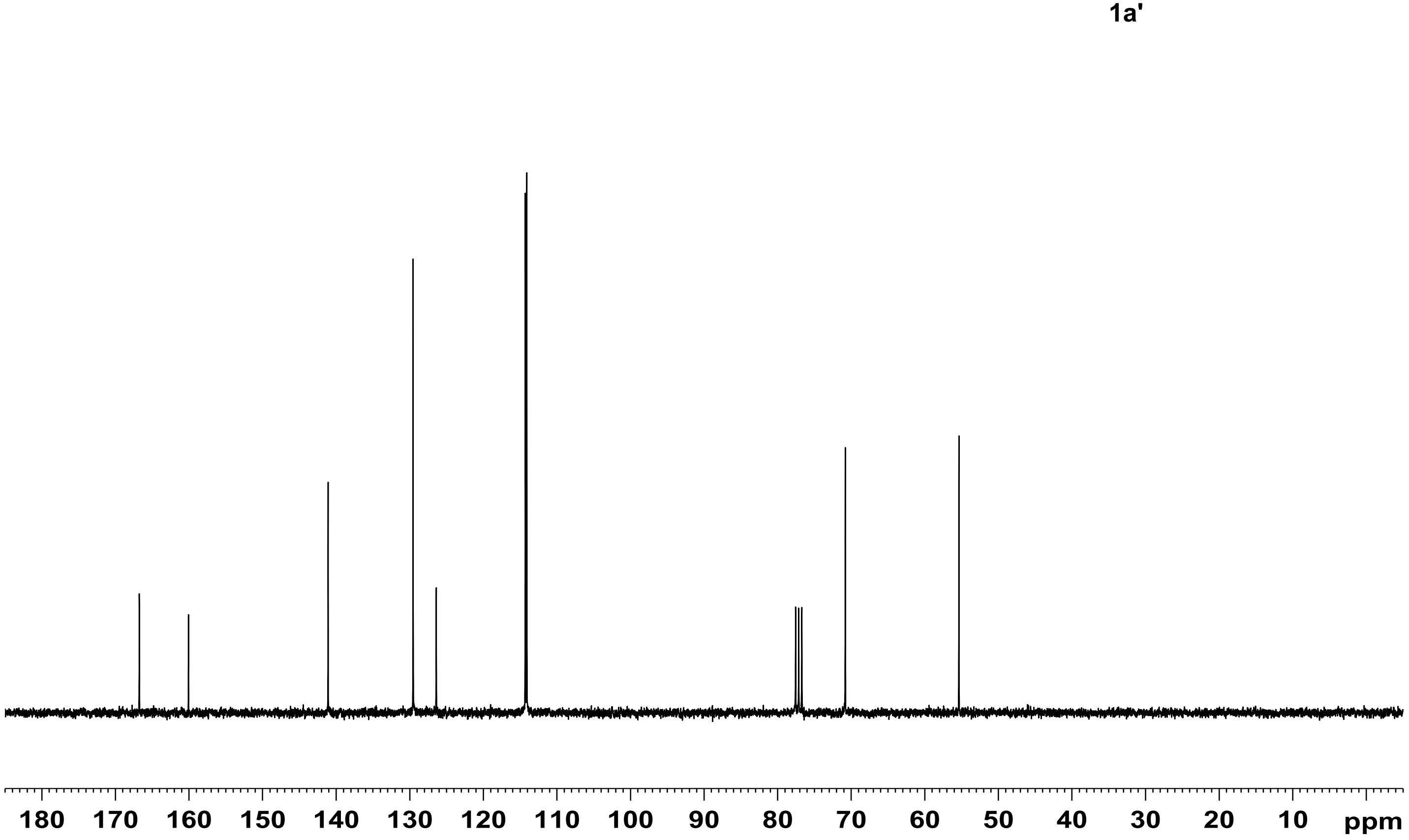


```
Current Data Parameters
NAME             zhex-179
EXPNO                  31
PROCNO                  1

F2 - Acquisition Parameters
Date_            20081003
Time                20.49
INSTRUM             spect
PROBHD   5 mm DUL 13C-1
PULPROG           zgpg30
TD                 65536
SOLVENT            CDCl3
NS                    38
DS                     4
SWH            17985.611 Hz
FIDRES          0.274439 Hz
AQ             1.8219508 sec
RG                 812.7
DW                27.800 usec
DE                  6.00 usec
TE                 300.8 K
D1            2.00000000 sec
d11           0.03000000 sec
DELTA         1.89999998 sec
MCREST        0.00000000 sec
MCWRK         0.01500000 sec

======== CHANNEL f1 ========
NUC1                 13C
P1                 12.50 usec
PL1                 2.00 dB
SFO1          75.4752953 MHz

======== CHANNEL f2 ========
CPDPRG2          waltz16
NUC2                  1H
PCPD2              80.00 usec
PL2                -1.00 dB
PL12               20.16 dB
PL13               16.98 dB
SFO2         300.1312005 MHz

F2 - Processing parameters
SI                 32768
SF            75.4677490 MHz
WDW                   EM
SSB                    0
LB                  1.00 Hz
GB                     0
PC                  1.40
```

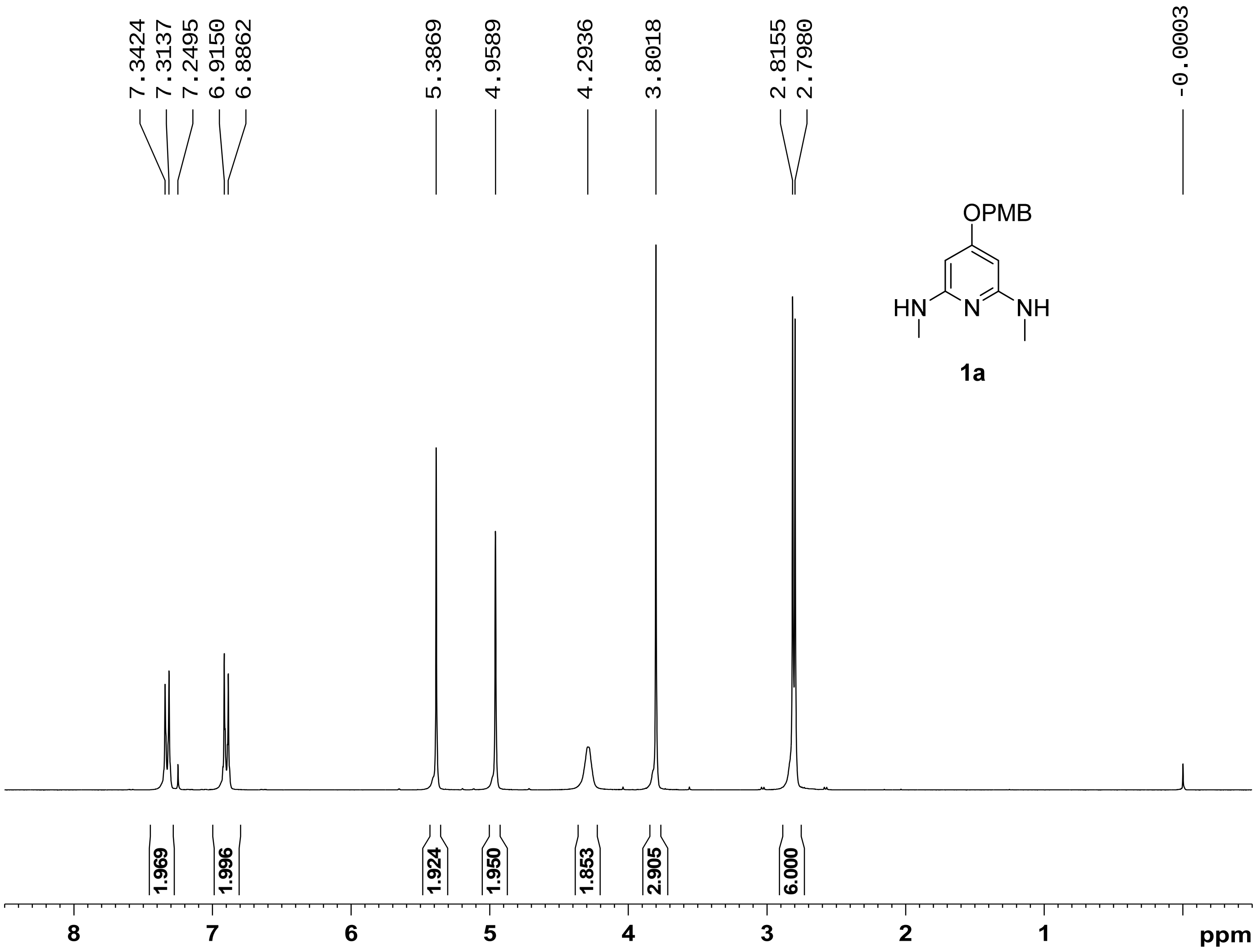


```
Current Data Parameters
NAME              zhex-195
EXPNO                   10
PROCNO                   1

F2 - Acquisition Parameters
Date_             20081003
Time                 21.06
INSTRUM              spect
PROBHD   5 mm DUL 13C-1
PULPROG               zg30
TD                   65536
SOLVENT              CDCl3
NS                      16
DS                       0
SWH               8992.806 Hz
FIDRES            0.137219 Hz
AQ               3.6438515 sec
RG                     128
DW                  55.600 usec
DE                    6.00 usec
TE                   301.1 K
D1              1.00000000 sec
MCREST          0.00000000 sec
MCWRK           0.01500000 sec

======== CHANNEL f1 ========
NUC1                    1H
P1                    7.00 usec
PL1                  -1.00 dB
SFO1           300.1324010 MHz

F2 - Processing parameters
SI                   32768
SF             300.1300091 MHz
WDW                     EM
SSB                      0
LB                    0.30 Hz
GB                       0
PC                    1.00
```

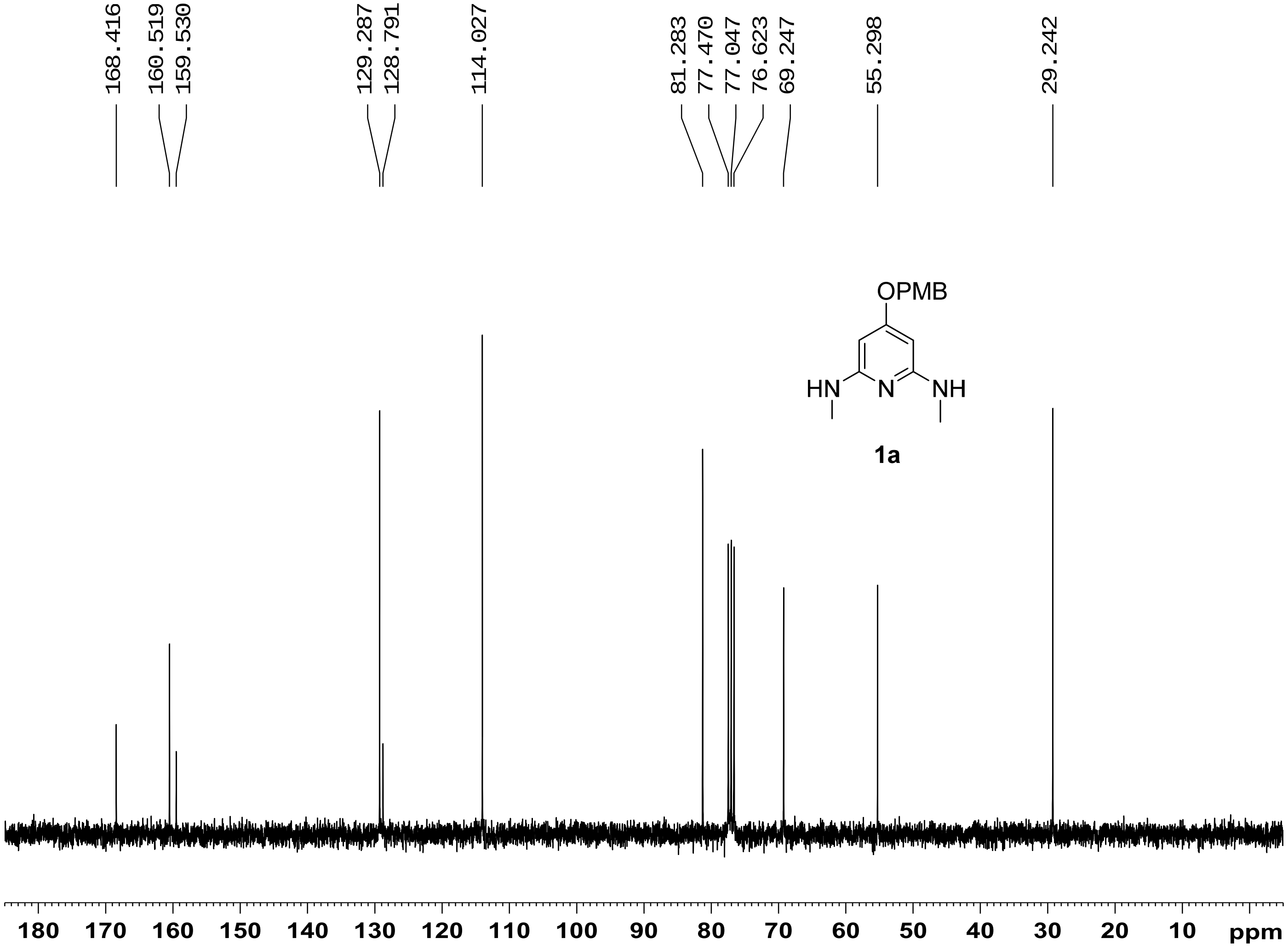


```
Current Data Parameters
NAME             zhex-195
EXPNO                  11
PROCNO                  1

F2 - Acquisition Parameters
Date_            20081003
Time                21.10
INSTRUM             spect
PROBHD   5 mm DUL 13C-1
PULPROG            zgpg30
TD                  65536
SOLVENT             CDCl3
NS                     53
DS                      4
SWH             17985.611 Hz
FIDRES           0.274439 Hz
AQ              1.8219508 sec
RG                  574.7
DW                 27.800 usec
DE                   6.00 usec
TE                  301.5 K
D1             2.00000000 sec
d11            0.03000000 sec
DELTA          1.89999998 sec
MCREST         0.00000000 sec
MCWRK          0.01500000 sec

======== CHANNEL f1 ========
NUC1                  13C
P1                  12.50 usec
PL1                  2.00 dB
SFO1           75.4752953 MHz

======== CHANNEL f2 ========
CPDPRG2           waltz16
NUC2                   1H
PCPD2               80.00 usec
PL2                 -1.00 dB
PL12                20.16 dB
PL13                16.98 dB
SFO2          300.1312005 MHz

F2 - Processing parameters
SI                  32768
SF             75.4677490 MHz
WDW                    EM
SSB                     0
LB                   1.00 Hz
GB                      0
PC                   1.40
```

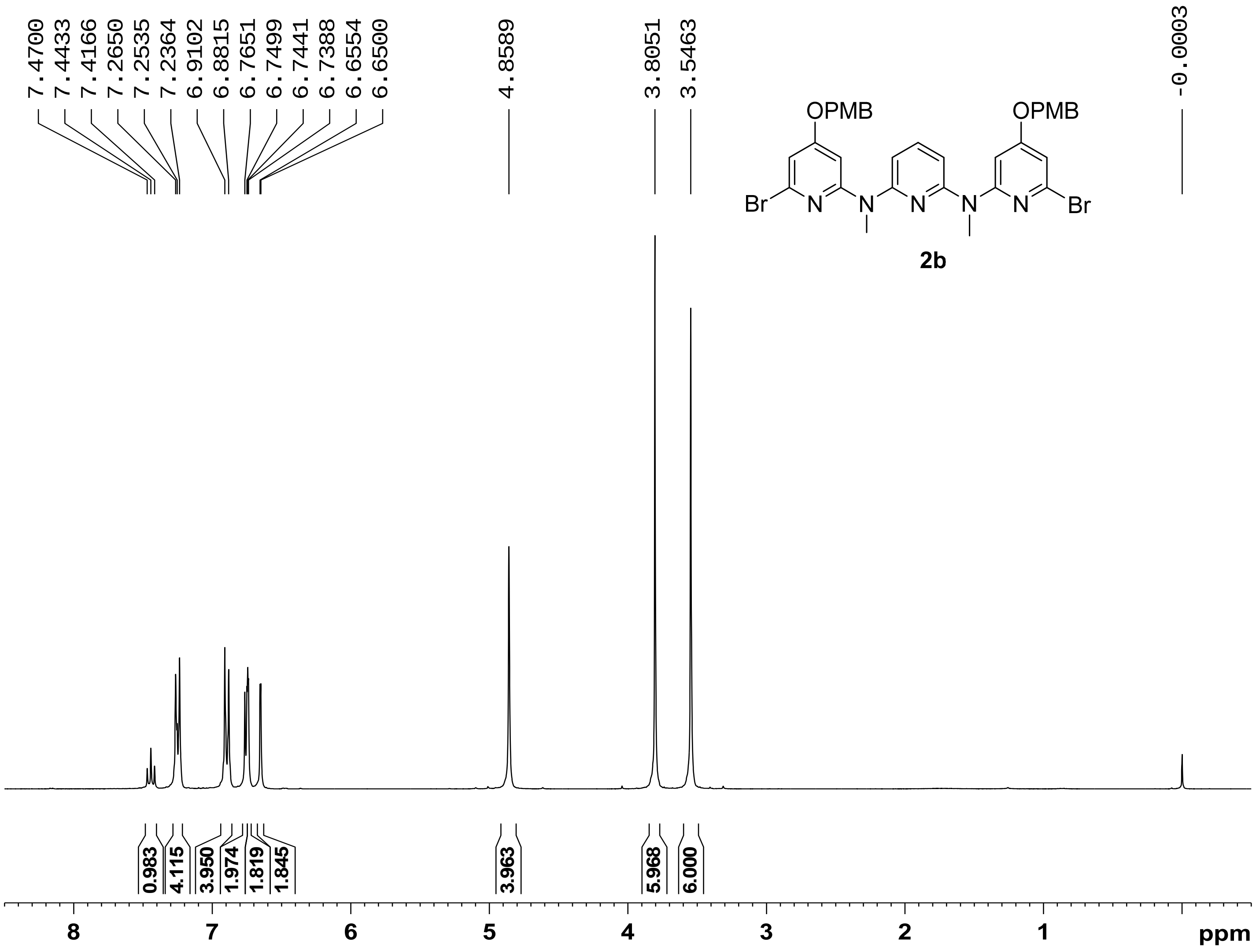


```
Current Data Parameters
NAME             zhex-182
EXPNO                  10
PROCNO                  1

F2 - Acquisition Parameters
Date_            20081003
Time                20.34
INSTRUM             spect
PROBHD   5 mm DUL 13C-1
PULPROG              zg30
TD                  65536
SOLVENT             CDCl3
NS                     16
DS                      0
SWH              8992.806 Hz
FIDRES           0.137219 Hz
AQ              3.6438515 sec
RG                  228.1
DW                 55.600 usec
DE                   6.00 usec
TE                  299.5 K
D1             1.00000000 sec
MCREST         0.00000000 sec
MCWRK          0.01500000 sec

======== CHANNEL f1 ========
NUC1                   1H
P1                   7.00 usec
PL1                 -1.00 dB
SFO1          300.1324010 MHz

F2 - Processing parameters
SI                  32768
SF            300.1300080 MHz
WDW                    EM
SSB                     0
LB                   0.30 Hz
GB                      0
PC                   1.00
```

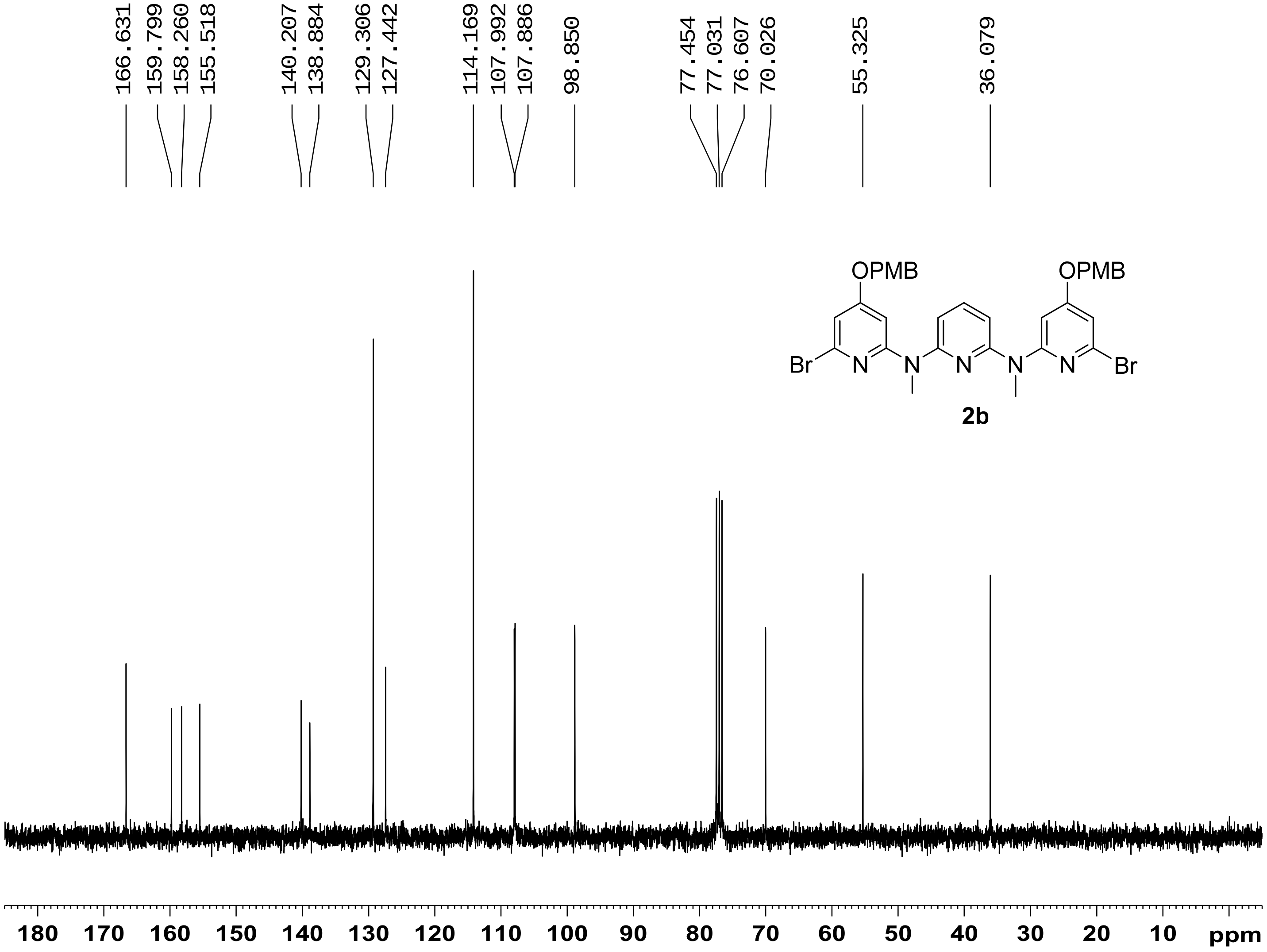


```
Current Data Parameters
NAME             zhex-182
EXPNO                  20
PROCNO                  1

F2 - Acquisition Parameters
Date_            20081003
Time                20.55
INSTRUM             spect
PROBHD   5 mm DUL 13C-1
PULPROG           zgpg30
TD                 65536
SOLVENT            CDCl3
NS                    77
DS                     4
SWH            17985.611 Hz
FIDRES          0.274439 Hz
AQ             1.8219508 sec
RG                 574.7
DW                27.800 usec
DE                  6.00 usec
TE                 301.8 K
D1            2.00000000 sec
d11           0.03000000 sec
DELTA         1.89999998 sec
MCREST        0.00000000 sec
MCWRK         0.01500000 sec

======== CHANNEL f1 ========
NUC1                 13C
P1                 12.50 usec
PL1                 2.00 dB
SFO1          75.4752953 MHz

======== CHANNEL f2 ========
CPDPRG2          waltz16
NUC2                  1H
PCPD2              80.00 usec
PL2                -1.00 dB
PL12               20.16 dB
PL13               16.98 dB
SFO2         300.1312005 MHz

F2 - Processing parameters
SI                 32768
SF            75.4677490 MHz
WDW                   EM
SSB                    0
LB                  1.00 Hz
GB                     0
PC                  1.40
```

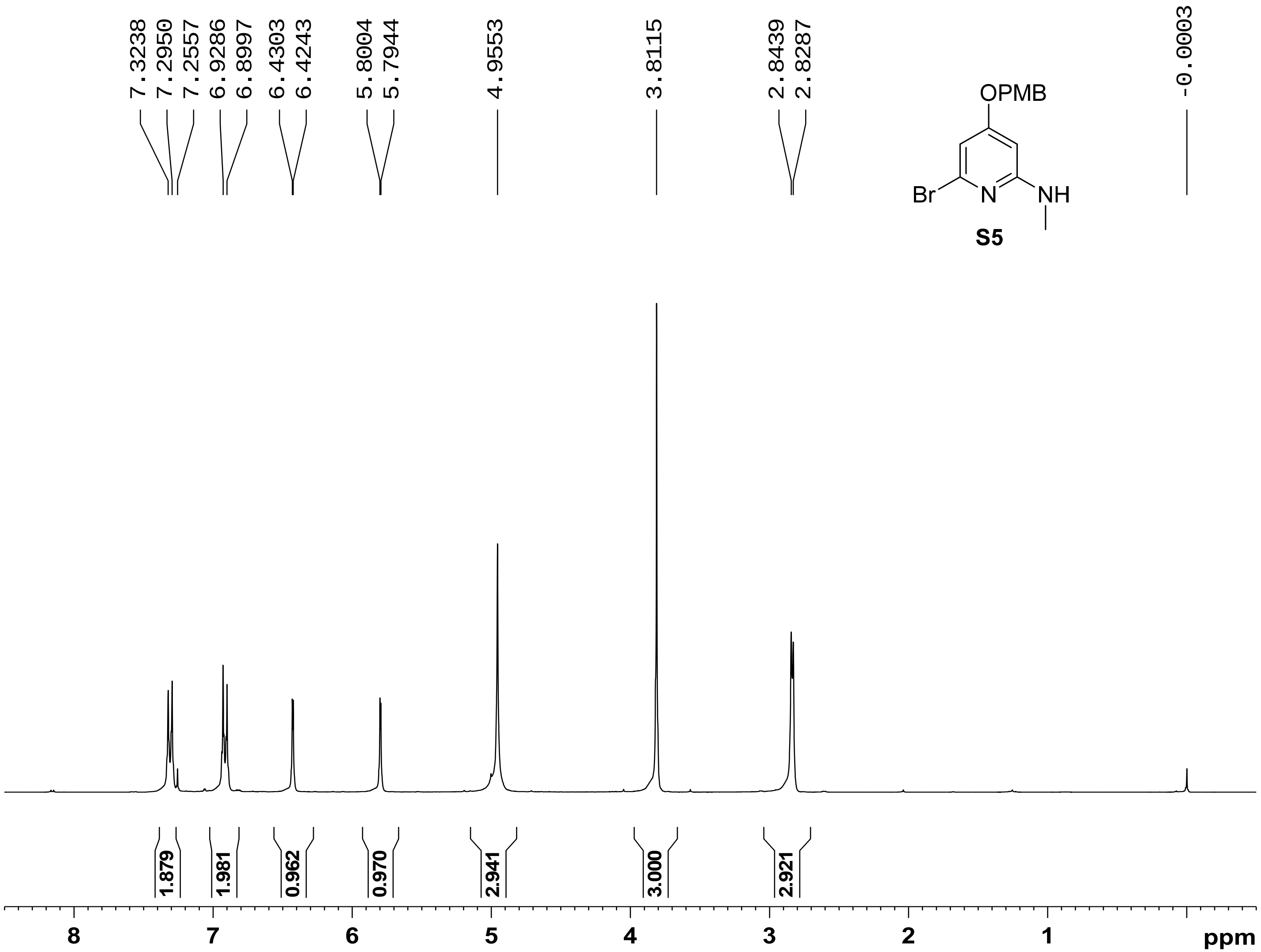

S5

```
Current Data Parameters
NAME             zhex-203
EXPNO                  10
PROCNO                  1

F2 - Acquisition Parameters
Date_            20081003
Time                19.52
INSTRUM             spect
PROBHD   5 mm DUL 13C-1
PULPROG              zg30
TD                  65536
SOLVENT             CDCl3
NS                     16
DS                      0
SWH              8992.806 Hz
FIDRES           0.137219 Hz
AQ              3.6438515 sec
RG                    128
DW                 55.600 usec
DE                   6.00 usec
TE                  300.0 K
D1             1.00000000 sec
MCREST         0.00000000 sec
MCWRK          0.01500000 sec

======== CHANNEL f1 ========
NUC1                   1H
P1                   7.00 usec
PL1                 -1.00 dB
SFO1          300.1324010 MHz

F2 - Processing parameters
SI                  32768
SF            300.1300071 MHz
WDW                    EM
SSB                     0
LB                   0.30 Hz
GB                      0
PC                   1.00
```

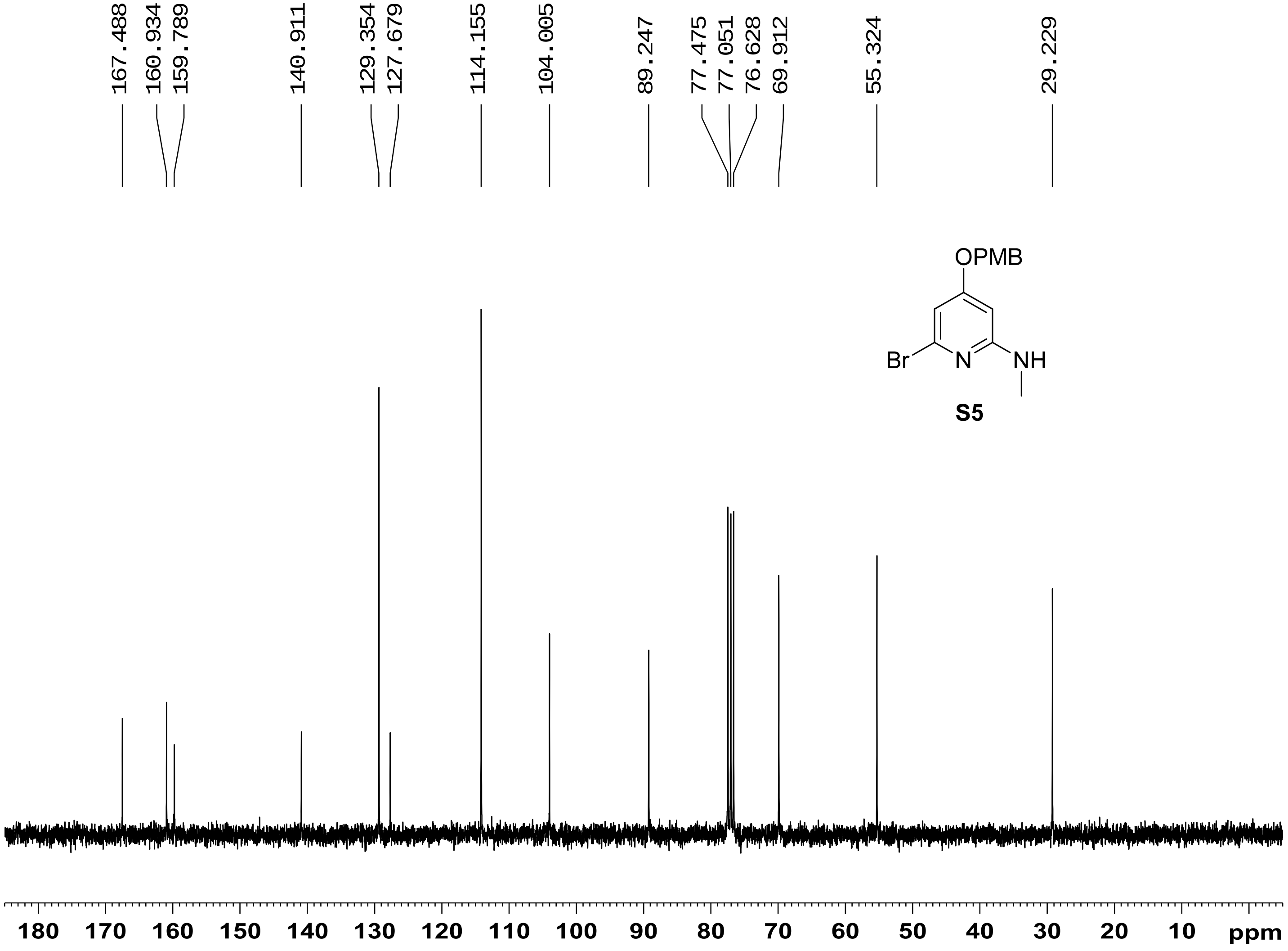

Current Data Parameters
NAME zhex-203
EXPNO 11
PROCNO 1

F2 - Acquisition Parameters
Date_ 20081003
Time 19.59
INSTRUM spect
PROBHD 5 mm DUL 13C-1
PULPROG zgpg30
TD 65536
SOLVENT CDCl3
NS 78
DS 4
SWH 17985.611 Hz
FIDRES 0.274439 Hz
AQ 1.8219508 sec
RG 574.7
DW 27.800 usec
DE 6.00 usec
TE 301.5 K
D1 2.00000000 sec
d11 0.03000000 sec
DELTA 1.89999998 sec
MCREST 0.00000000 sec
MCWRK 0.01500000 sec

======== CHANNEL f1 ========
NUC1 13C
P1 12.50 usec
PL1 2.00 dB
SFO1 75.4752953 MHz

======== CHANNEL f2 ========
CPDPRG2 waltz16
NUC2 1H
PCPD2 80.00 usec
PL2 -1.00 dB
PL12 20.16 dB
PL13 16.98 dB
SFO2 300.1312005 MHz

F2 - Processing parameters
SI 32768
SF 75.4677490 MHz
WDW EM
SSB 0
LB 1.00 Hz
GB 0
PC 1.40

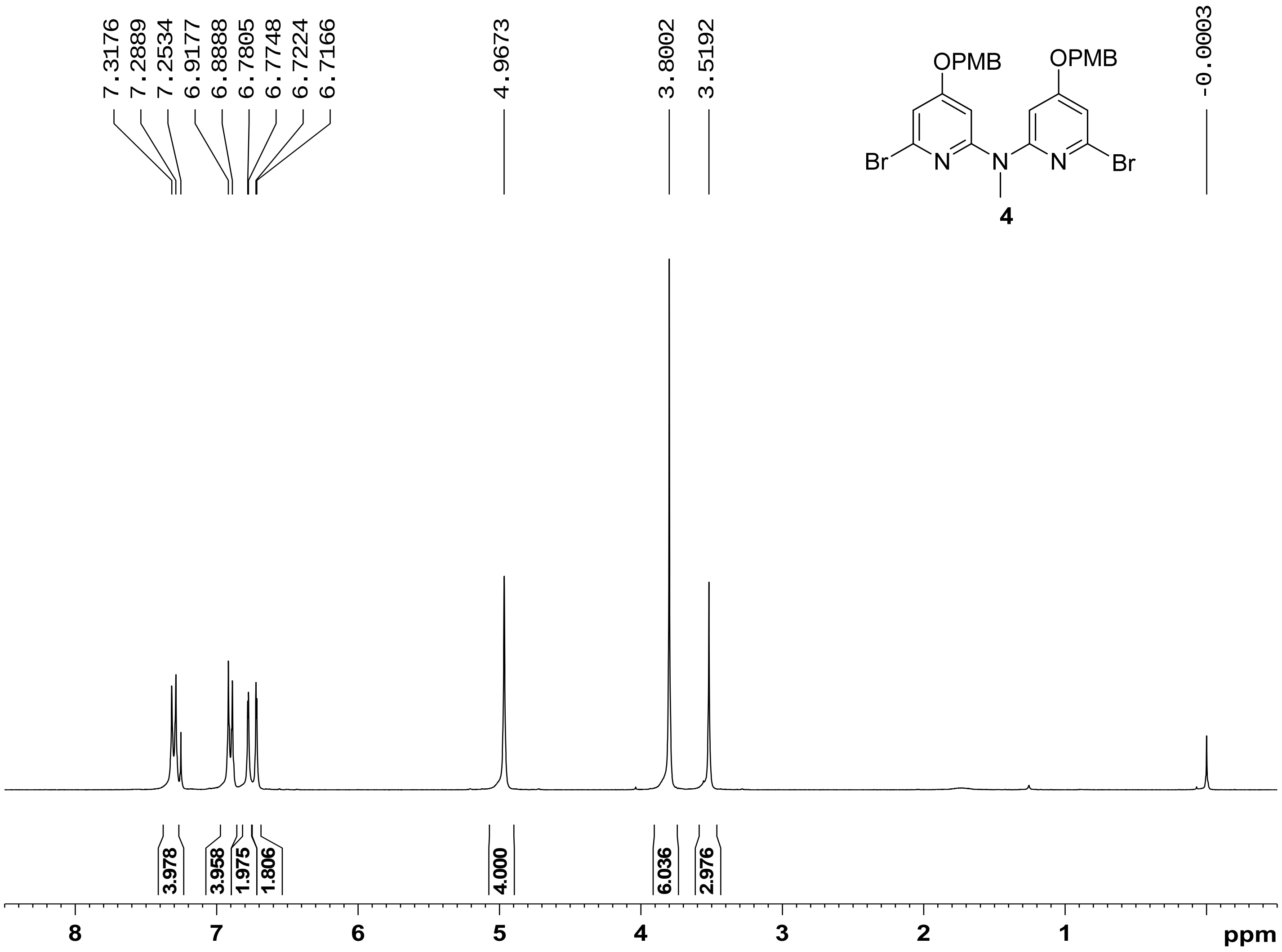


```
Current Data Parameters
NAME            zhex-205
EXPNO                 12
PROCNO                 1

F2 - Acquisition Parameters
Date_           20081003
Time               19.46
INSTRUM            spect
PROBHD   5 mm DUL 13C-1
PULPROG             zg30
TD                 65536
SOLVENT            CDCl3
NS                    16
DS                     0
SWH             8992.806 Hz
FIDRES          0.137219 Hz
AQ             3.6438515 sec
RG                   362
DW                55.600 usec
DE                  6.00 usec
TE                 301.0 K
D1            1.00000000 sec
MCREST        0.00000000 sec
MCWRK         0.01500000 sec

======== CHANNEL f1 ========
NUC1                  1H
P1                  7.00 usec
PL1                -1.00 dB
SFO1         300.1324010 MHz

F2 - Processing parameters
SI                 32768
SF           300.1300078 MHz
WDW                   EM
SSB                    0
LB                  0.30 Hz
GB                     0
PC                  1.00
```

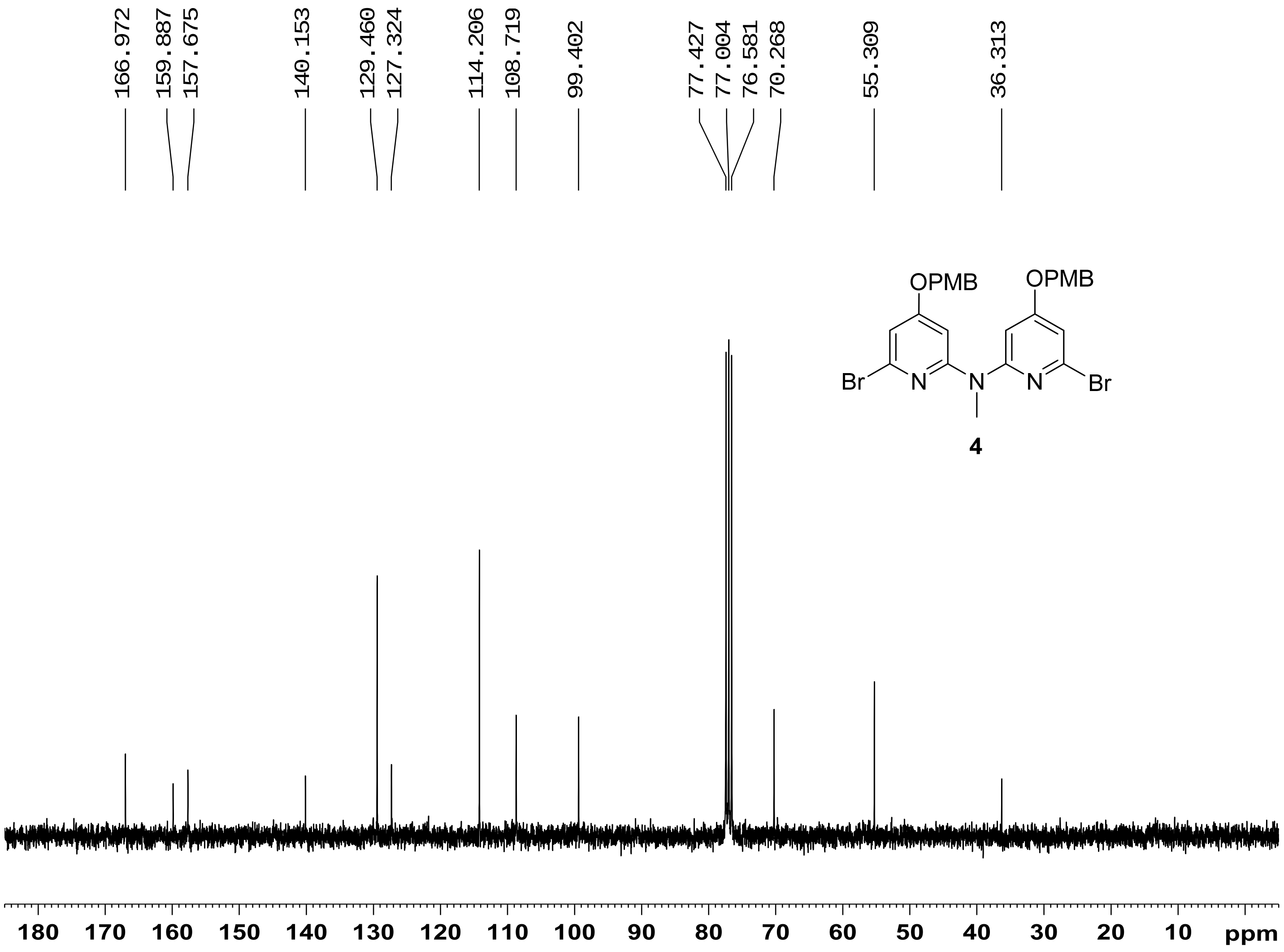


```
Current Data Parameters
NAME              zhex-205
EXPNO                   11
PROCNO                   1

F2 - Acquisition Parameters
Date_             20081003
Time                 19.37
INSTRUM              spect
PROBHD   5 mm DUL 13C-1
PULPROG            zgpg30
TD                   65536
SOLVENT              CDCl3
NS                     162
DS                       4
SWH              17985.611 Hz
FIDRES            0.274439 Hz
AQ               1.8219508 sec
RG                   724.1
DW                  27.800 usec
DE                    6.00 usec
TE                   301.6 K
D1              2.00000000 sec
d11             0.03000000 sec
DELTA           1.89999998 sec
MCREST          0.00000000 sec
MCWRK           0.01500000 sec

======== CHANNEL f1 ========
NUC1                   13C
P1                   12.50 usec
PL1                   2.00 dB
SFO1            75.4752953 MHz

======== CHANNEL f2 ========
CPDPRG2            waltz16
NUC2                    1H
PCPD2                80.00 usec
PL2                  -1.00 dB
PL12                 20.16 dB
PL13                 16.98 dB
SFO2           300.1312005 MHz

F2 - Processing parameters
SI                   32768
SF              75.4677490 MHz
WDW                     EM
SSB                      0
LB                    1.00 Hz
GB                       0
PC                    1.40
```

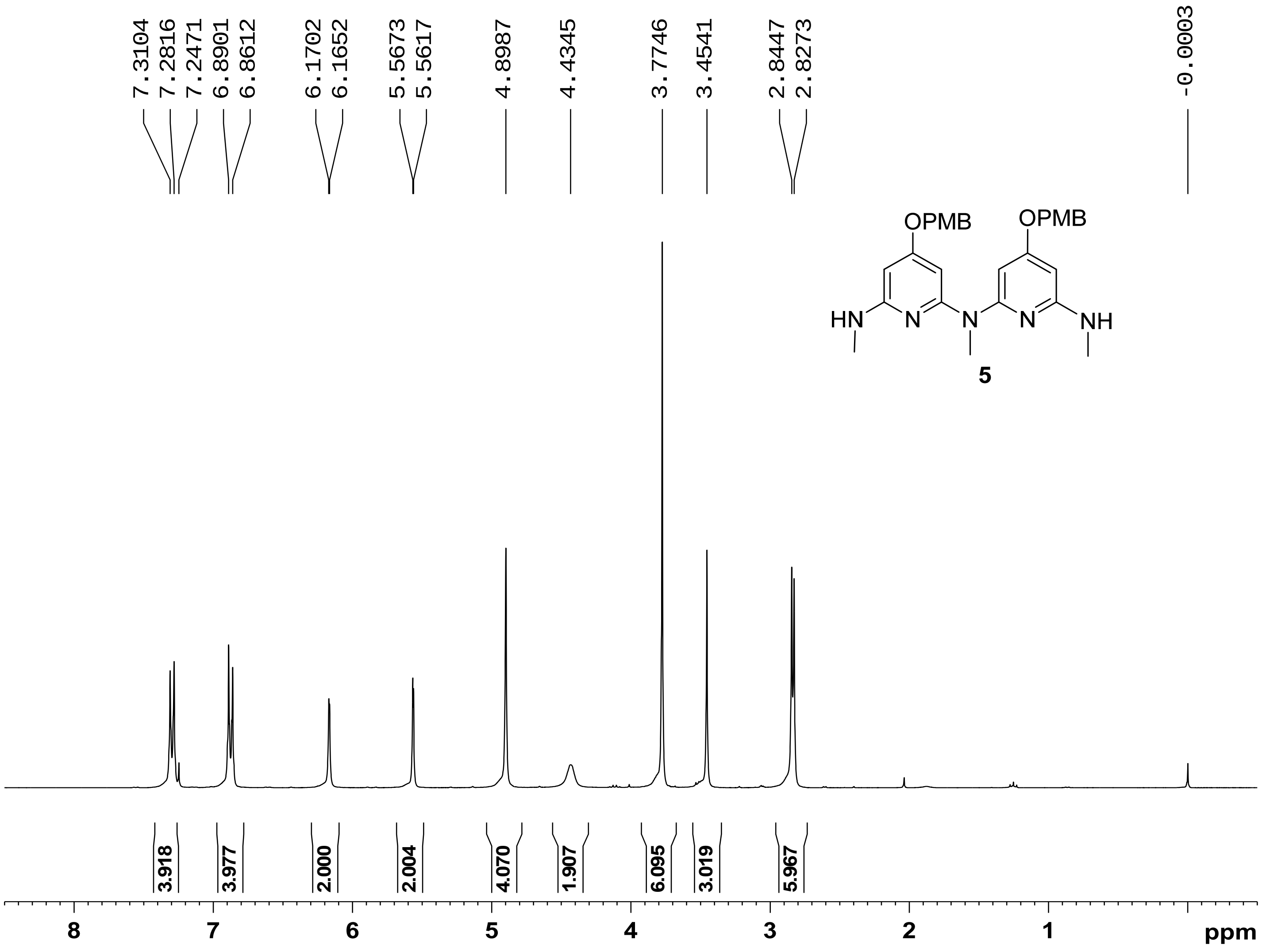


```
Current Data Parameters
NAME             zhex-301
EXPNO                  10
PROCNO                  1

F2 - Acquisition Parameters
Date_            20080818
Time                19.42
INSTRUM             spect
PROBHD   5 mm DUL 13C-1
PULPROG              zg30
TD                  65536
SOLVENT             CDCl3
NS                     16
DS                      0
SWH             8992.806 Hz
FIDRES          0.137219 Hz
AQ             3.6438515 sec
RG                    128
DW                 55.600 usec
DE                   6.00 usec
TE                  299.9 K
D1             1.00000000 sec
MCREST         0.00000000 sec
MCWRK          0.01500000 sec

======== CHANNEL f1 ========
NUC1                   1H
P1                   7.00 usec
PL1                 -1.00 dB
SFO1          300.1324010 MHz

F2 - Processing parameters
SI                  32768
SF            300.1300096 MHz
WDW                    EM
SSB                     0
LB                   0.30 Hz
GB                      0
PC                   1.00
```

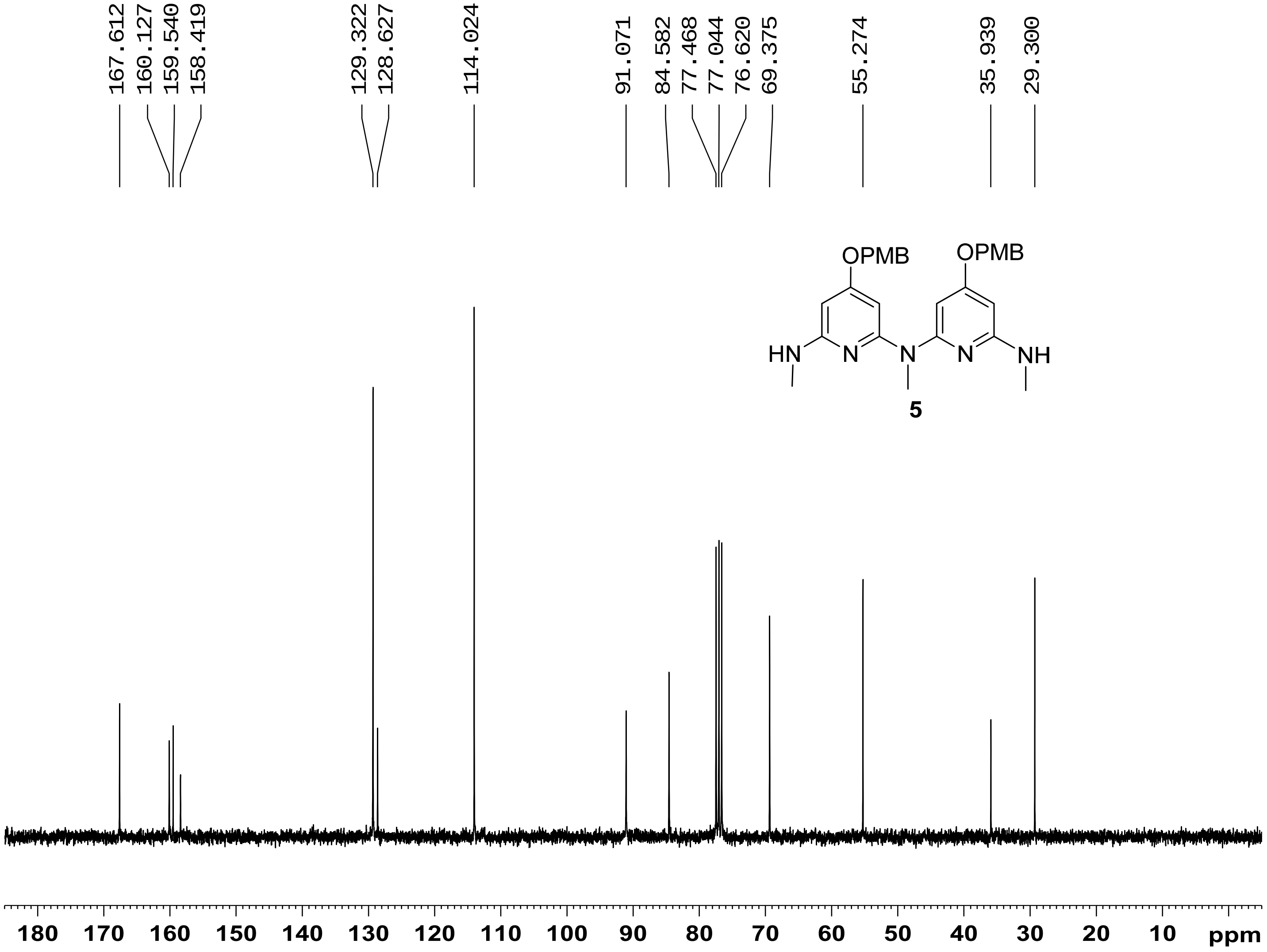


```
Current Data Parameters
NAME           zhex-301
EXPNO                11
PROCNO                1

F2 - Acquisition Parameters
Date_          20080818
Time              19.46
INSTRUM           spect
PROBHD   5 mm DUL 13C-1
PULPROG          zgpg30
TD                65536
SOLVENT           CDCl3
NS                  163
DS                    4
SWH           17985.611 Hz
FIDRES         0.274439 Hz
AQ            1.8219508 sec
RG                456.1
DW               27.800 usec
DE                 6.00 usec
TE                300.9 K
D1           2.00000000 sec
d11          0.03000000 sec
DELTA        1.89999998 sec
MCREST       0.00000000 sec
MCWRK        0.01500000 sec

======== CHANNEL f1 ========
NUC1                13C
P1                12.50 usec
PL1                2.00 dB
SFO1         75.4752953 MHz

======== CHANNEL f2 ========
CPDPRG2         waltz16
NUC2                 1H
PCPD2             80.00 usec
PL2               -1.00 dB
PL12              20.16 dB
PL13              16.98 dB
SFO2        300.1312005 MHz

F2 - Processing parameters
SI                32768
SF           75.4677490 MHz
WDW                  EM
SSB                   0
LB                 1.00 Hz
GB                    0
PC                 1.40
```

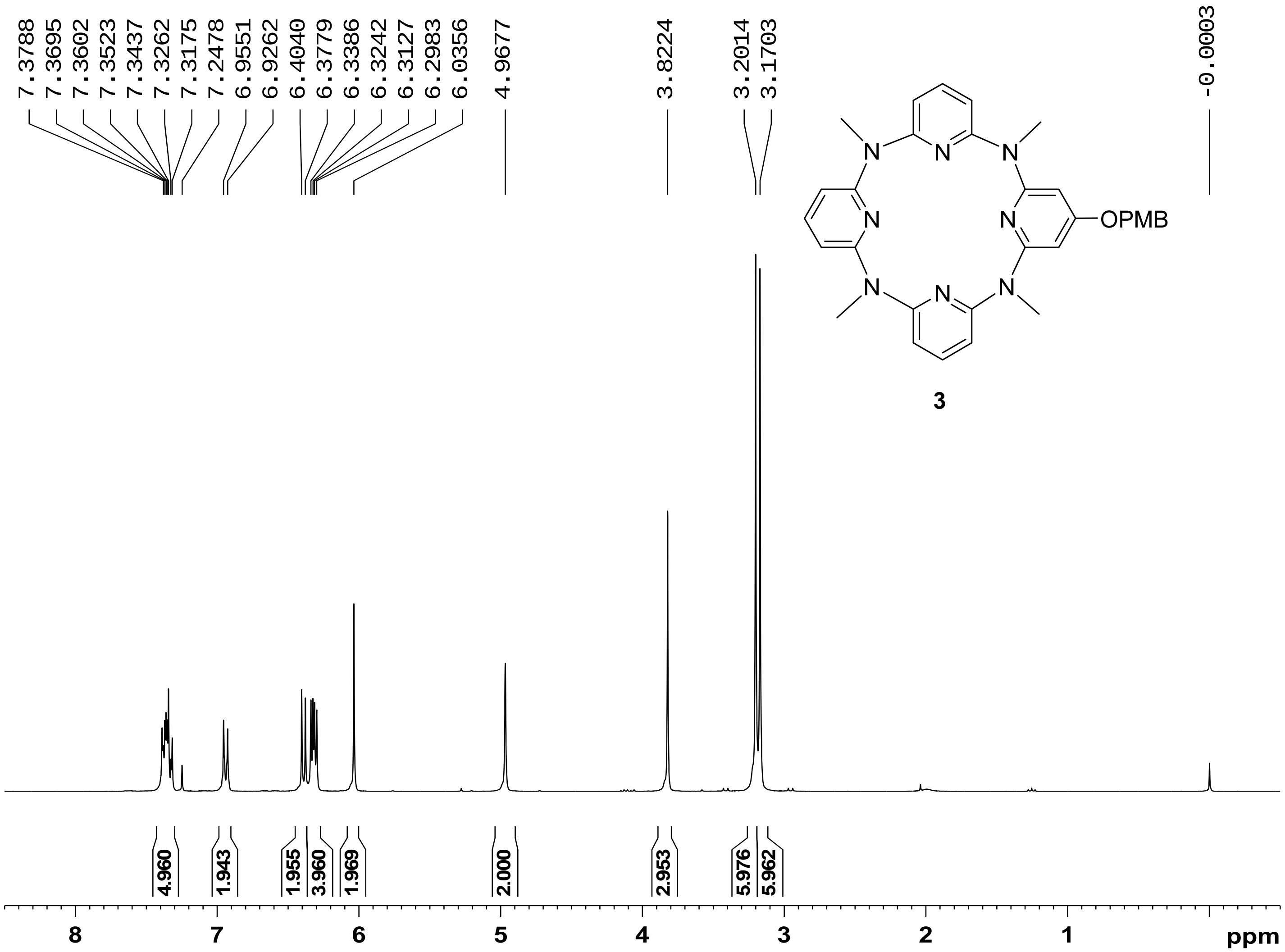

Current Data Parameters

NAME	zhex-201
EXPNO	70
PROCNO	1

F2 - Acquisition Parameters

Date_	20080821
Time	19.24
INSTRUM	spect
PROBHD	5 mm DUL 13C-1
PULPROG	zg30
TD	65536
SOLVENT	CDCl3
NS	16
DS	0
SWH	8992.806 Hz
FIDRES	0.137219 Hz
AQ	3.6438515 sec
RG	143.7
DW	55.600 usec
DE	6.00 usec
TE	299.7 K
D1	1.00000000 sec
MCREST	0.00000000 sec
MCWRK	0.01500000 sec

======== CHANNEL f1 ========

NUC1	1H
P1	7.00 usec
PL1	-1.00 dB
SFO1	300.1324010 MHz

F2 - Processing parameters

SI	32768
SF	300.1300096 MHz
WDW	EM
SSB	0
LB	0.30 Hz
GB	0
PC	1.00

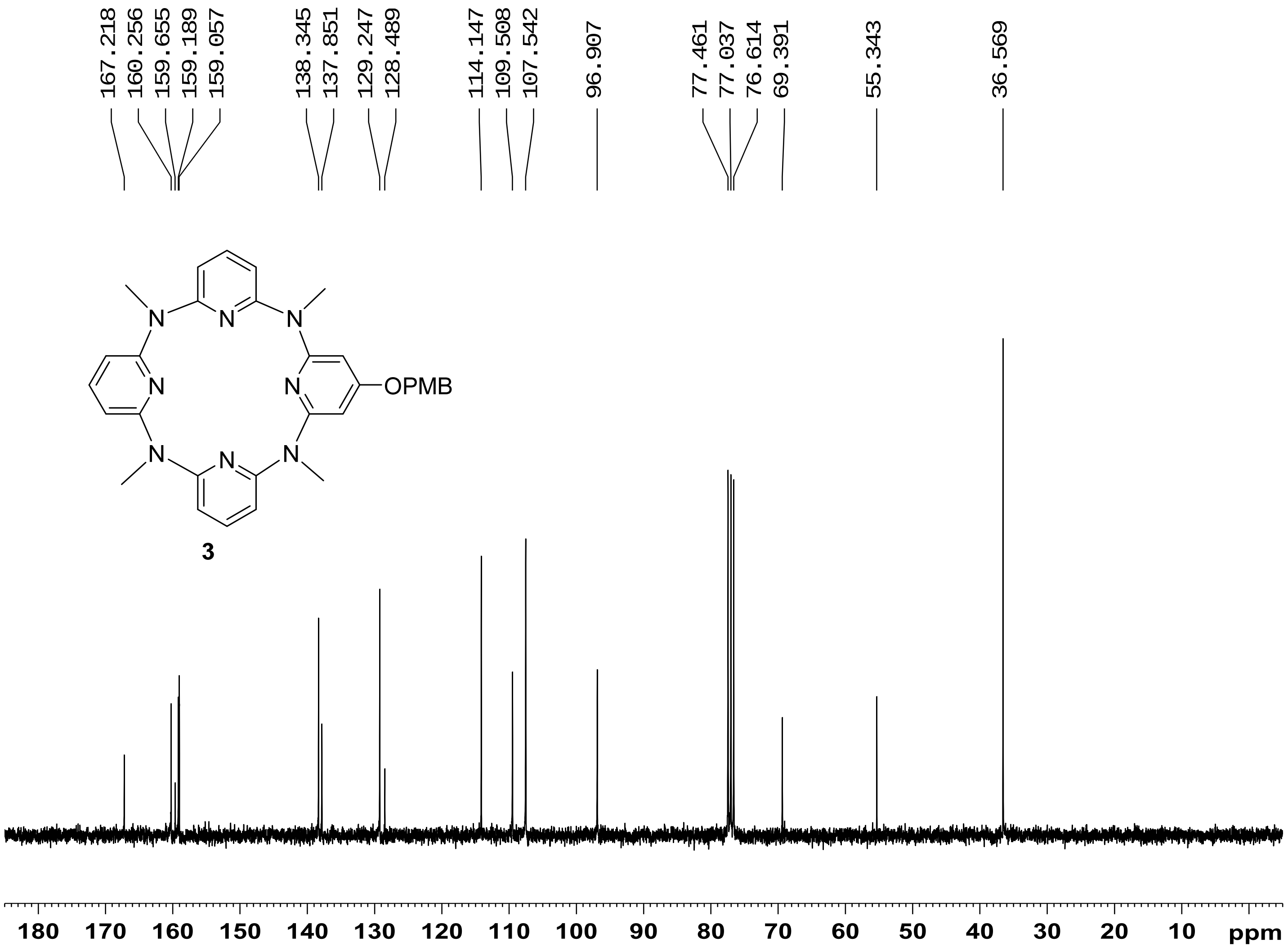


```
Current Data Parameters
NAME             zhex-201
EXPNO                  71
PROCNO                  1

F2 - Acquisition Parameters
Date_            20080821
Time                19.33
INSTRUM             spect
PROBHD   5 mm DUL 13C-1
PULPROG           zgpg30
TD                 65536
SOLVENT            CDCl3
NS                   178
DS                     4
SWH            17985.611 Hz
FIDRES          0.274439 Hz
AQ             1.8219508 sec
RG                 322.5
DW                27.800 usec
DE                  6.00 usec
TE                 301.5 K
D1            2.00000000 sec
d11           0.03000000 sec
DELTA         1.89999998 sec
MCREST        0.00000000 sec
MCWRK         0.01500000 sec

======== CHANNEL f1 ========
NUC1                 13C
P1                 12.50 usec
PL1                 2.00 dB
SFO1          75.4752953 MHz

======== CHANNEL f2 ========
CPDPRG2          waltz16
NUC2                  1H
PCPD2              80.00 usec
PL2                -1.00 dB
PL12               20.16 dB
PL13               16.98 dB
SFO2         300.1312005 MHz

F2 - Processing parameters
SI                 32768
SF            75.4677490 MHz
WDW                   EM
SSB                    0
LB                  1.00 Hz
GB                     0
PC                  1.40
```

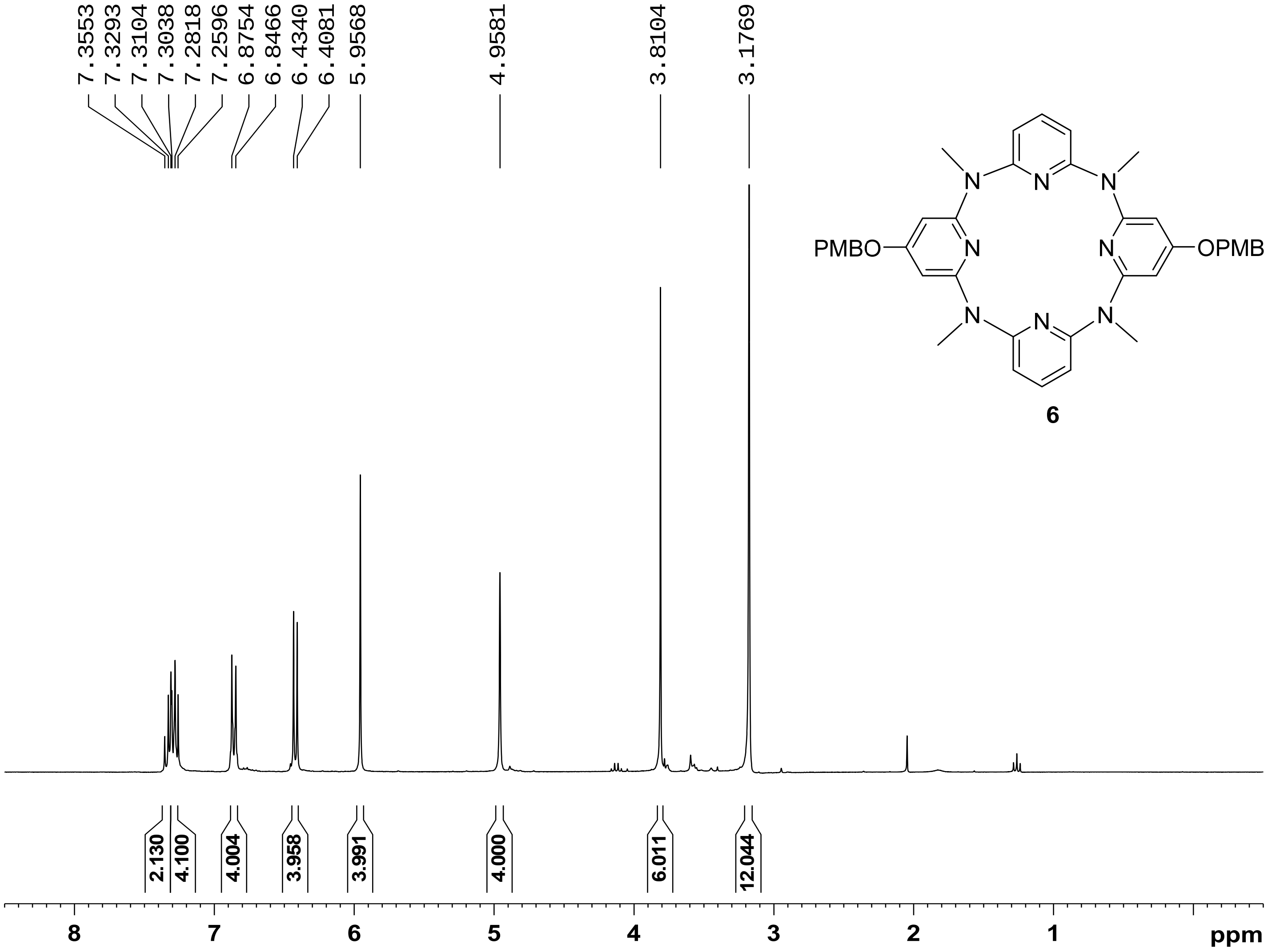

6

```
Current Data Parameters
NAME          zhex-183-3
EXPNO                 10
PROCNO                 1

F2 - Acquisition Parameters
Date_           20080512
Time               21.32
INSTRUM            spect
PROBHD   5 mm DUL 13C-1
PULPROG             zg30
TD                 65536
SOLVENT            CDCl3
NS                    16
DS                     0
SWH            8992.806 Hz
FIDRES         0.137219 Hz
AQ            3.6438515 sec
RG                 228.1
DW                55.600 usec
DE                  6.00 usec
TE                 300.0 K
D1            1.00000000 sec
MCREST        0.00000000 sec
MCWRK         0.01500000 sec

======== CHANNEL f1 ========
NUC1                  1H
P1                  7.00 usec
PL1                -1.00 dB
SFO1         300.1324010 MHz

F2 - Processing parameters
SI                 32768
SF           300.1300060 MHz
WDW                   EM
SSB                    0
LB                  0.30 Hz
GB                     0
PC                  1.00
```

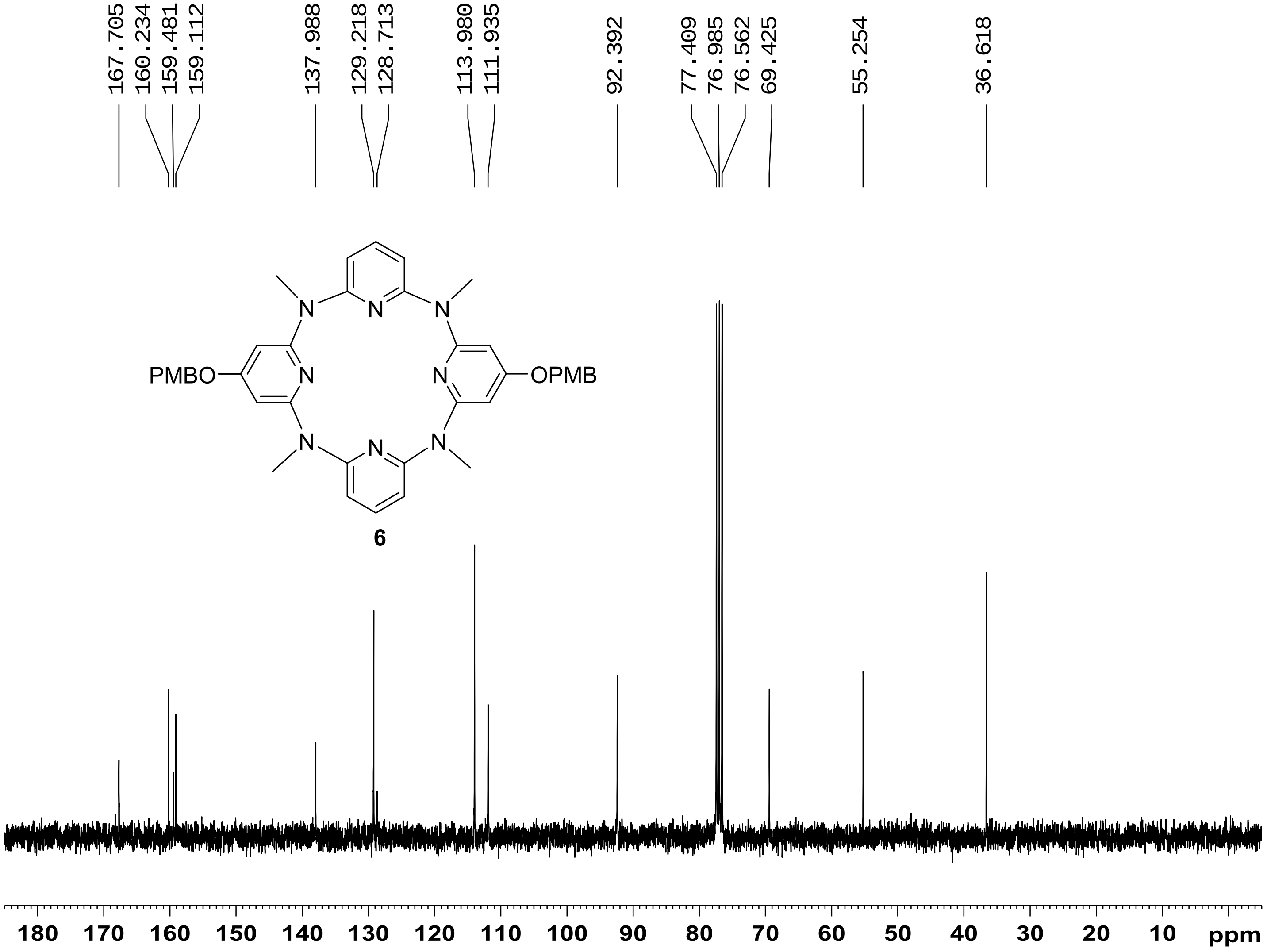

Current Data Parameters
NAME zhex-183-3
EXPNO 11
PROCNO 1

F2 - Acquisition Parameters
Date_ 20080512
Time 21.37
INSTRUM spect
PROBHD 5 mm DUL 13C-1
PULPROG zgpg30
TD 65536
SOLVENT CDCl3
NS 190
DS 4
SWH 17985.611 Hz
FIDRES 0.274439 Hz
AQ 1.8219508 sec
RG 812.7
DW 27.800 usec
DE 6.00 usec
TE 301.7 K
D1 2.00000000 sec
d11 0.03000000 sec
DELTA 1.89999998 sec
MCREST 0.00000000 sec
MCWRK 0.01500000 sec

======== CHANNEL f1 ========
NUC1 13C
P1 12.50 usec
PL1 2.00 dB
SFO1 75.4752953 MHz

======== CHANNEL f2 ========
CPDPRG2 waltz16
NUC2 1H
PCPD2 80.00 usec
PL2 -1.00 dB
PL12 20.16 dB
PL13 16.98 dB
SFO2 300.1312005 MHz

F2 - Processing parameters
SI 32768
SF 75.4677490 MHz
WDW EM
SSB 0
LB 1.00 Hz
GB 0
PC 1.40

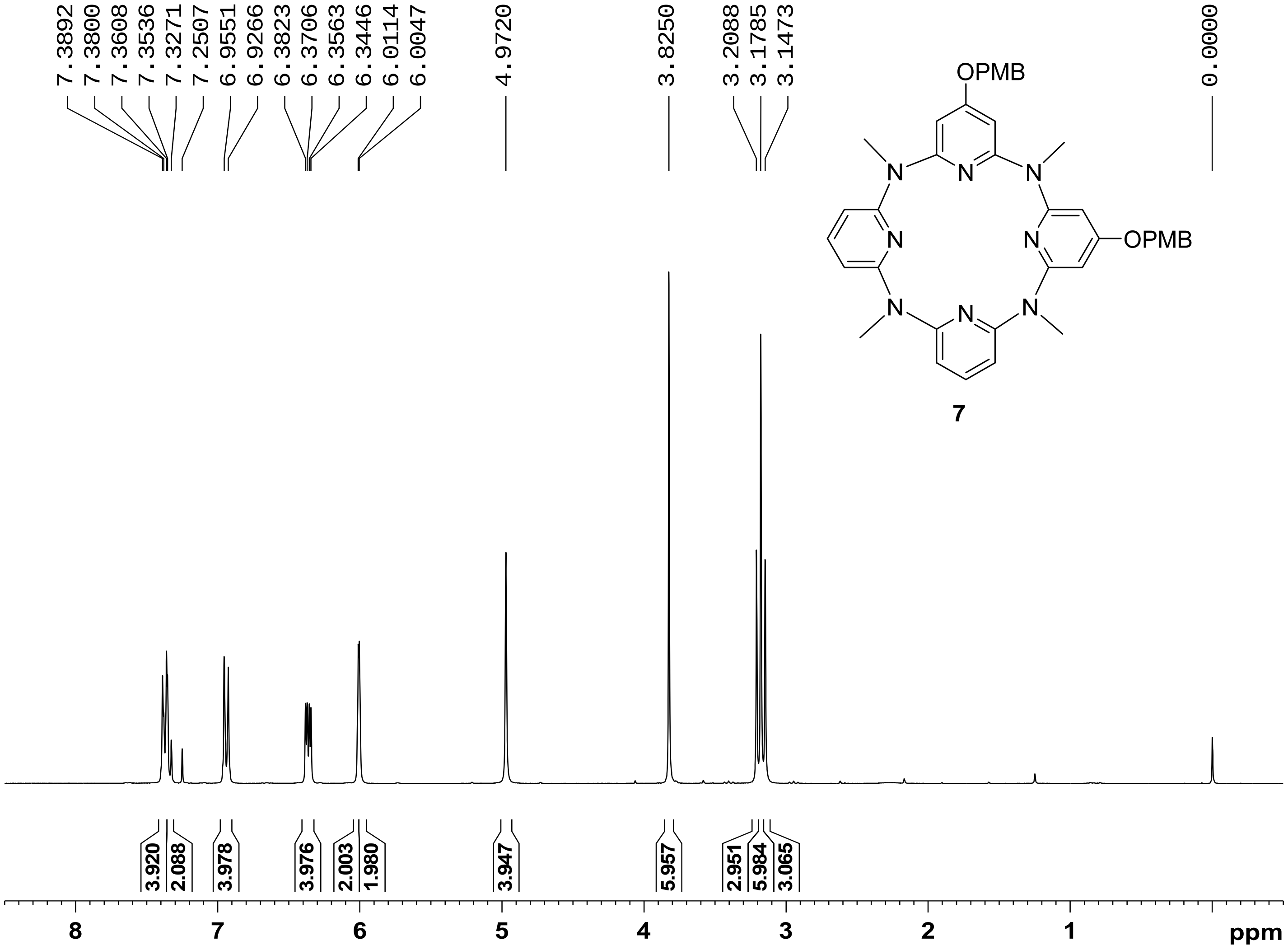

7

```
Current Data Parameters
NAME            zhex-220
EXPNO                 10
PROCNO                 1

F2 - Acquisition Parameters
Date_           20100305
Time               11.18
INSTRUM            spect
PROBHD   5 mm DUL 13C-1
PULPROG             zg30
TD                 32768
SOLVENT            CDCl3
NS                    16
DS                     0
SWH           8992.806 Hz
FIDRES        0.274439 Hz
AQ          1.8219508 sec
RG                   181
DW            55.600 usec
DE              8.00 usec
TE               296.0 K
D1         2.00000000 sec
TD0                    1

======== CHANNEL f1 ========
NUC1                  1H
P1             10.80 usec
PL1              3.00 dB
SFO1      300.1318008 MHz

F2 - Processing parameters
SI                 32768
SF        300.1300090 MHz
WDW                   EM
SSB                    0
LB               0.30 Hz
GB                     0
PC                  1.00
```

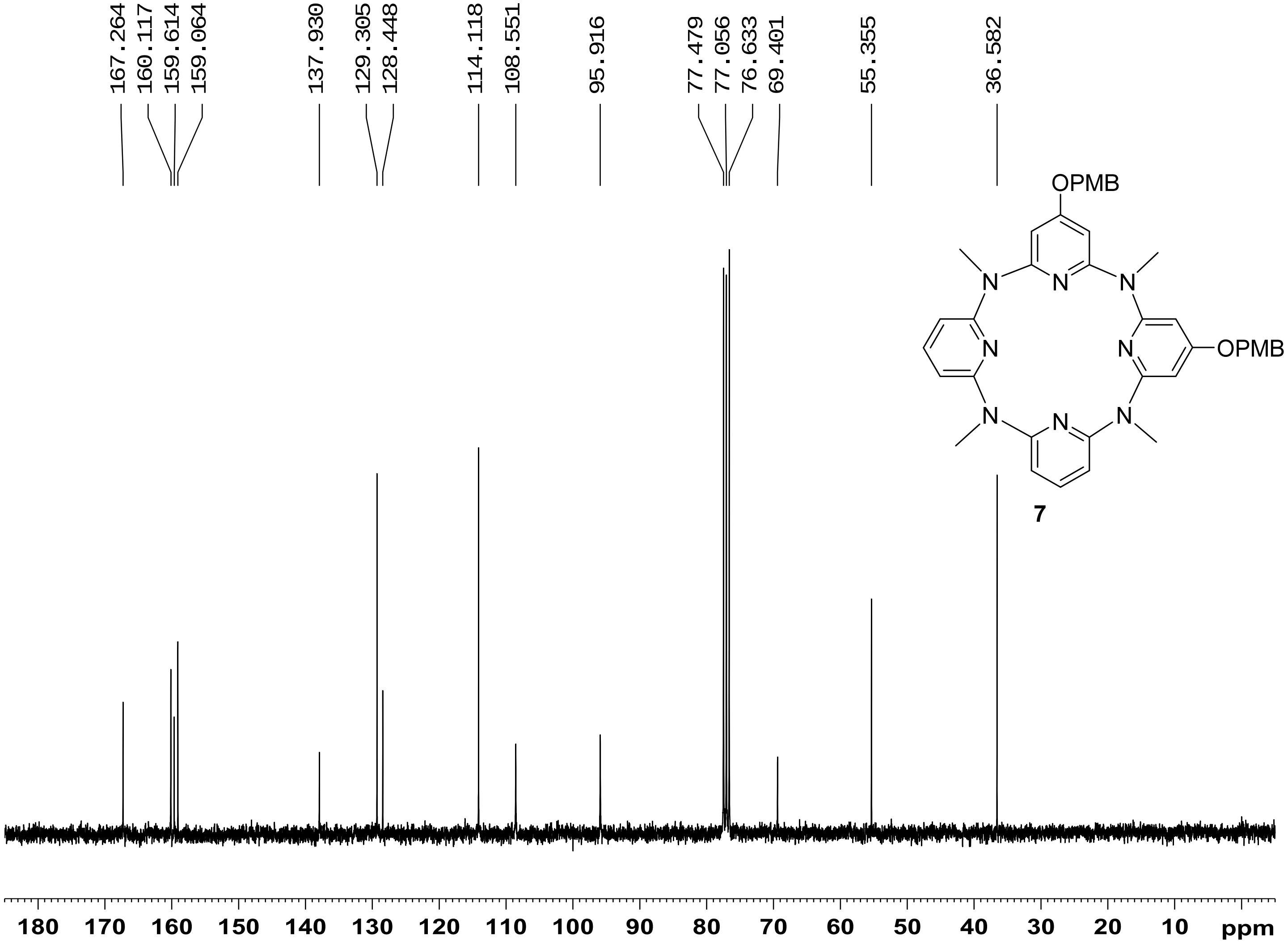

7

```
Current Data Parameters
NAME             zhex-220
EXPNO                  11
PROCNO                  1

F2 - Acquisition Parameters
Date_            20100305
Time                11.21
INSTRUM             spect
PROBHD   5 mm DUL 13C-1
PULPROG            zgig30
TD                  32768
SOLVENT             CDCl3
NS                    276
DS                      0
SWH           18832.393 Hz
FIDRES         0.574719 Hz
AQ            0.8700404 sec
RG               9195.2
DW               26.550 usec
DE                 8.00 usec
TE                296.2 K
D1           2.00000000 sec
D11          0.03000000 sec
TD0                     1

======== CHANNEL f1 ========
NUC1                  13C
P1                  12.50 usec
PL1                  2.00 dB
SFO1          75.4752953 MHz

======== CHANNEL f2 ========
CPDPRG2           waltz16
NUC2                   1H
PCPD2              100.00 usec
PL2                  3.00 dB
PL12                22.33 dB
SFO2         300.1312005 MHz

F2 - Processing parameters
SI                  32768
SF            75.4677490 MHz
WDW                    EM
SSB                     0
LB                   1.00 Hz
GB                      0
PC                   1.40
```

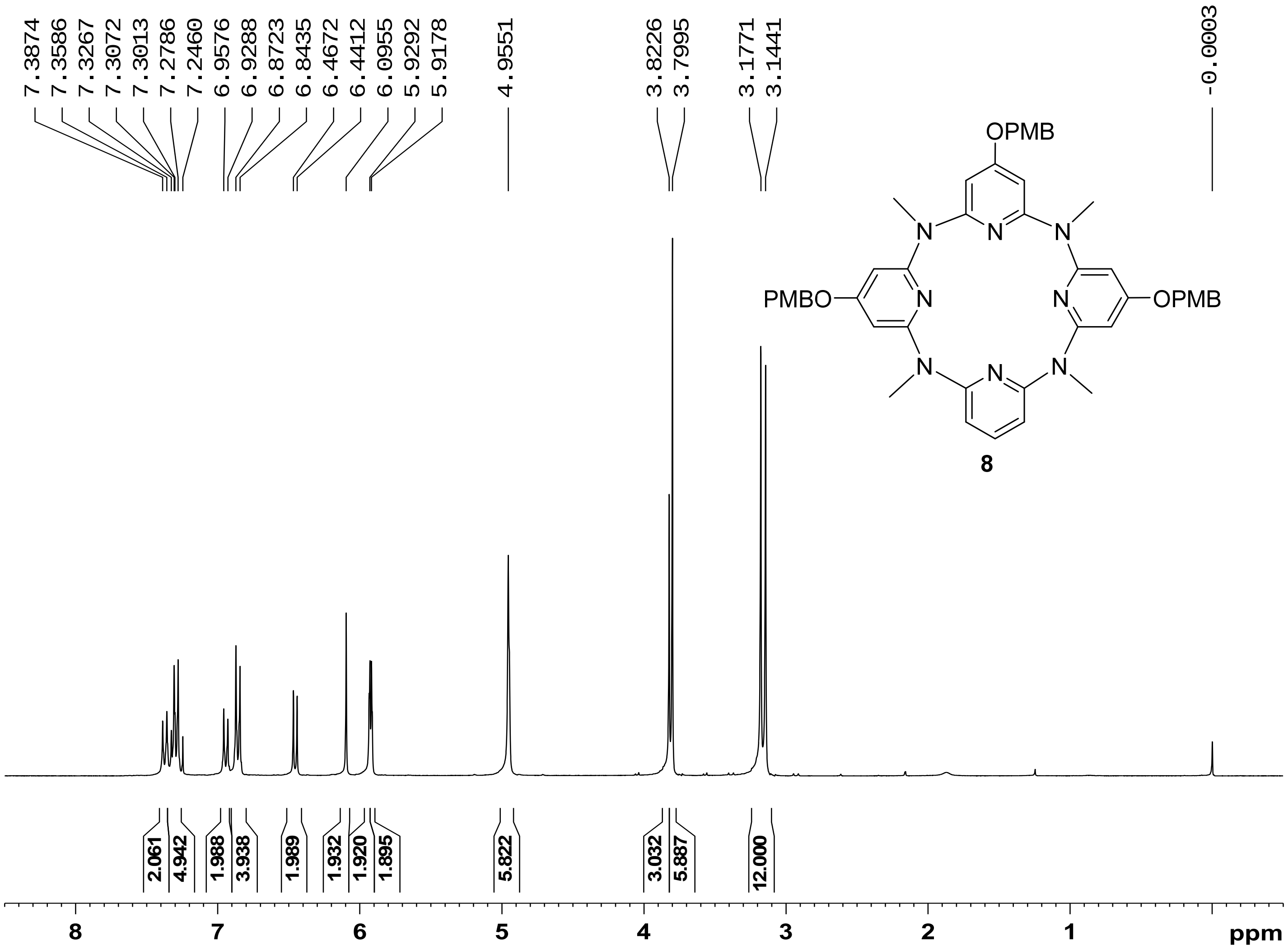


```
Current Data Parameters
NAME             zhex-202
EXPNO                  20
PROCNO                  1

F2 - Acquisition Parameters
Date_            20090211
Time                20.13
INSTRUM             spect
PROBHD   5 mm DUL 13C-1
PULPROG              zg30
TD                  65536
SOLVENT             CDCl3
NS                     16
DS                      0
SWH             8992.806 Hz
FIDRES          0.137219 Hz
AQ             3.6438515 sec
RG                    181
DW                 55.600 usec
DE                   6.00 usec
TE                  673.2 K
D1             1.00000000 sec
MCREST         0.00000000 sec
MCWRK          0.01500000 sec

======== CHANNEL f1 ========
NUC1                   1H
P1                   7.00 usec
PL1                 -1.00 dB
SFO1          300.1324010 MHz

F2 - Processing parameters
SI                  32768
SF            300.1300097 MHz
WDW                    EM
SSB                     0
LB                   0.30 Hz
GB                      0
PC                   1.00
```

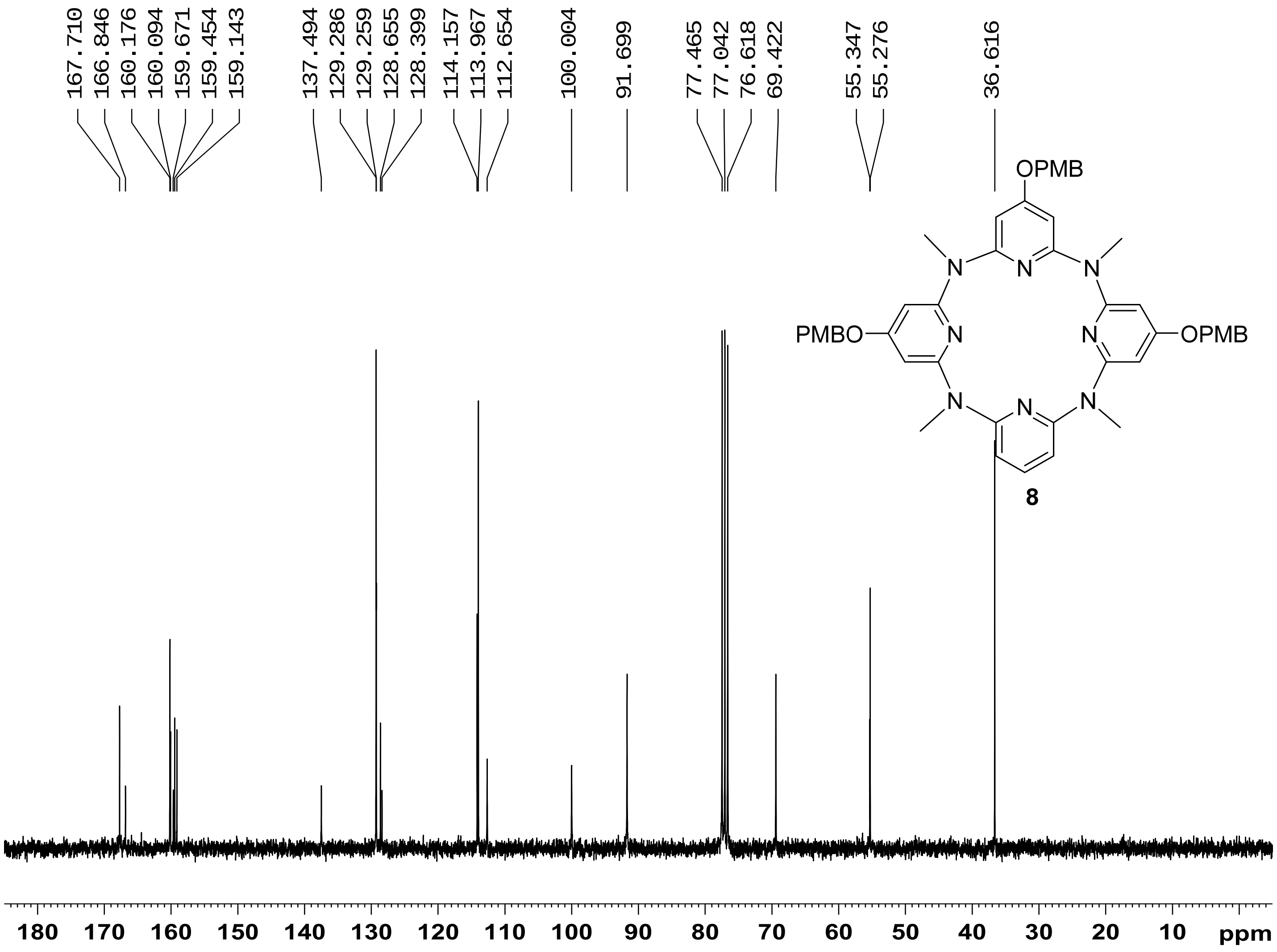


```
Current Data Parameters
NAME            zhex-202
EXPNO                 21
PROCNO                 1

F2 - Acquisition Parameters
Date_           20090211
Time               20.21
INSTRUM            spect
PROBHD   5 mm DUL 13C-1
PULPROG          zgpg30
TD                 65536
SOLVENT            CDCl3
NS                   186
DS                     4
SWH            17985.611 Hz
FIDRES          0.274439 Hz
AQ             1.8219508 sec
RG                1448.2
DW                27.800 usec
DE                  6.00 usec
TE                 673.2 K
D1            2.00000000 sec
d11           0.03000000 sec
DELTA         1.89999998 sec
MCREST        0.00000000 sec
MCWRK         0.01500000 sec

======== CHANNEL f1 ========
NUC1                 13C
P1                 12.50 usec
PL1                 2.00 dB
SFO1          75.4752953 MHz

======== CHANNEL f2 ========
CPDPRG2          waltz16
NUC2                  1H
PCPD2              80.00 usec
PL2                -1.00 dB
PL12               20.16 dB
PL13               16.98 dB
SFO2         300.1312005 MHz

F2 - Processing parameters
SI                 32768
SF            75.4677490 MHz
WDW                   EM
SSB                    0
LB                  1.00 Hz
GB                     0
PC                  1.40
```

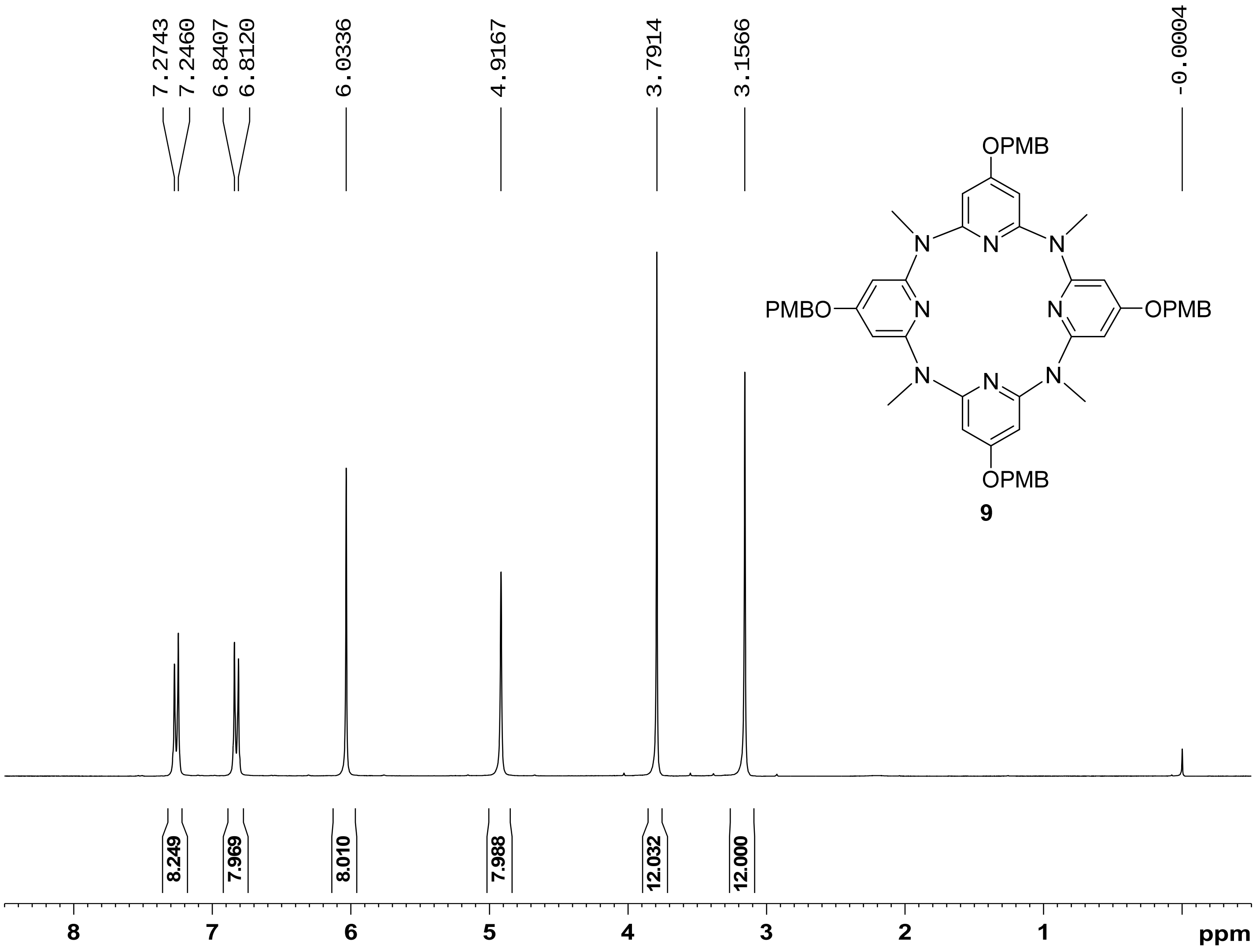


```
Current Data Parameters
NAME             zhex-218
EXPNO                  10
PROCNO                  1

F2 - Acquisition Parameters
Date_            20100307
Time                14.44
INSTRUM             spect
PROBHD   5 mm DUL 13C-1
PULPROG              zg30
TD                  65536
SOLVENT             CDCl3
NS                     16
DS                      0
SWH              8992.806 Hz
FIDRES           0.137219 Hz
AQ              3.6438515 sec
RG                    128
DW                 55.600 usec
DE                   8.00 usec
TE                  297.4 K
D1             1.00000000 sec
TD0                     1

======== CHANNEL f1 ========
NUC1                   1H
P1                  10.80 usec
PL1                  3.00 dB
SFO1          300.1324010 MHz

F2 - Processing parameters
SI                  32768
SF            300.1300098 MHz
WDW                    EM
SSB                     0
LB                   0.30 Hz
GB                      0
PC                   1.00
```

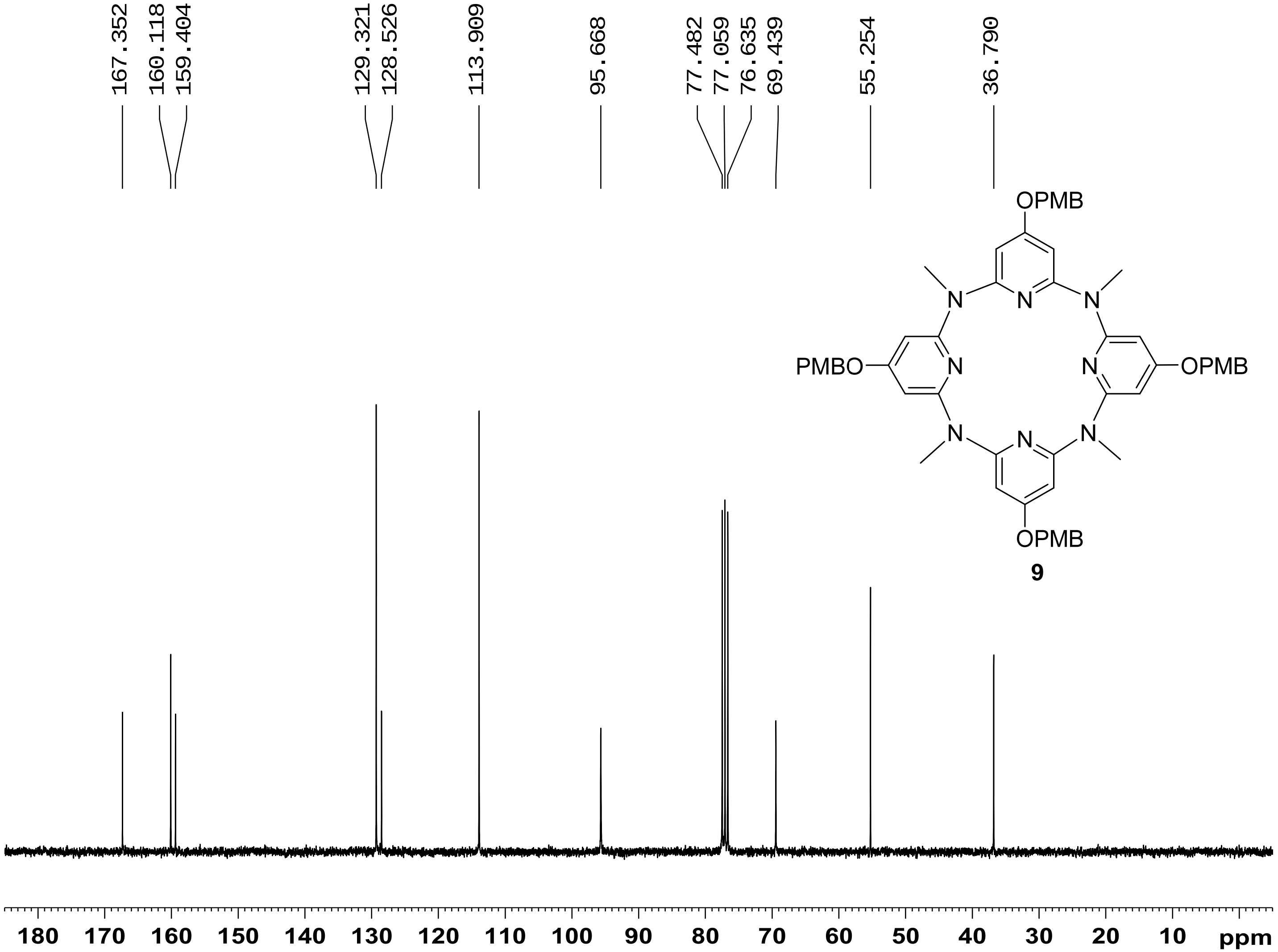


```
Current Data Parameters
NAME             zhex-218
EXPNO                  11
PROCNO                  1

F2 - Acquisition Parameters
Date_            20100307
Time                14.48
INSTRUM             spect
PROBHD   5 mm DUL 13C-1
PULPROG           zgpg30
TD                 65536
SOLVENT            CDCl3
NS                   217
DS                     4
SWH          17985.611 Hz
FIDRES        0.274439 Hz
AQ           1.8219508 sec
RG                  8192
DW              27.800 usec
DE                8.00 usec
TE               297.7 K
D1          2.00000000 sec
D11         0.03000000 sec
TD0                    1

======== CHANNEL f1 ========
NUC1                 13C
P1                 12.50 usec
PL1                 2.00 dB
SFO1          75.4752953 MHz

======== CHANNEL f2 ========
CPDPRG2          waltz16
NUC2                  1H
PCPD2             100.00 usec
PL2                 3.00 dB
PL12               22.33 dB
PL13               23.00 dB
SFO2         300.1312005 MHz

F2 - Processing parameters
SI                 32768
SF            75.4677490 MHz
WDW                   EM
SSB                    0
LB                  1.00 Hz
GB                     0
PC                  1.40
```

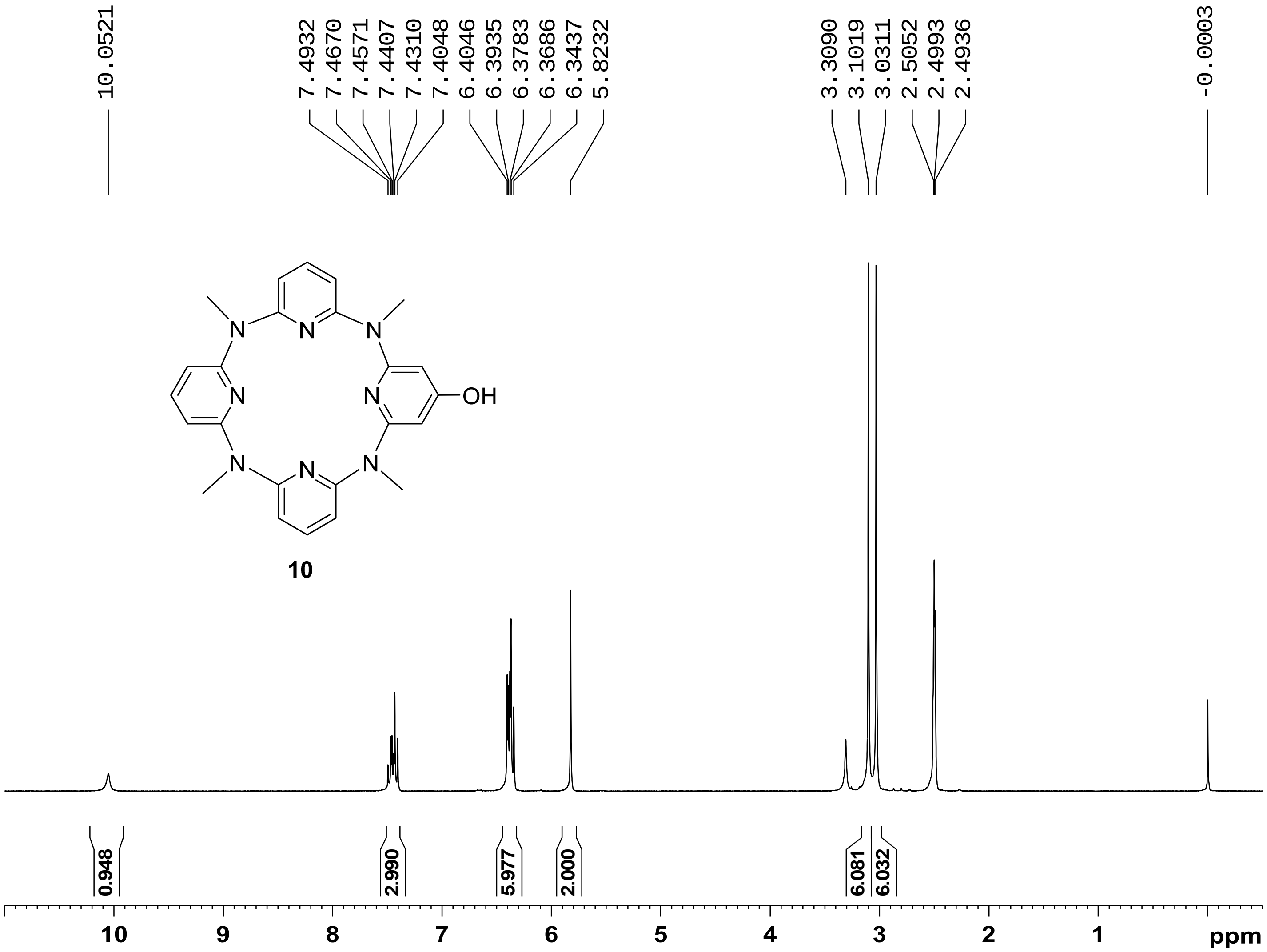


```
Current Data Parameters
NAME            zhex-216
EXPNO                 10
PROCNO                 1

F2 - Acquisition Parameters
Date_           20081010
Time               16.39
INSTRUM            spect
PROBHD   5 mm DUL 13C-1
PULPROG             zg30
TD                 65536
SOLVENT             DMSO
NS                    16
DS                     0
SWH            8992.806 Hz
FIDRES         0.137219 Hz
AQ            3.6438515 sec
RG                   362
DW                55.600 usec
DE                  6.00 usec
TE                 300.4 K
D1            1.00000000 sec
MCREST        0.00000000 sec
MCWRK         0.01500000 sec

======== CHANNEL f1 ========
NUC1                  1H
P1                  7.00 usec
PL1                -1.00 dB
SFO1         300.1324010 MHz

F2 - Processing parameters
SI                 32768
SF           300.1300010 MHz
WDW                   EM
SSB                    0
LB                  0.30 Hz
GB                     0
PC                  1.00
```

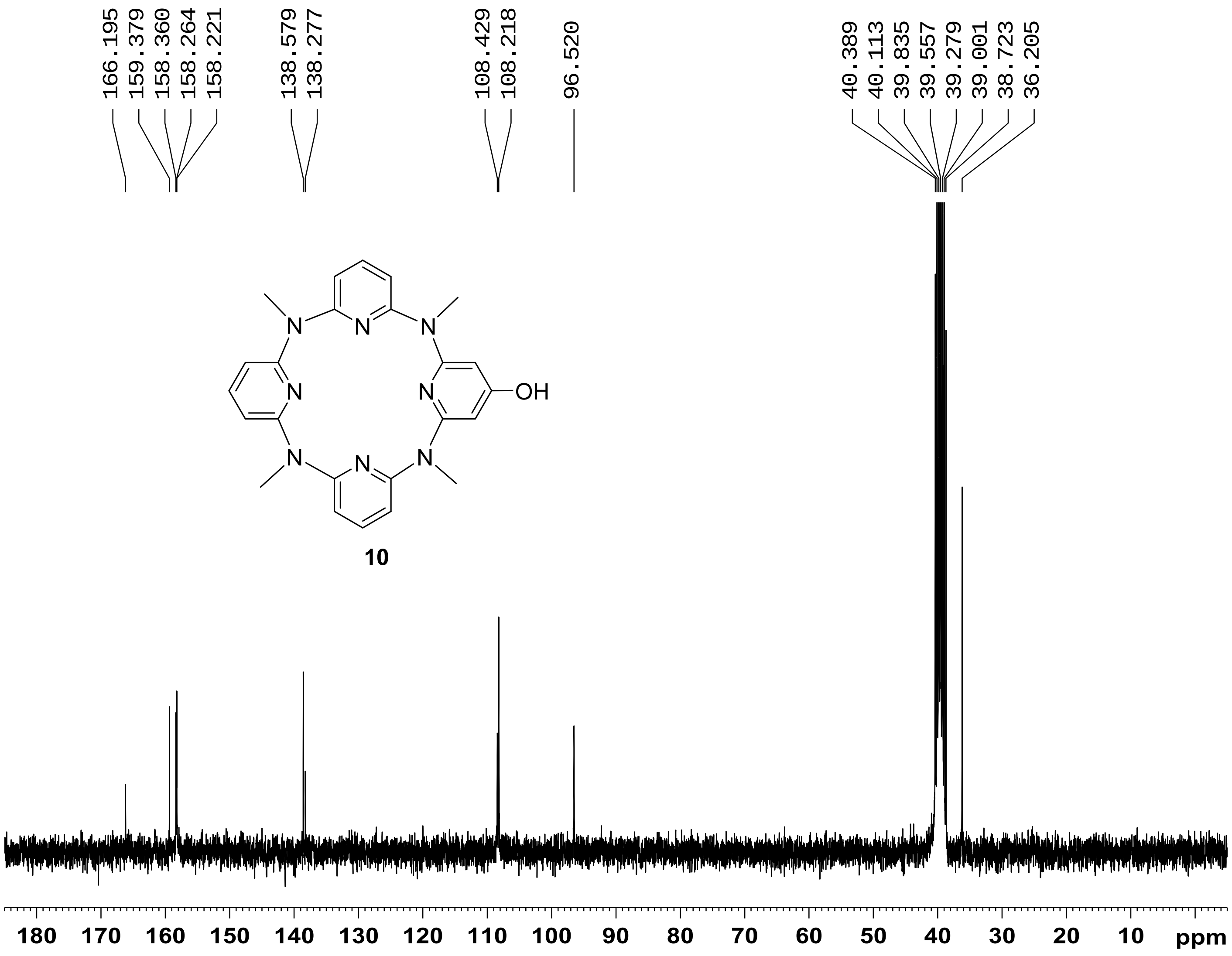


```
Current Data Parameters
NAME             zhex-216
EXPNO                  20
PROCNO                  1

F2 - Acquisition Parameters
Date_            20081010
Time                17.06
INSTRUM             spect
PROBHD   5 mm DUL 13C-1
PULPROG           zgpg30
TD                 65536
SOLVENT             DMSO
NS                  1024
DS                     4
SWH           17985.611 Hz
FIDRES         0.274439 Hz
AQ            1.8219508 sec
RG                4597.6
DW                27.800 usec
DE                  6.00 usec
TE                 301.8 K
D1            2.00000000 sec
d11           0.03000000 sec
DELTA         1.89999998 sec
MCREST        0.00000000 sec
MCWRK         0.01500000 sec

======== CHANNEL f1 ========
NUC1                 13C
P1                 12.50 usec
PL1                 2.00 dB
SFO1         75.4752953 MHz

======== CHANNEL f2 ========
CPDPRG2          waltz16
NUC2                  1H
PCPD2              80.00 usec
PL2                -1.00 dB
PL12               20.16 dB
PL13               16.98 dB
SFO2        300.1312005 MHz

F2 - Processing parameters
SI                 32768
SF            75.4677867 MHz
WDW                   EM
SSB                    0
LB                  1.00 Hz
GB                     0
PC                  1.40
```

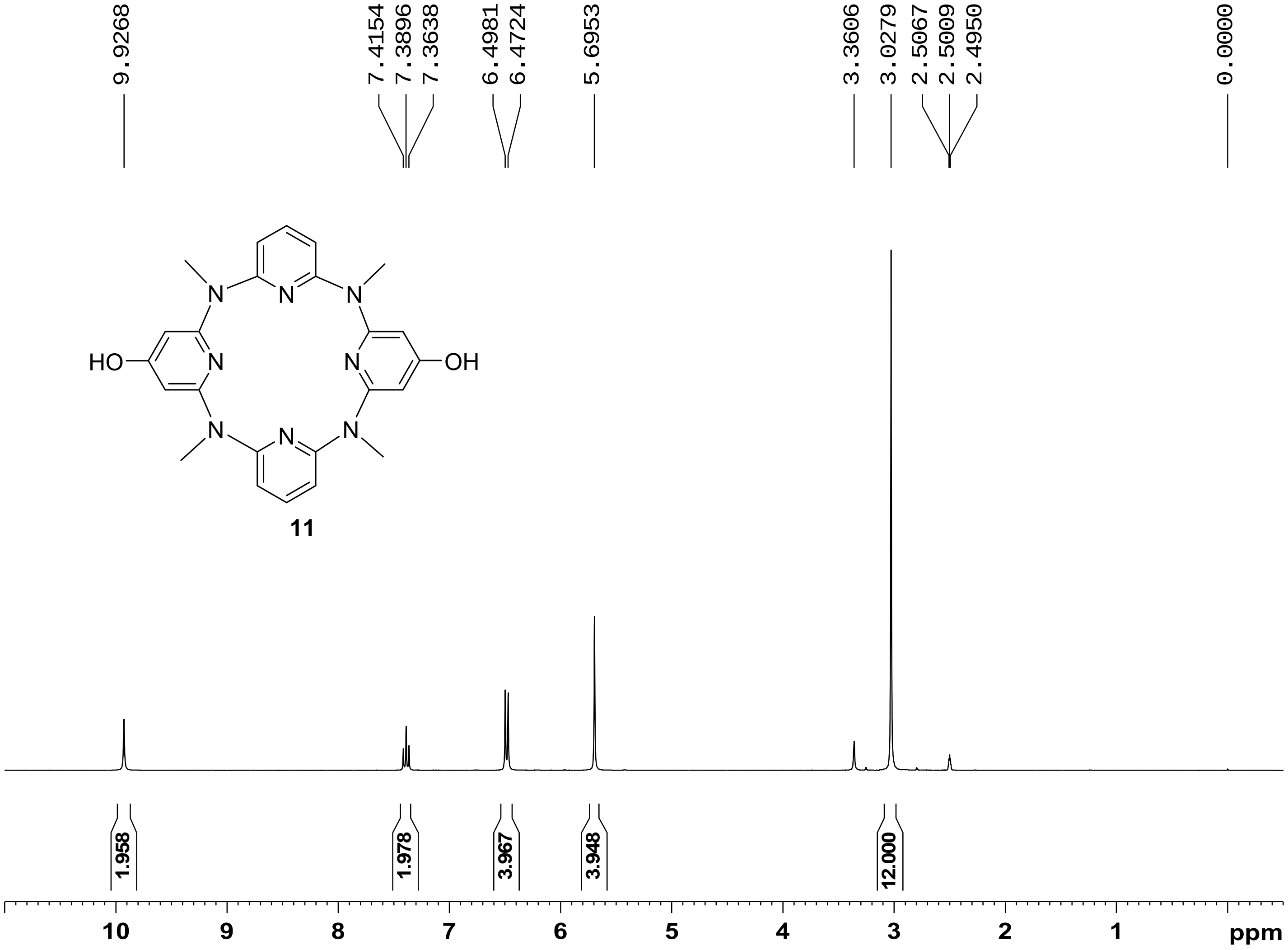

11

```
Current Data Parameters
NAME            zhex-189
EXPNO                 60
PROCNO                 1

F2 - Acquisition Parameters
Date_           20100307
Time               21.22
INSTRUM            spect
PROBHD   5 mm DUL 13C-1
PULPROG             zg30
TD                 32768
SOLVENT             DMSO
NS                    16
DS                     0
SWH             8992.806 Hz
FIDRES          0.274439 Hz
AQ             1.8219508 sec
RG                   181
DW                55.600 usec
DE                  8.00 usec
TE                 298.1 K
D1            2.00000000 sec
TD0                    1

======== CHANNEL f1 ========
NUC1                  1H
P1                 10.80 usec
PL1                 3.00 dB
SFO1         300.1318008 MHz

F2 - Processing parameters
SI                 32768
SF           300.1300005 MHz
WDW                   EM
SSB                    0
LB                  0.30 Hz
GB                     0
PC                  1.00
```

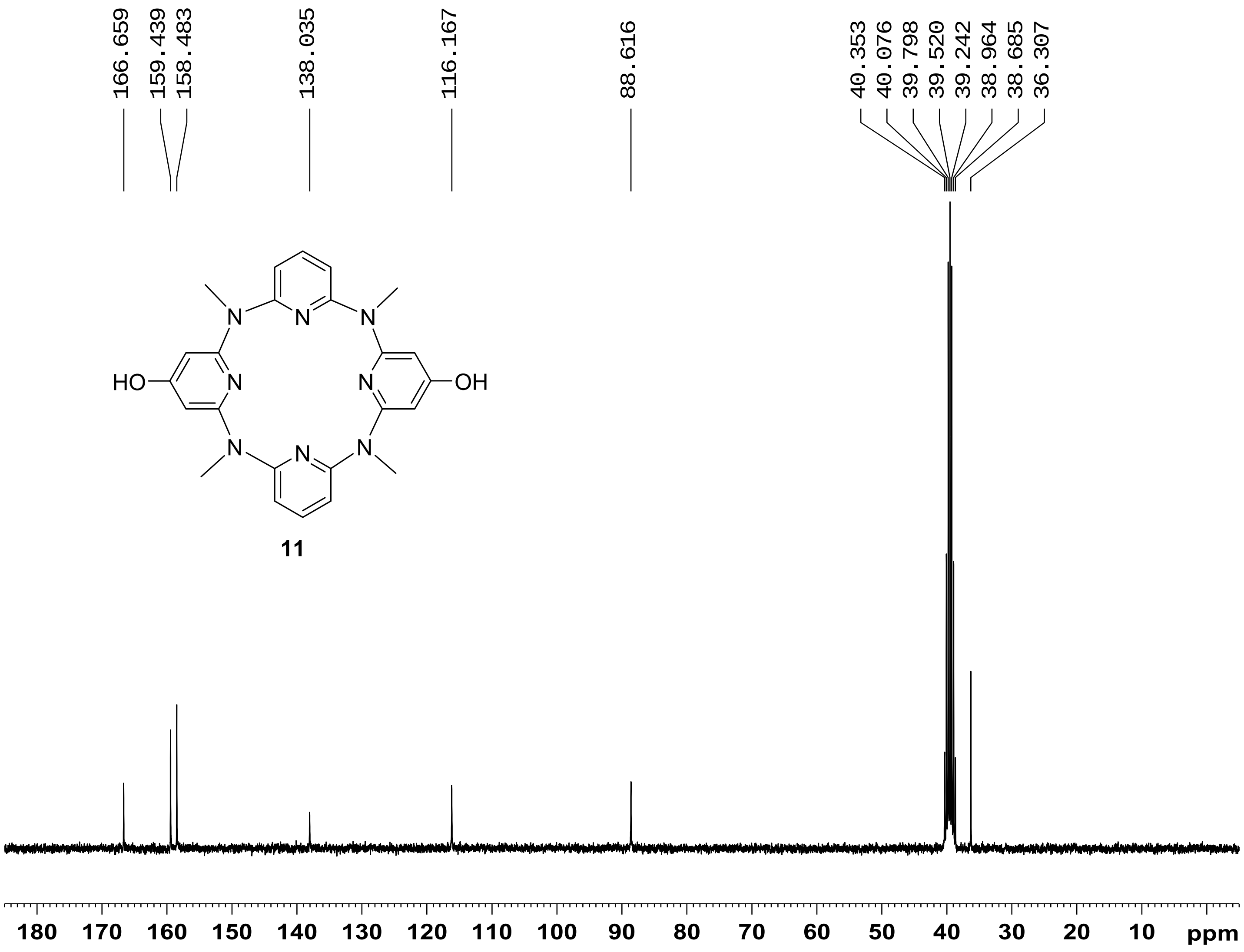

11

```
Current Data Parameters
NAME              zhex-189
EXPNO                   61
PROCNO                   1

F2 - Acquisition Parameters
Date_             20100307
Time                 21.29
INSTRUM              spect
PROBHD   5 mm DUL 13C-1
PULPROG            zgig30
TD                  32768
SOLVENT              DMSO
NS                    354
DS                      0
SWH           18832.393 Hz
FIDRES         0.574719 Hz
AQ            0.8700404 sec
RG                  16384
DW               26.550 usec
DE                 8.00 usec
TE                298.2 K
D1           2.00000000 sec
D11          0.03000000 sec
TD0                     1

======== CHANNEL f1 ========
NUC1                  13C
P1                12.50 usec
PL1                2.00 dB
SFO1        75.4752953 MHz

======== CHANNEL f2 ========
CPDPRG2           waltz16
NUC2                   1H
PCPD2            100.00 usec
PL2                3.00 dB
PL12              22.33 dB
SFO2       300.1312005 MHz

F2 - Processing parameters
SI                  32768
SF          75.4677835 MHz
WDW                    EM
SSB                     0
LB                 1.00 Hz
GB                      0
PC                   1.40
```

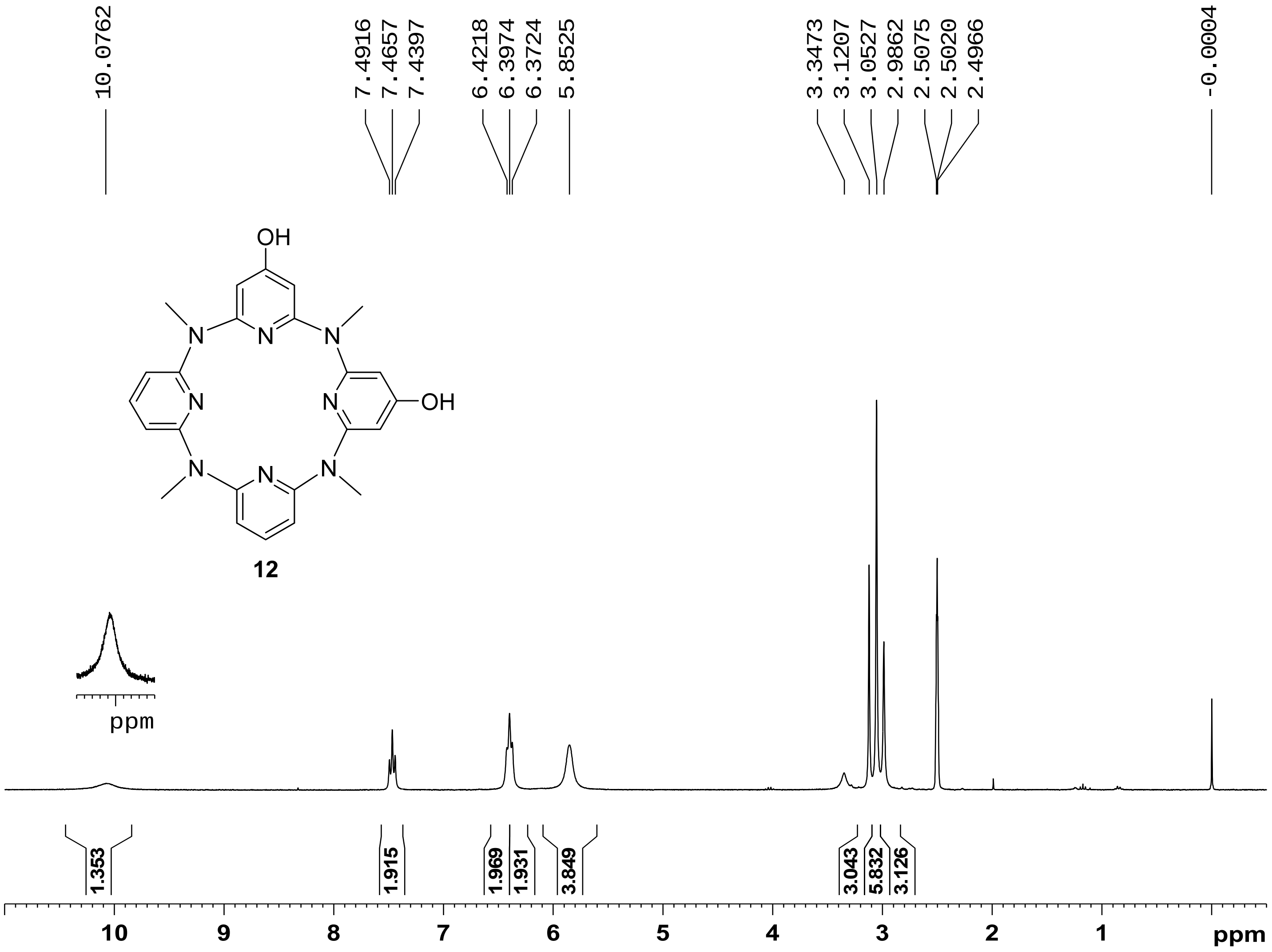


```
Current Data Parameters
NAME            zhex-251
EXPNO                 10
PROCNO                 1

F2 - Acquisition Parameters
Date_           20100302
Time               20.24
INSTRUM            spect
PROBHD   5 mm DUL 13C-1
PULPROG             zg30
TD                 65536
SOLVENT             DMSO
NS                    16
DS                     0
SWH             8992.806 Hz
FIDRES          0.137219 Hz
AQ             3.6438515 sec
RG                   512
DW                55.600 usec
DE                  8.00 usec
TE                 293.7 K
D1            1.00000000 sec
TD0                    1

======== CHANNEL f1 ========
NUC1                  1H
P1                 10.80 usec
PL1                 3.00 dB
SFO1        300.1324010 MHz

F2 - Processing parameters
SI                 32768
SF          300.1300000 MHz
WDW                   EM
SSB                    0
LB                  0.30 Hz
GB                     0
PC                  1.00
```

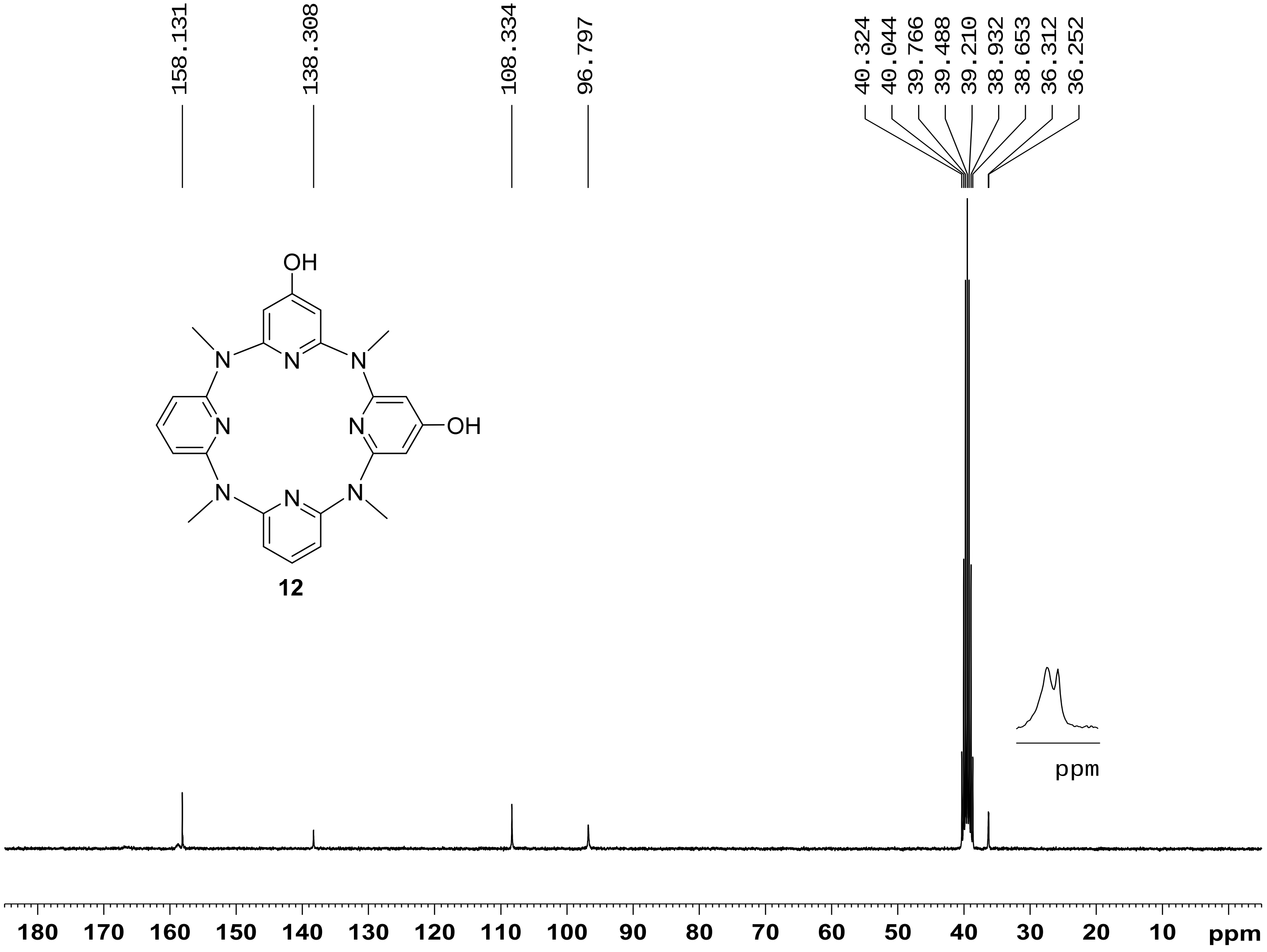


```
Current Data Parameters
NAME            zhex-251
EXPNO                 22
PROCNO                 1

F2 - Acquisition Parameters
Date_           20100307
Time               17.52
INSTRUM            spect
PROBHD   5 mm DUL 13C-1
PULPROG           zgpg30
TD                 65536
SOLVENT             DMSO
NS                  2989
DS                     4
SWH            17985.611 Hz
FIDRES          0.274439 Hz
AQ             1.8219508 sec
RG                 16384
DW                27.800 usec
DE                  8.00 usec
TE                 297.5 K
D1            2.00000000 sec
D11           0.03000000 sec
TD0                    1

======== CHANNEL f1 ========
NUC1                 13C
P1                 12.50 usec
PL1                 2.00 dB
SFO1          75.4752953 MHz

======== CHANNEL f2 ========
CPDPRG2          waltz16
NUC2                  1H
PCPD2             100.00 usec
PL2                 3.00 dB
PL12               22.33 dB
PL13               23.00 dB
SFO2         300.1312005 MHz

F2 - Processing parameters
SI                 32768
SF            75.4677867 MHz
WDW                   EM
SSB                    0
LB                  1.00 Hz
GB                     0
PC                  1.40
```

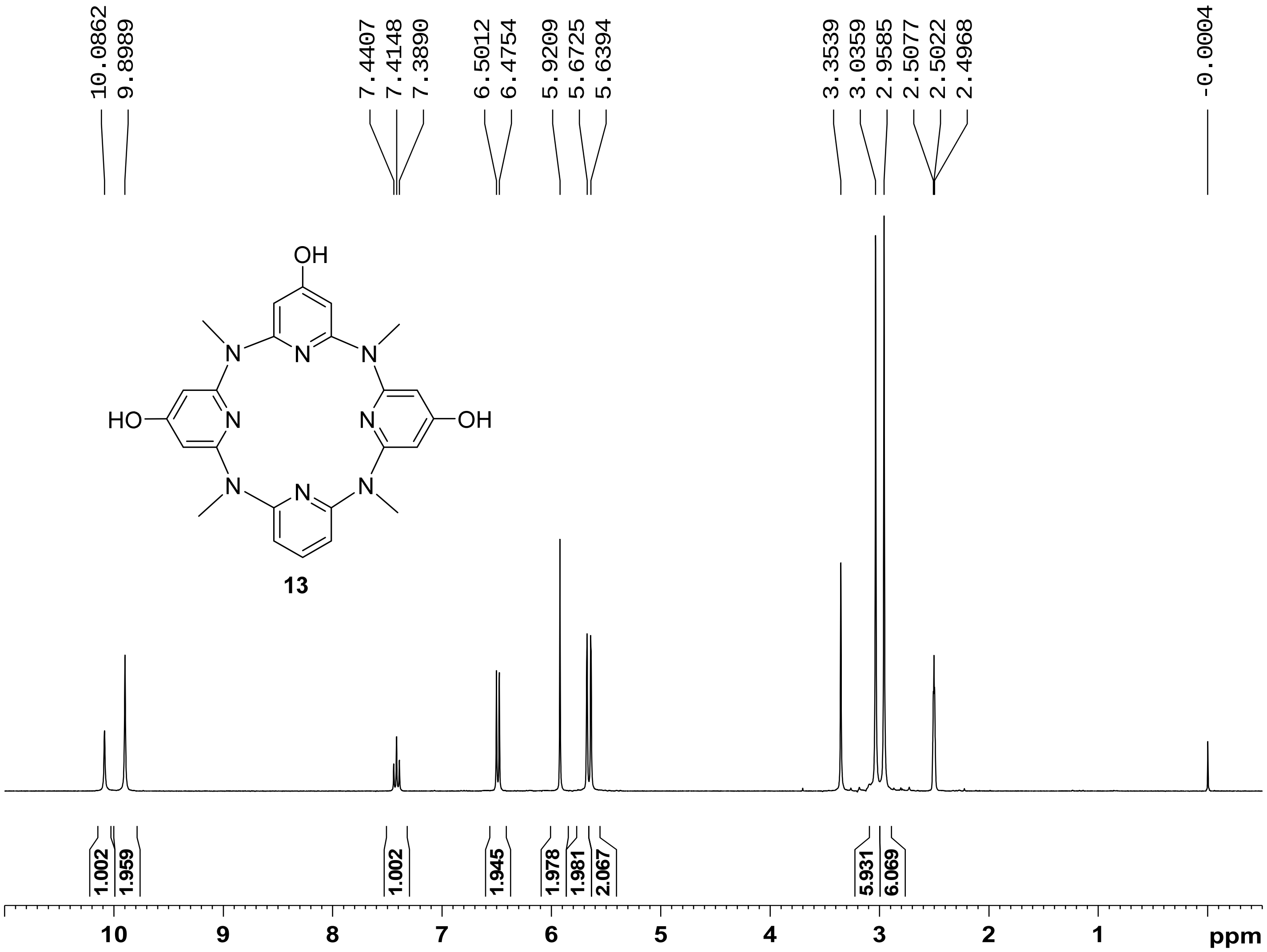


```
Current Data Parameters
NAME             zhex-257
EXPNO                  10
PROCNO                  1

F2 - Acquisition Parameters
Date_            20100302
Time                20.33
INSTRUM             spect
PROBHD   5 mm DUL 13C-1
PULPROG              zg30
TD                  65536
SOLVENT              DMSO
NS                     16
DS                      0
SWH              8992.806 Hz
FIDRES           0.137219 Hz
AQ              3.6438515 sec
RG                    362
DW                 55.600 usec
DE                   8.00 usec
TE                  294.3 K
D1             1.00000000 sec
TD0                     1

======== CHANNEL f1 ========
NUC1                   1H
P1                  10.80 usec
PL1                  3.00 dB
SFO1          300.1324010 MHz

F2 - Processing parameters
SI                  32768
SF            300.1300000 MHz
WDW                    EM
SSB                     0
LB                   0.30 Hz
GB                      0
PC                   1.00
```

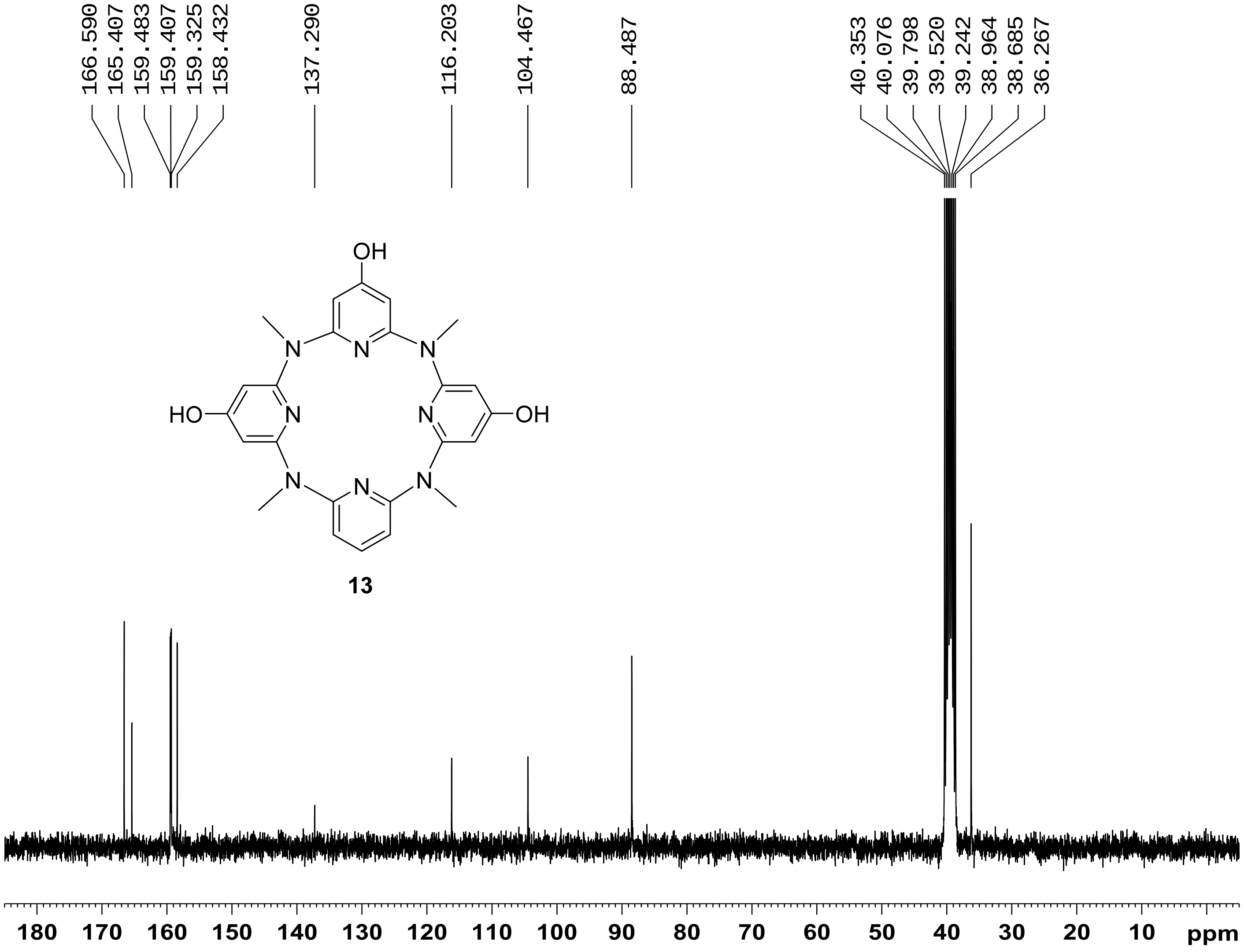

13

```
Current Data Parameters
NAME             zhex-257
EXPNO                  21
PROCNO                  1

F2 - Acquisition Parameters
Date_            20100304
Time                11.04
INSTRUM             spect
PROBHD   5 mm DUL 13C-1
PULPROG            zgig30
TD                  32768
SOLVENT              DMSO
NS                   2281
DS                      0
SWH            18832.393 Hz
FIDRES          0.574719 Hz
AQ             0.8700404 sec
RG                18390.4
DW                 26.550 usec
DE                   8.00 usec
TE                  293.7 K
D1             2.00000000 sec
D11            0.03000000 sec
TD0                     1

======== CHANNEL f1 ========
NUC1                  13C
P1                  12.50 usec
PL1                  2.00 dB
SFO1          75.4752953 MHz

======== CHANNEL f2 ========
CPDPRG2           waltz16
NUC2                   1H
PCPD2              100.00 usec
PL2                  3.00 dB
PL12                22.33 dB
SFO2         300.1312005 MHz

F2 - Processing parameters
SI                  32768
SF            75.4677814 MHz
WDW                    EM
SSB                     0
LB                   1.00 Hz
GB                      0
PC                   1.40
```

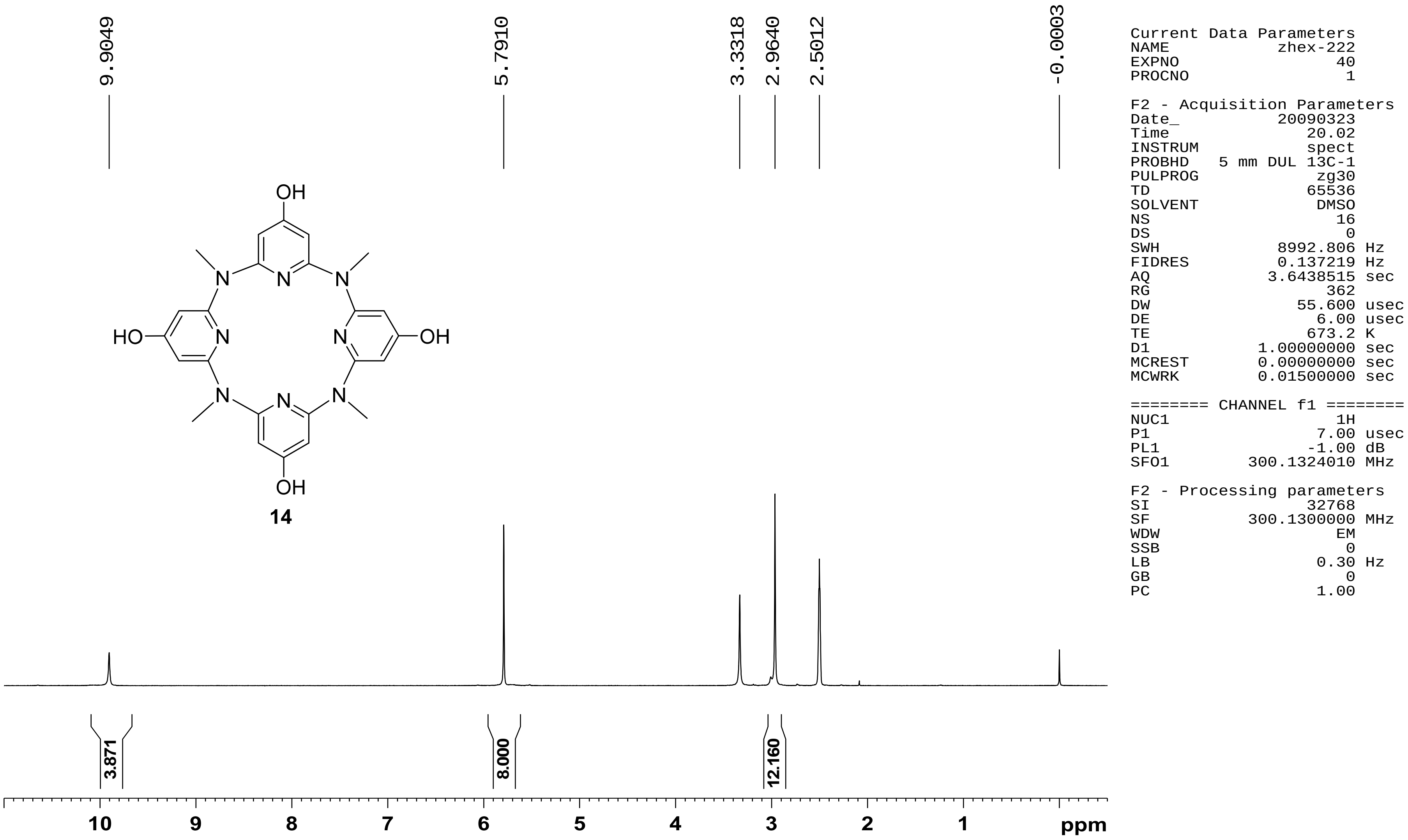
9.9049
5.7910
3.3318
2.9640
2.5012
-0.0003
OH
N
N
N
HO
N
N
OH
N
N
N
OH
14
3.871
8.000
12.160
10 9 8 7 6 5 4 3 2 1 ppm
Current Data Parameters
NAME zhex-222
EXPNO 40
PROCNO 1
F2 - Acquisition Parameters
Date_ 20090323
Time 20.02
INSTRUM spect
PROBHD 5 mm DUL 13C-1
PULPROG zg30
TD 65536
SOLVENT DMSO
NS 16
DS 0
SWH 8992.806 Hz
FIDRES 0.137219 Hz
AQ 3.6438515 sec
RG 362
DW 55.600 usec
DE 6.00 usec
TE 673.2 K
D1 1.00000000 sec
MCREST 0.00000000 sec
MCWRK 0.01500000 sec
======== CHANNEL f1 ========
NUC1 1H
P1 7.00 usec
PL1 -1.00 dB
SFO1 300.1324010 MHz
F2 - Processing parameters
SI 32768
SF 300.1300000 MHz
WDW EM
SSB 0
LB 0.30 Hz
GB 0
PC 1.00

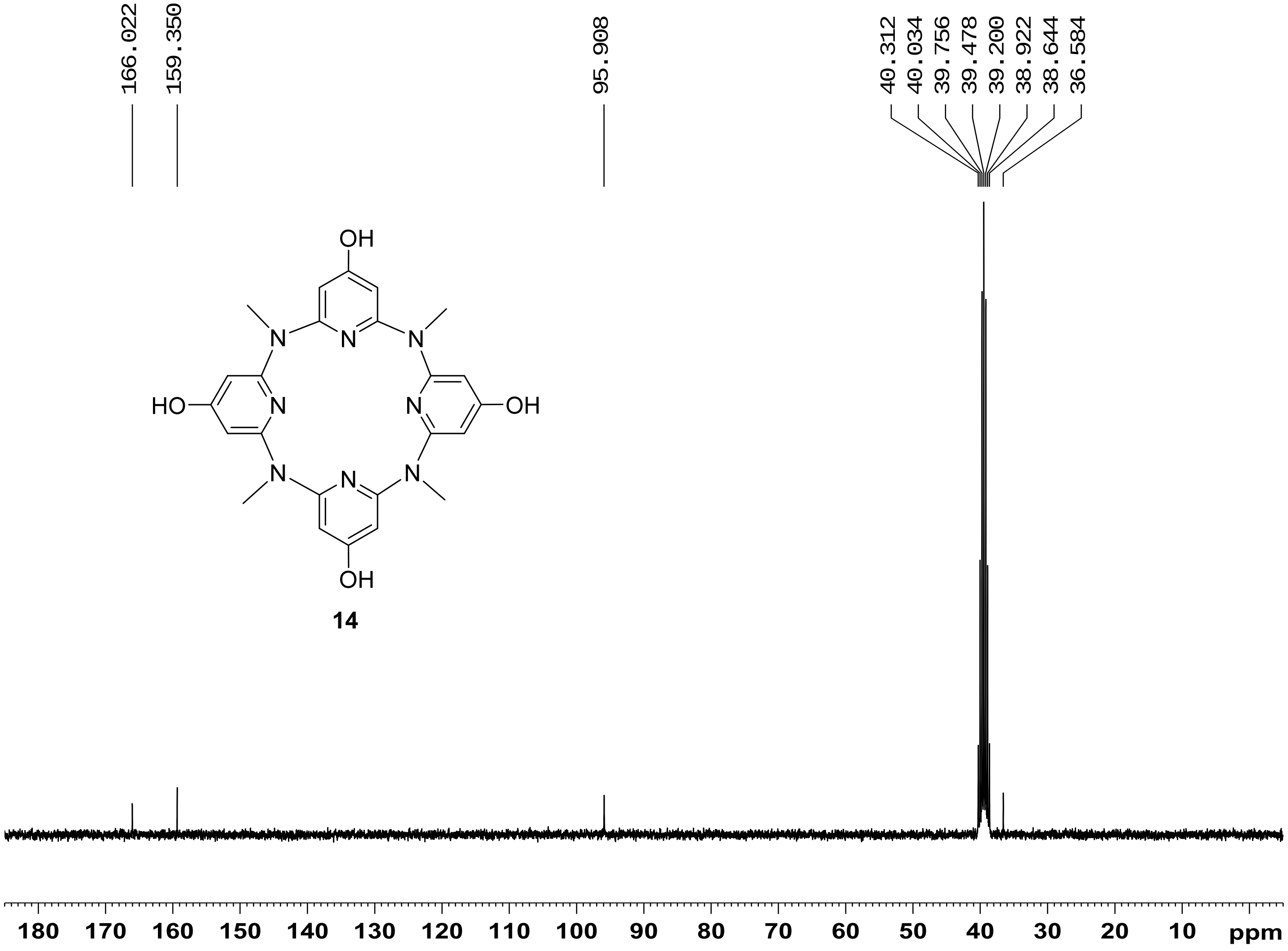


```
Current Data Parameters
NAME          zhex-222-2
EXPNO                 11
PROCNO                 1

F2 - Acquisition Parameters
Date_           20091116
Time               20.17
INSTRUM            spect
PROBHD   5 mm DUL 13C-1
PULPROG          zgpg30
TD                65536
SOLVENT             DMSO
NS                   257
DS                     4
SWH           17985.611 Hz
FIDRES         0.274439 Hz
AQ            1.8219508 sec
RG                 13004
DW                27.800 usec
DE                  8.00 usec
TE                 296.7 K
D1            2.00000000 sec
D11           0.03000000 sec
TD0                    1

======== CHANNEL f1 ========
NUC1                 13C
P1                 12.50 usec
PL1                 2.00 dB
SFO1         75.4752953 MHz

======== CHANNEL f2 ========
CPDPRG2          waltz16
NUC2                  1H
PCPD2             100.00 usec
PL2                 3.00 dB
PL12               22.33 dB
PL13               23.00 dB
SFO2         300.1312005 MHz

F2 - Processing parameters
SI                 32768
SF            75.4677867 MHz
WDW                   EM
SSB                    0
LB                  1.00 Hz
GB                     0
PC                  1.40
```

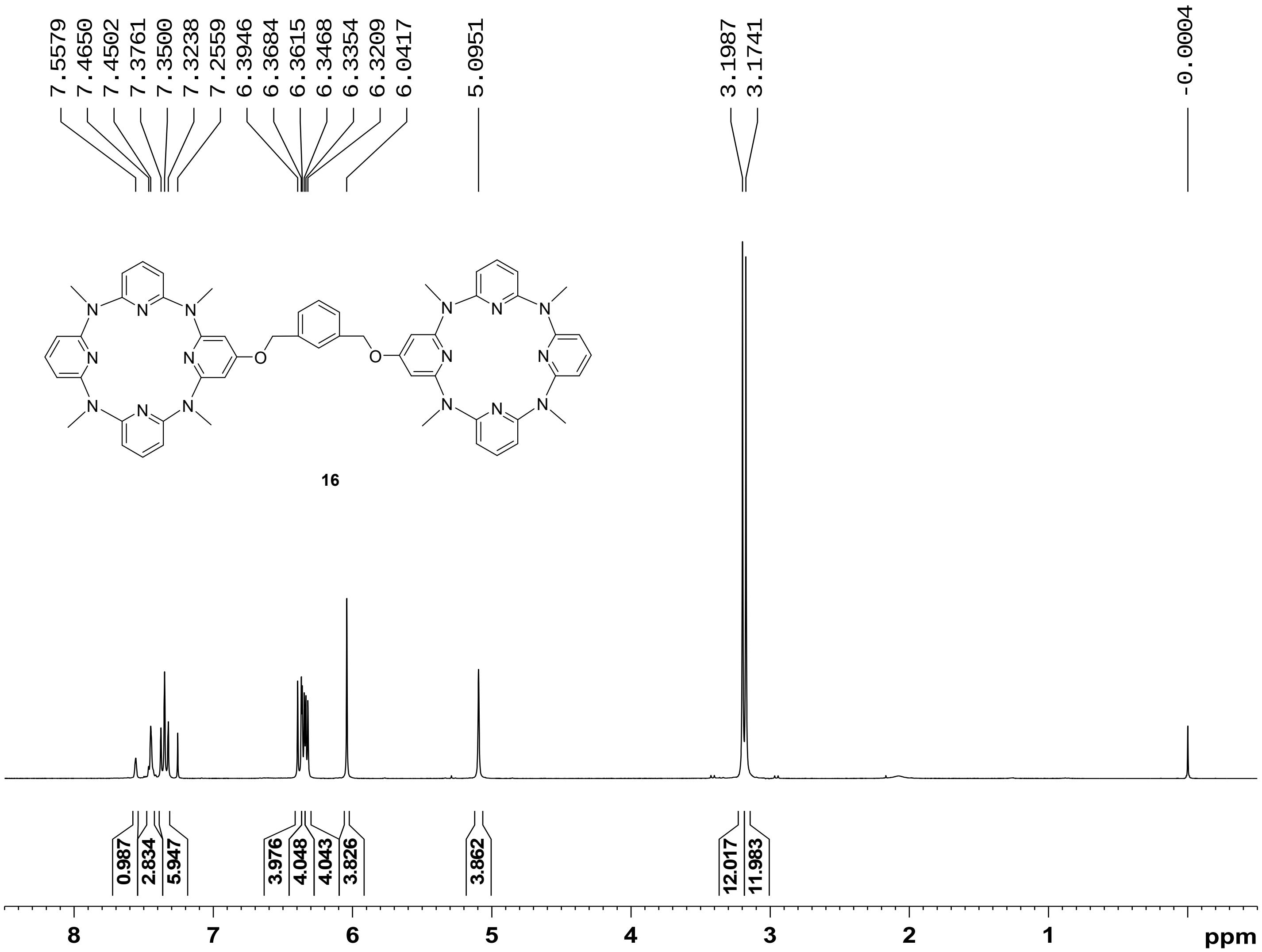


```
Current Data Parameters
NAME             zhex-252
EXPNO                  91
PROCNO                  1

F2 - Acquisition Parameters
Date_            20100208
Time                14.11
INSTRUM             spect
PROBHD   5 mm DUL 13C-1
PULPROG              zg30
TD                  65536
SOLVENT             CDCl3
NS                     16
DS                      0
SWH              8992.806 Hz
FIDRES           0.137219 Hz
AQ              3.6438515 sec
RG                    256
DW                 55.600 usec
DE                   8.00 usec
TE                  295.0 K
D1             1.00000000 sec
TD0                     1

======== CHANNEL f1 ========
NUC1                   1H
P1                  10.80 usec
PL1                  3.00 dB
SFO1          300.1324010 MHz

F2 - Processing parameters
SI                  32768
SF            300.1300072 MHz
WDW                    EM
SSB                     0
LB                   0.30 Hz
GB                      0
PC                   1.00
```

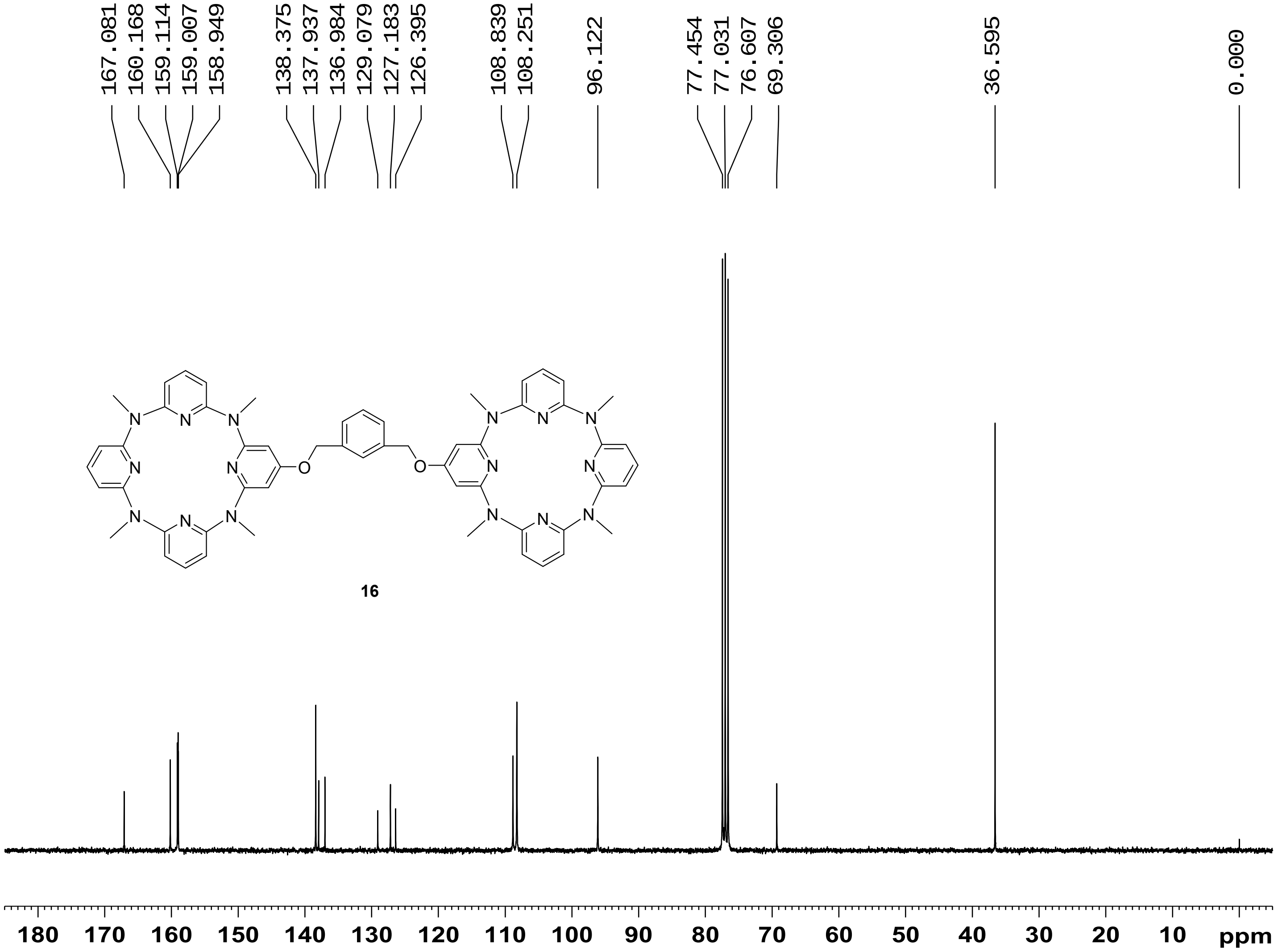


```
Current Data Parameters
NAME            zhex-252
EXPNO                 90
PROCNO                 1

F2 - Acquisition Parameters
Date_           20100208
Time               12.21
INSTRUM            spect
PROBHD   5 mm DUL 13C-1
PULPROG           zgpg30
TD                 65536
SOLVENT            CDCl3
NS                  1673
DS                     4
SWH            17985.611 Hz
FIDRES          0.274439 Hz
AQ             1.8219508 sec
RG               11585.2
DW                27.800 usec
DE                  8.00 usec
TE                 295.2 K
D1            2.00000000 sec
D11           0.03000000 sec
TD0                    1

======== CHANNEL f1 ========
NUC1                 13C
P1                 12.50 usec
PL1                 2.00 dB
SFO1          75.4752953 MHz

======== CHANNEL f2 ========
CPDPRG2          waltz16
NUC2                  1H
PCPD2             100.00 usec
PL2                 3.00 dB
PL12               22.33 dB
PL13               23.00 dB
SFO2         300.1312005 MHz

F2 - Processing parameters
SI                 32768
SF            75.4677504 MHz
WDW                   EM
SSB                    0
LB                  1.00 Hz
GB                     0
PC                  1.40
```

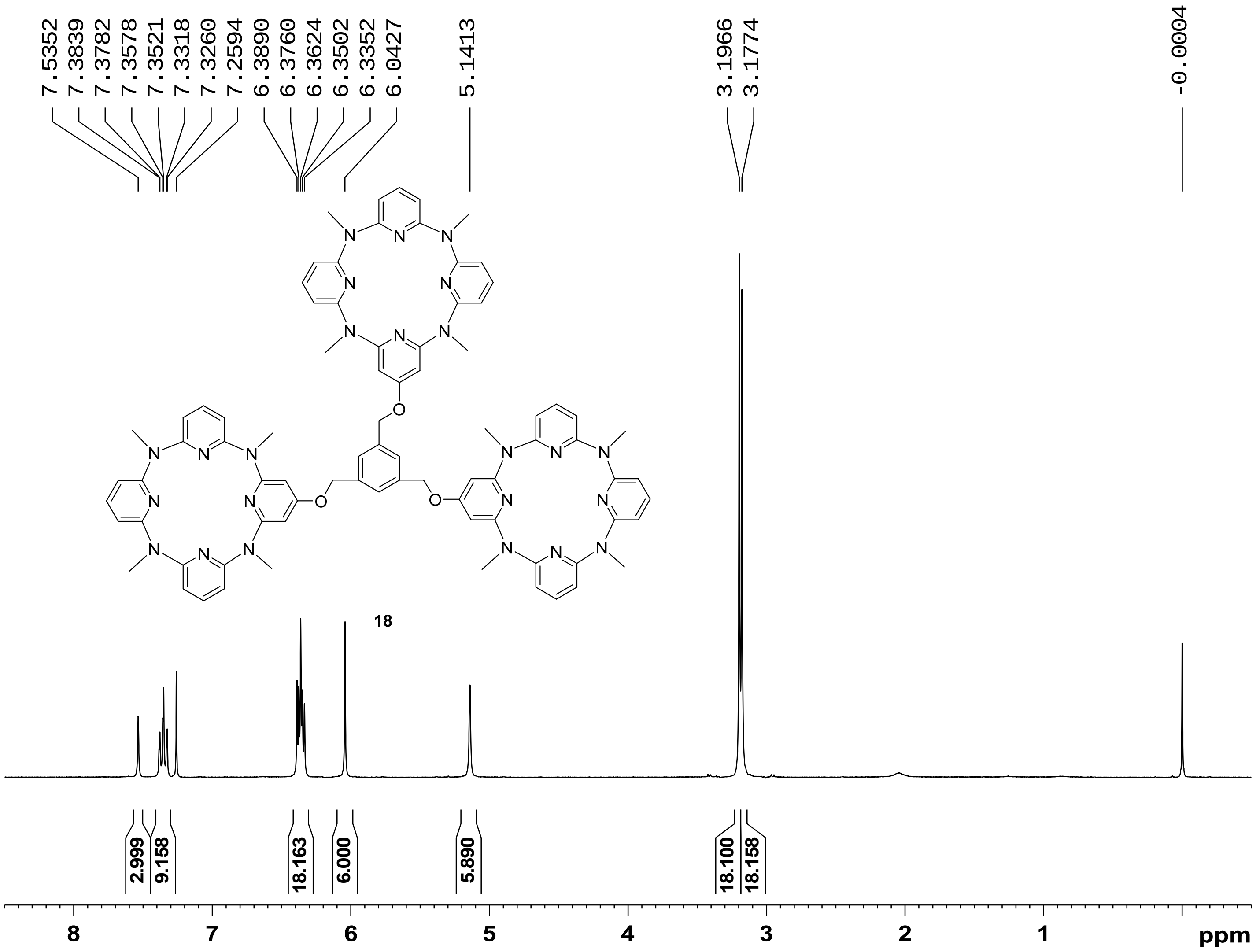


```
Current Data Parameters
NAME            zhex-256
EXPNO                 10
PROCNO                 1

F2 - Acquisition Parameters
Date_           20100206
Time                9.58
INSTRUM            spect
PROBHD   5 mm DUL 13C-1
PULPROG             zg30
TD                 65536
SOLVENT            CDCl3
NS                    16
DS                     0
SWH          8992.806 Hz
FIDRES       0.137219 Hz
AQ          3.6438515 sec
RG                 456.1
DW            55.600 usec
DE              8.00 usec
TE               294.9 K
D1        1.00000000 sec
TD0                    1

======== CHANNEL f1 ========
NUC1                  1H
P1             10.80 usec
PL1              3.00 dB
SFO1      300.1324010 MHz

F2 - Processing parameters
SI                 32768
SF        300.1300062 MHz
WDW                   EM
SSB                    0
LB               0.30 Hz
GB                     0
PC                  1.00
```

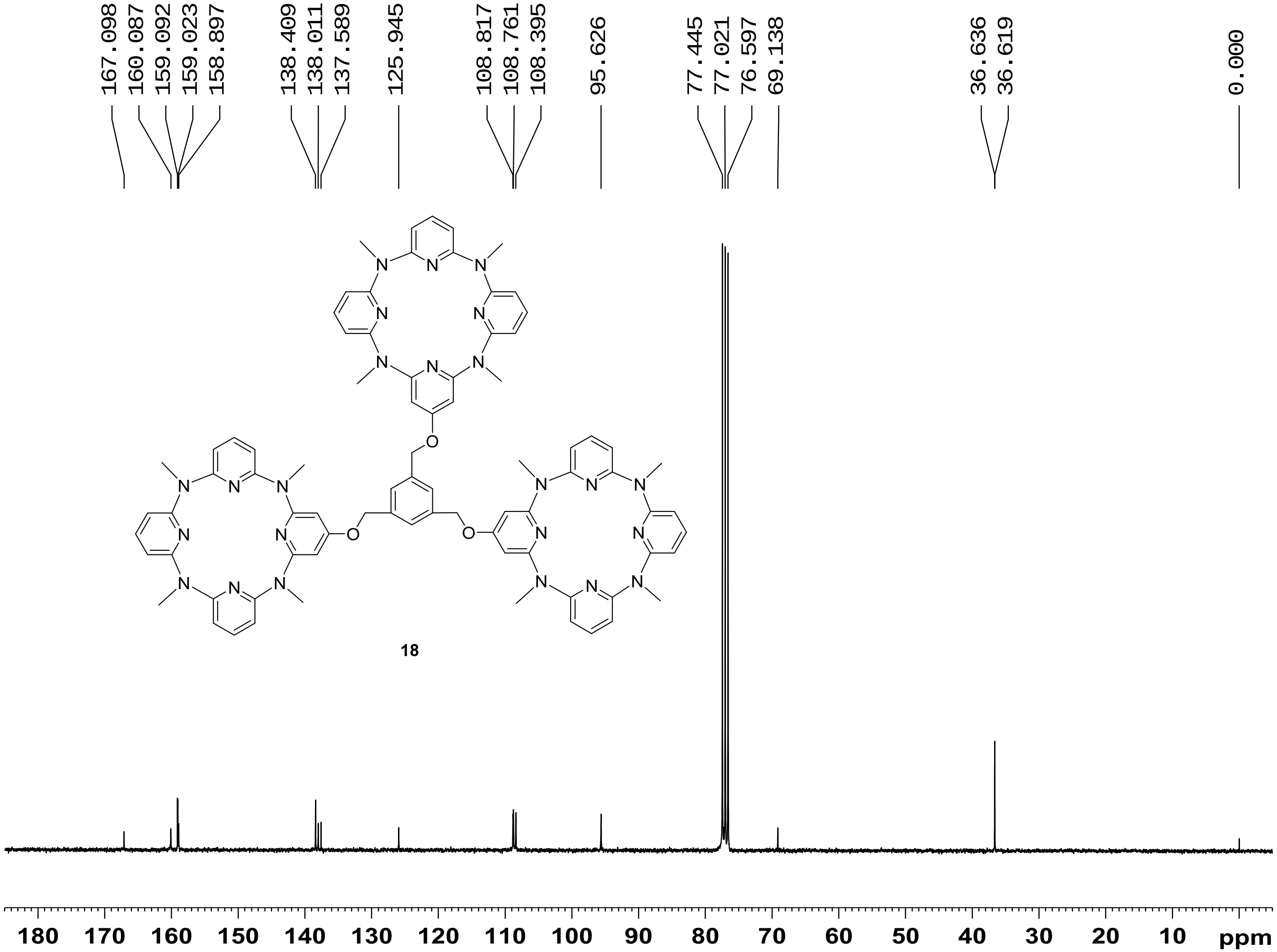

Current Data Parameters
NAME zhex-256
EXPNO 12
PROCNO 1

F2 - Acquisition Parameters
Date_ 20100206
Time 10.10
INSTRUM spect
PROBHD 5 mm DUL 13C-1
PULPROG zgpg30
TD 65536
SOLVENT CDCl3
NS 3533
DS 4
SWH 17985.611 Hz
FIDRES 0.274439 Hz
AQ 1.8219508 sec
RG 14596.5
DW 27.800 usec
DE 8.00 usec
TE 295.4 K
D1 2.00000000 sec
D11 0.03000000 sec
TD0 1

======== CHANNEL f1 ========
NUC1 13C
P1 12.50 usec
PL1 2.00 dB
SFO1 75.4752953 MHz

======== CHANNEL f2 ========
CPDPRG2 waltz16
NUC2 1H
PCPD2 100.00 usec
PL2 3.00 dB
PL12 22.33 dB
PL13 23.00 dB
SFO2 300.1312005 MHz

F2 - Processing parameters
SI 32768
SF 75.4677494 MHz
WDW EM
SSB 0
LB 1.00 Hz
GB 0
PC 1.40

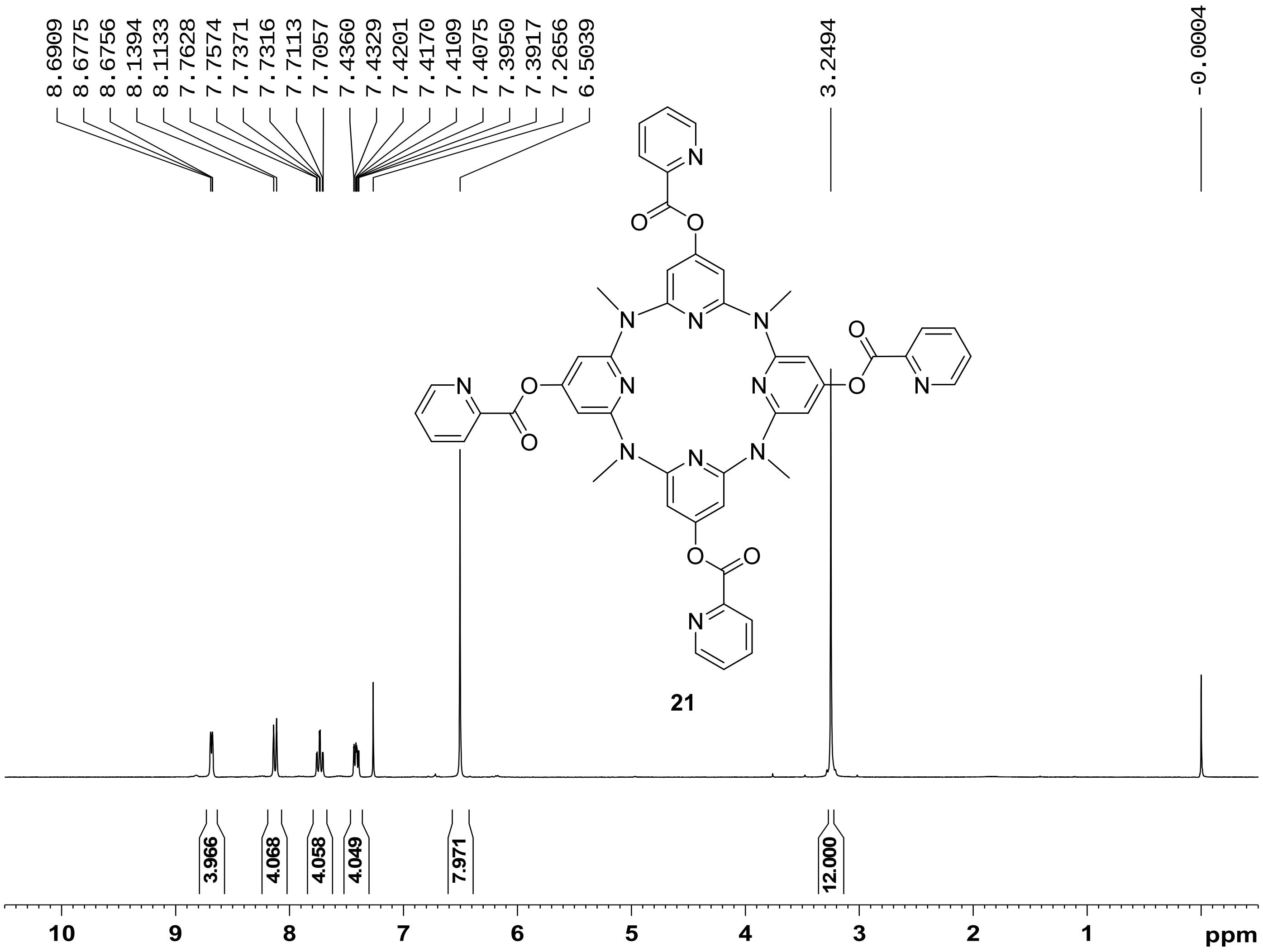


```
Current Data Parameters
NAME           zhex-333
EXPNO                20
PROCNO                1

F2 - Acquisition Parameters
Date_          20100308
Time              22.13
INSTRUM           spect
PROBHD   5 mm DUL 13C-1
PULPROG            zg30
TD                65536
SOLVENT           CDCl3
NS                   16
DS                    0
SWH            8992.806 Hz
FIDRES         0.137219 Hz
AQ            3.6438515 sec
RG                574.7
DW               55.600 usec
DE                 8.00 usec
TE                297.2 K
D1           1.00000000 sec
TD0                   1

======== CHANNEL f1 ========
NUC1                 1H
P1                10.80 usec
PL1                3.00 dB
SFO1        300.1324010 MHz

F2 - Processing parameters
SI                32768
SF          300.1300044 MHz
WDW                  EM
SSB                   0
LB                 0.30 Hz
GB                    0
PC                 1.00
```

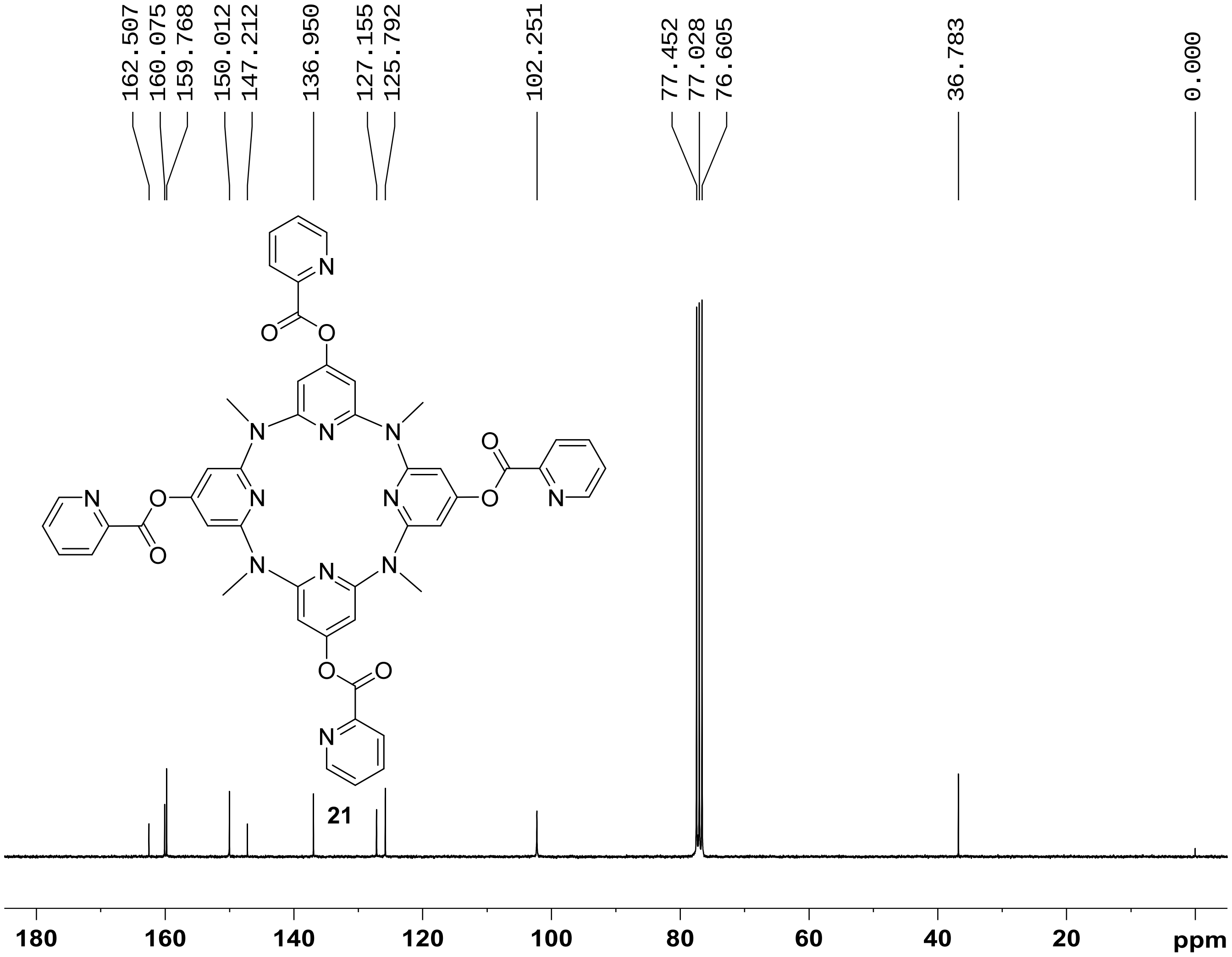


```
Current Data Parameters
NAME             zhex-333
EXPNO                  30
PROCNO                  1

F2 - Acquisition Parameters
Date_            20100309
Time                 6.44
INSTRUM             spect
PROBHD   5 mm DUL 13C-1
PULPROG           zgpg30
TD                  65536
SOLVENT             CDCl3
NS                   7029
DS                      4
SWH             17985.611 Hz
FIDRES           0.274439 Hz
AQ              1.8219508 sec
RG                   8192
DW                 27.800 usec
DE                   8.00 usec
TE                  297.2 K
D1             2.00000000 sec
D11            0.03000000 sec
TD0                     1

======== CHANNEL f1 ========
NUC1                  13C
P1                  12.50 usec
PL1                  2.00 dB
SFO1           75.4752953 MHz

======== CHANNEL f2 ========
CPDPRG2           waltz16
NUC2                   1H
PCPD2              100.00 usec
PL2                  3.00 dB
PL12                22.33 dB
PL13                23.00 dB
SFO2          300.1312005 MHz

F2 - Processing parameters
SI                  32768
SF             75.4677482 MHz
WDW                    EM
SSB                     0
LB                   1.00 Hz
GB                      0
PC                   1.40
```

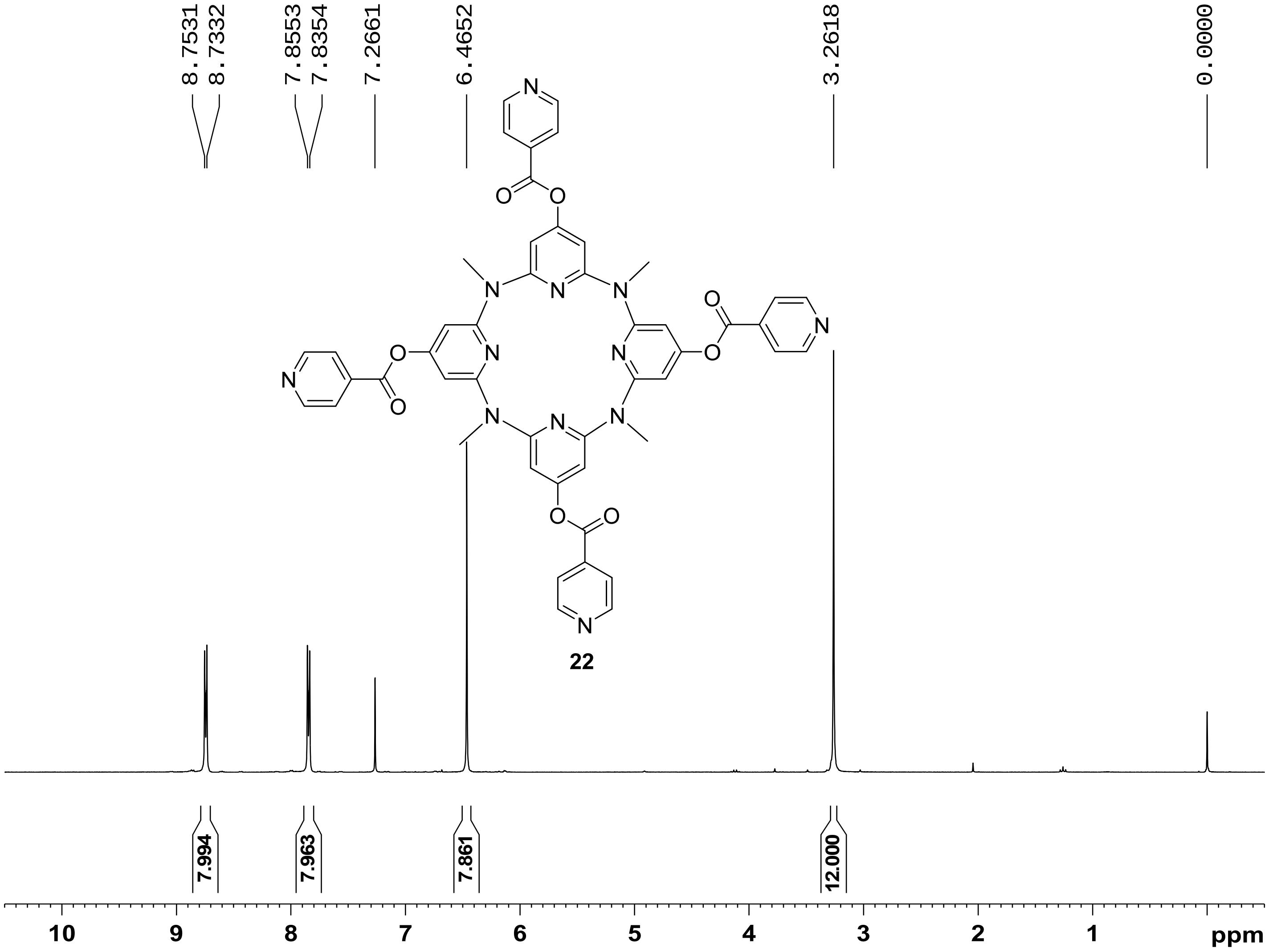


```
Current Data Parameters
NAME             zhex-329
EXPNO                  70
PROCNO                  1

F2 - Acquisition Parameters
Date_            20100313
Time                11.03
INSTRUM             spect
PROBHD   5 mm DUL 13C-1
PULPROG              zg30
TD                  32768
SOLVENT             CDCl3
NS                     16
DS                      0
SWH              8992.806 Hz
FIDRES           0.274439 Hz
AQ              1.8219508 sec
RG                  456.1
DW                 55.600 usec
DE                   8.00 usec
TE                  298.4 K
D1             2.00000000 sec
TD0                     1

======== CHANNEL f1 ========
NUC1                   1H
P1                  10.80 usec
PL1                  3.00 dB
SFO1          300.1318008 MHz

F2 - Processing parameters
SI                  32768
SF            300.1300042 MHz
WDW                    EM
SSB                     0
LB                   0.30 Hz
GB                      0
PC                   1.00
```

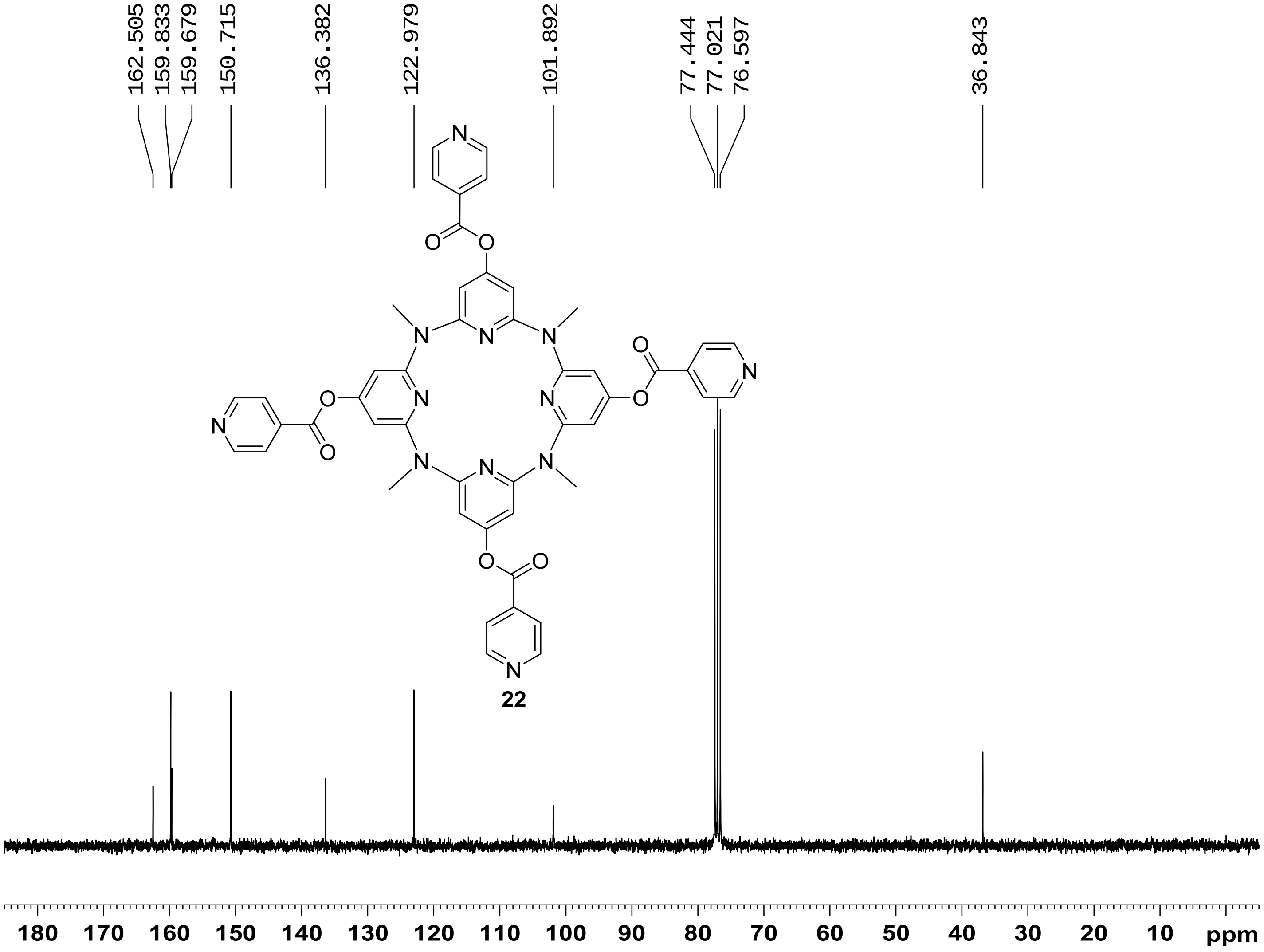


```
Current Data Parameters
NAME              zhex-329
EXPNO                   71
PROCNO                   1

F2 - Acquisition Parameters
Date_             20100313
Time                 11.11
INSTRUM              spect
PROBHD   5 mm DUL 13C-1
PULPROG            zgig30
TD                   32768
SOLVENT              CDCl3
NS                     402
DS                       0
SWH           18832.393 Hz
FIDRES         0.574719 Hz
AQ           0.8700404 sec
RG                   13004
DW            26.550 usec
DE              8.00 usec
TE               298.7 K
D1        2.00000000 sec
D11       0.03000000 sec
TD0                      1

======== CHANNEL f1 ========
NUC1                   13C
P1             12.50 usec
PL1              2.00 dB
SFO1       75.4752953 MHz

======== CHANNEL f2 ========
CPDPRG2            waltz16
NUC2                    1H
PCPD2         100.00 usec
PL2              3.00 dB
PL12            22.33 dB
SFO2      300.1312005 MHz

F2 - Processing parameters
SI                   32768
SF         75.4677490 MHz
WDW                     EM
SSB                      0
LB               1.00 Hz
GB                       0
PC                    1.40
```

7. Crystallographic data

Table S2 Crystallographic data of **10** and **14'**

	**10**	**14'**
ccdc	1939813	1939814
empirical formula	$C_{26}H_{30}N_8O_2S$	$C_{24}H_{28}N_8O_6$
M_r	518.64	524.54
crystal size [mm^3]	0.25 × 0.14 × 0.09	0.13 × 0.12 × 0.10
crystal system	triclinic	monoclinic
space group	P1	C2/c
a [Å]	12.267(3)	17.767(4)
b [Å]	13.680(3)	8.4770(17)
c [Å]	17.162(3)	17.343(4)

α [deg]	67.26(3)	90
β [deg]	85.66(3)	107.49(3)
γ [deg]	89.71(3)	90
V [Å^3]	2647.5(9)	2491.2(9)
d [g/cm^3]	1.301	1.399
Z	4	4
T [K]	173(2)	173(2)
R1, wR2 [I>2σ(I)]	0.0688, 0.1777	0.0754, 0.1646
R1, wR2 (all data)	0.0762, 0.1851	0.0902, 0.1748
quality of fit	1.063	1.165

Table S3 Crystallographic data of **21** and **22**

	**21**	**22**
ccdc	1939815	1939816
empirical formula	$C_{98}H_{75}N_{25}O_{16}$	$C_{48}H_{38}N_{12}O_8$
M_r	1858.83	910.9
crystal size [mm^3]	0.17 × 0.15 × 0.1	0.45 × 0.21 × 0.21
crystal system	monoclinic	tetragonal
space group	P12/C1	I 41/a
a [Å]	16.484(8)	12.1150(6)
b [Å]	8.058(4)	12.1150(6)
c [Å]	34.3607(18)	66.776(5)
α [deg]	90	90
β [deg]	96.057(5)	90

γ [deg]	90	90
V [Å^3]	4538(3)	9800.9(12)
d [g/cm^3]	1.360	1.235
Z	2	8
T [K]	173(2)	173(2)
R1, wR2 [I>2σ(I)]	0.1249, 0.2836	0.1083, 0.2312
R1, wR2 (all data)	0.1332, 0.2888	0.1104, 0.2328
quality of fit	1.288	1.295
