# Supplementary material for: Hydroxy-Substituted Azacalix[4]Pyridines: Synthesis, Structure, and Construction of Functional Architectures
Source: Front Chem. 2019 Aug 16;7:553. doi: 10.3389/fchem.2019.00553 (PMC6707087; doi:10.3389/fchem.2019.00553)

checkCIF/PLATON report

You have not supplied any structure factors. As a result the full set of tests cannot be run.

THIS REPORT IS FOR GUIDANCE ONLY. IF USED AS PART OF A REVIEW PROCEDURE FOR PUBLICATION, IT SHOULD NOT REPLACE THE EXPERTISE OF AN EXPERIENCED CRYSTALLOGRAPHIC REFEREE.

No syntax errors found. CIF dictionary Interpreting this report

Datablock: mx200

```
Bond precision:  C-C = 0.0059 A          Wavelength=0.71073

Cell:           a=12.267(3)       b=13.680(3)      c=17.162(3)
                alpha=67.26(3)    beta=85.66(3)    gamma=89.71(3)
Temperature:    173 K

                Calculated                 Reported
Volume          2647.6(12)                 2647.5(9)
Space group     P 1                        P1
Hall group      P 1                        ?
Moiety formula  C24 H24 N8 O, C2 H6 O S    ?
Sum formula     C26 H30 N8 O2 S            C26 H30 N8 O2 S
Mr              518.64                     518.64
Dx,g cm-3       1.301                      1.301
Z               4                          4
Mu (mm-1)       0.162                      0.162
F000            1096.0                     1096.0
F000'           1096.85
h,k,lmax        15,17,22                   15,17,22
Nref            24254[ 12127]              23971
Tmin,Tmax       0.973,0.986                0.961,0.986
Tmin'           0.960

Correction method= # Reported T Limits: Tmin=0.961 Tmax=0.986
AbsCorr = NUMERICAL

Data completeness= 1.98/0.99      Theta(max)= 27.470

R(reflections)= 0.0688( 21101)    wR2(reflections)= 0.1851( 23971)

S = 1.063                  Npar= 1438
```

The following ALERTS were generated. Each ALERT has the format
test-name_ALERT_alert-type_alert-level.
Click on the hyperlinks for more details of the test.

Alert level C

```
PLAT234_ALERT_4_C Large Hirshfeld Difference N2       --C21'     .       0.16 Ang.
PLAT260_ALERT_2_C Large Average Ueq of Residue Including        S4      0.106 Check
PLAT260_ALERT_2_C Large Average Ueq of Residue Including       S2'      0.103 Check
PLAT260_ALERT_2_C Large Average Ueq of Residue Including       S4'      0.167 Check
PLAT340_ALERT_3_C Low Bond Precision on  C-C Bonds ...............    0.00592 Ang.
PLAT412_ALERT_2_C Short Intra XH3 .. XHn     H2A       ..H24A     .       1.84 Ang.
                                                       x,y,z  =      1_555 Check
PLAT412_ALERT_2_C Short Intra XH3 .. XHn     H12A      ..H22A     .       1.87 Ang.
                                                       x,y,z  =      1_555 Check
PLAT412_ALERT_2_C Short Intra XH3 .. XHn     H2BA      ..H24D     .       1.88 Ang.
                                                       x,y,z  =      1_555 Check
PLAT412_ALERT_2_C Short Intra XH3 .. XHn     H4BA      ..H21G     .       1.85 Ang.
                                                       x,y,z  =      1_555 Check
PLAT412_ALERT_2_C Short Intra XH3 .. XHn     H12B      ..H22D     .       1.87 Ang.
                                                       x,y,z  =      1_555 Check
PLAT412_ALERT_2_C Short Intra XH3 .. XHn     H14B      ..H23D     .       1.82 Ang.
                                                       x,y,z  =      1_555 Check
PLAT412_ALERT_2_C Short Intra XH3 .. XHn     H2CA      ..H24G     .       1.84 Ang.
                                                       x,y,z  =      1_555 Check
PLAT412_ALERT_2_C Short Intra XH3 .. XHn     H4CA      ..H21J     .       1.85 Ang.
                                                       x,y,z  =      1_555 Check
PLAT412_ALERT_2_C Short Intra XH3 .. XHn     H12C      ..H22G     .       1.86 Ang.
                                                       x,y,z  =      1_555 Check
PLAT412_ALERT_2_C Short Intra XH3 .. XHn     H14C      ..H23G     .       1.86 Ang.
                                                       x,y,z  =      1_555 Check
PLAT412_ALERT_2_C Short Intra XH3 .. XHn     H2DA      ..H24J     .       1.87 Ang.
                                                       x,y,z  =      1_555 Check
PLAT412_ALERT_2_C Short Intra XH3 .. XHn     H4DA      ..H21M     .       1.88 Ang.
                                                       x,y,z  =      1_555 Check
PLAT413_ALERT_2_C Short Inter XH3 .. XHn     H19B      ..H22H     .       2.12 Ang.
                                                     1+x,y,z  =      1_655 Check
PLAT413_ALERT_2_C Short Inter XH3 .. XHn     H23E      ..H23K     .       2.01 Ang.
                                                    x,y,-1+z  =      1_554 Check
PLAT480_ALERT_4_C Long H...A H-Bond Reported H1C       ..S2       .       2.98 Ang.
PLAT480_ALERT_4_C Long H...A H-Bond Reported H1D       ..S1       .       2.91 Ang.
PLAT601_ALERT_2_C Structure Contains Solvent Accessible VOIDS of .          31 Ang**3
PLAT790_ALERT_4_C Centre of Gravity not Within Unit Cell: Resd.  #           1 Note
              C24 H24 N8 O
```

Alert level G

```
PLAT002_ALERT_2_G Number of Distance or Angle Restraints on AtSite          27 Note
PLAT003_ALERT_2_G Number of Uiso or Uij Restrained non-H Atoms ...          18 Report
PLAT005_ALERT_5_G No Embedded Refinement Details Found  in the CIF     Please Do !
PLAT007_ALERT_5_G Number of Unrefined Donor-H Atoms ..............           6 Report
PLAT066_ALERT_1_G Predicted and Reported Tmin&Tmax Range Identical           ? Check
PLAT112_ALERT_2_G ADDSYM Detects New (Pseudo) Symm. Elem        2           99 %Fit
PLAT113_ALERT_2_G ADDSYM Suggests Possible Pseudo/New  Space Group          C2 Check
PLAT152_ALERT_1_G The Supplied and Calc. Volume s.u. Differ by ...           3 Units
PLAT154_ALERT_1_G The s.u.'s on the Cell Angles are Equal ..(Note)        0.03 Degree
PLAT301_ALERT_3_G Main Residue  Disorder ..............(Resd  1  )          3% Note
PLAT301_ALERT_3_G Main Residue  Disorder ..............(Resd  2  )          3% Note
PLAT301_ALERT_3_G Main Residue  Disorder ..............(Resd  3  )          3% Note
PLAT302_ALERT_4_G Anion/Solvent/Minor-Residue Disorder (Resd  6  )        100% Note
PLAT302_ALERT_4_G Anion/Solvent/Minor-Residue Disorder (Resd  8  )        100% Note
PLAT302_ALERT_4_G Anion/Solvent/Minor-Residue Disorder (Resd  9  )        100% Note
PLAT302_ALERT_4_G Anion/Solvent/Minor-Residue Disorder (Resd 10  )        100% Note
PLAT304_ALERT_4_G Non-Integer Number of Atoms in ...... Resd  6           7.80 Check
PLAT304_ALERT_4_G Non-Integer Number of Atoms in ...... Resd  8           5.33 Check
PLAT304_ALERT_4_G Non-Integer Number of Atoms in ...... Resd  9           2.20 Check
```

```
PLAT304_ALERT_4_G Non-Integer Number of Atoms in ...... Resd 10          4.67 Check
PLAT380_ALERT_4_G Incorrectly? Oriented X(sp2)-Methyl Moiety .....        C22 Check
PLAT380_ALERT_4_G Incorrectly? Oriented X(sp2)-Methyl Moiety .....        C24 Check
PLAT380_ALERT_4_G Incorrectly? Oriented X(sp2)-Methyl Moiety .....       C24B Check
PLAT380_ALERT_4_G Incorrectly? Oriented X(sp2)-Methyl Moiety .....       C21C Check
PLAT380_ALERT_4_G Incorrectly? Oriented X(sp2)-Methyl Moiety .....       C24C Check
PLAT380_ALERT_4_G Incorrectly? Oriented X(sp2)-Methyl Moiety .....       C22D Check
PLAT380_ALERT_4_G Incorrectly? Oriented X(sp2)-Methyl Moiety .....       C23D Check
PLAT380_ALERT_4_G Incorrectly? Oriented X(sp2)-Methyl Moiety .....       C24D Check
PLAT432_ALERT_2_G Short Inter X...Y Contact  N3B      ..C27'             2.94 Ang.
                                                x,-1+y,z  =      1_545 Check
PLAT720_ALERT_4_G Number of Unusual/Non-Standard Labels ..........         17 Note
PLAT779_ALERT_4_G Suspect or Irrelevant (Bond) Angle in CIF .... #         98 Check
              C21' -N2   -C21     1.555   1.555   1.555              40.20 Deg.
PLAT779_ALERT_4_G Suspect or Irrelevant (Bond) Angle in CIF .... #        202 Check
              C3B  -O1B  -H3BA    1.555   1.555   1.555               9.30 Deg.
PLAT779_ALERT_4_G Suspect or Irrelevant (Bond) Angle in CIF .... #        205 Check
              C13B -O1E  -H13C    1.555   1.555   1.555              17.00 Deg.
PLAT779_ALERT_4_G Suspect or Irrelevant (Bond) Angle in CIF .... #        232 Check
              O1B  -C3B  -H3BA    1.555   1.555   1.555               3.80 Deg.
PLAT779_ALERT_4_G Suspect or Irrelevant (Bond) Angle in CIF .... #        265 Check
              O1E  -C13B -H13C    1.555   1.555   1.555               7.00 Deg.
PLAT779_ALERT_4_G Suspect or Irrelevant (Bond) Angle in CIF .... #        314 Check
              C3C  -O1C  -H3CA    1.555   1.555   1.555               5.30 Deg.
PLAT779_ALERT_4_G Suspect or Irrelevant (Bond) Angle in CIF .... #        317 Check
              C13C -O1F  -H13D    1.555   1.555   1.555              35.50 Deg.
PLAT779_ALERT_4_G Suspect or Irrelevant (Bond) Angle in CIF .... #        346 Check
              O1C  -C3C  -H3CA    1.555   1.555   1.555               2.30 Deg.
PLAT779_ALERT_4_G Suspect or Irrelevant (Bond) Angle in CIF .... #        379 Check
              O1F  -C13C -H13D    1.555   1.555   1.555              23.60 Deg.
PLAT790_ALERT_4_G Centre of Gravity not Within Unit Cell: Resd.  #          2 Note
              C24 H24 N8 O
PLAT790_ALERT_4_G Centre of Gravity not Within Unit Cell: Resd.  #          3 Note
              C24 H24 N8 O
PLAT790_ALERT_4_G Centre of Gravity not Within Unit Cell: Resd.  #          4 Note
              C24 H24 N8 O
PLAT790_ALERT_4_G Centre of Gravity not Within Unit Cell: Resd.  #          6 Note
              C2 H6 O S
PLAT790_ALERT_4_G Centre of Gravity not Within Unit Cell: Resd.  #          8 Note
              C2 H6 O S
PLAT790_ALERT_4_G Centre of Gravity not Within Unit Cell: Resd.  #          9 Note
              C2 H6 O S
PLAT790_ALERT_4_G Centre of Gravity not Within Unit Cell: Resd.  #         10 Note
              C2 H6 O S
PLAT860_ALERT_3_G Number of Least-Squares Restraints .............        184 Note
PLAT899_ALERT_4_G SHELXL97   is Deprecated and Succeeded by SHELXL       2018 Note
```

---

   0 **ALERT level A** = Most likely a serious problem - resolve or explain
   0 **ALERT level B** = A potentially serious problem, consider carefully
  23 **ALERT level C** = Check. Ensure it is not caused by an omission or oversight
  48 **ALERT level G** = General information/check it is not something unexpected

```
   3 ALERT type 1 CIF construction/syntax error, inconsistent or missing data
  23 ALERT type 2 Indicator that the structure model may be wrong or deficient
   5 ALERT type 3 Indicator that the structure quality may be low
  38 ALERT type 4 Improvement, methodology, query or suggestion
   2 ALERT type 5 Informative message, check
```

It is advisable to attempt to resolve as many as possible of the alerts in all categories. Often the minor alerts point to easily fixed oversights, errors and omissions in your CIF or refinement strategy, so attention to these fine details can be worthwhile. In order to resolve some of the more serious problems it may be necessary to carry out additional measurements or structure refinements. However, the purpose of your study may justify the reported deviations and the more serious of these should normally be commented upon in the discussion or experimental section of a paper or in the "special_details" fields of the CIF. checkCIF was carefully designed to identify outliers and unusual parameters, but every test has its limitations and alerts that are not important in a particular case may appear. Conversely, the absence of alerts does not guarantee there are no aspects of the results needing attention. It is up to the individual to critically assess their own results and, if necessary, seek expert advice.

Publication of your CIF in IUCr journals

A basic structural check has been run on your CIF. These basic checks will be run on all CIFs submitted for publication in IUCr journals (*Acta Crystallographica*, *Journal of Applied Crystallography*, *Journal of Synchrotron Radiation*); however, if you intend to submit to *Acta Crystallographica Section C* or *E* or *IUCrData*, you should make sure that full publication checks are run on the final version of your CIF prior to submission.

Publication of your CIF in other journals

Please refer to the *Notes for Authors* of the relevant journal for any special instructions relating to CIF submission.

PLATON version of 03/05/2019; check.def file version of 29/04/2019

Datablock mx200 - ellipsoid plot

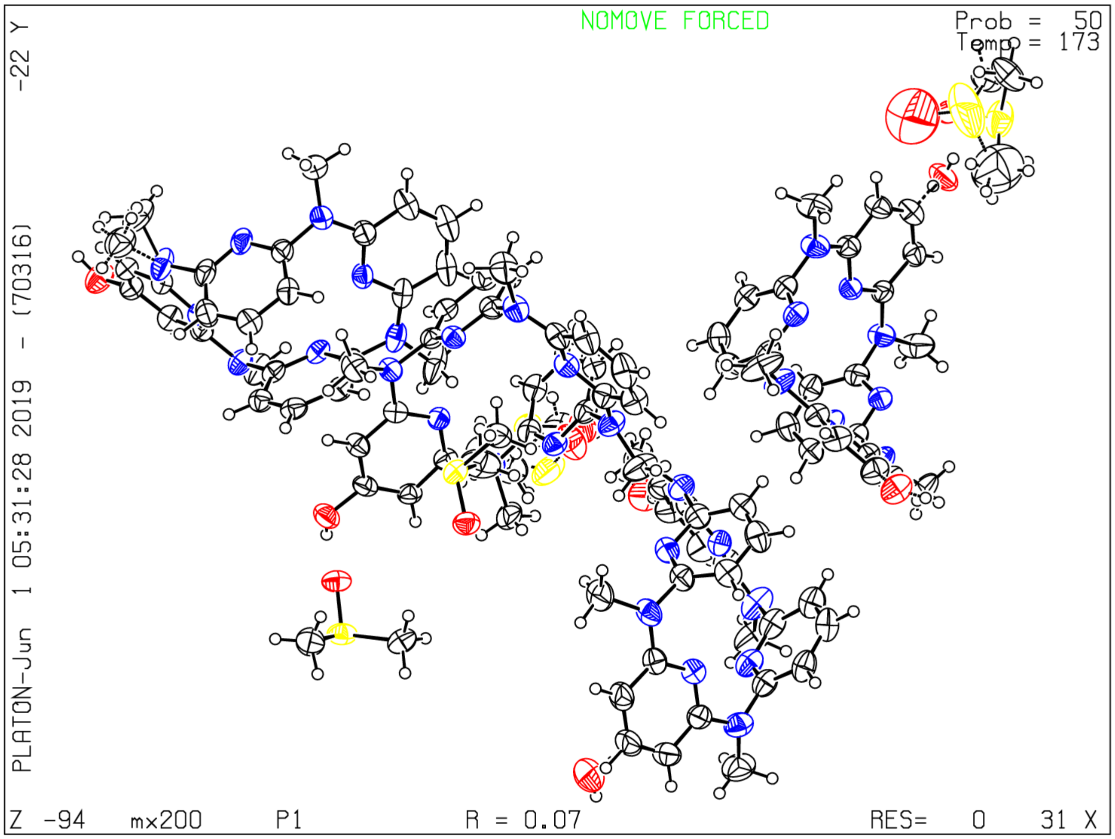

Supplement: Supplementary file 2 [file Data_Sheet_2.PDF]
