# Supplementary material for: Hydroxy-Substituted Azacalix[4]Pyridines: Synthesis, Structure, and Construction of Functional Architectures
Source: Front Chem. 2019 Aug 16;7:553. doi: 10.3389/fchem.2019.00553 (PMC6707087; doi:10.3389/fchem.2019.00553)

checkCIF/PLATON report

Structure factors have been supplied for datablock(s) sa657

THIS REPORT IS FOR GUIDANCE ONLY. IF USED AS PART OF A REVIEW PROCEDURE FOR PUBLICATION, IT SHOULD NOT REPLACE THE EXPERTISE OF AN EXPERIENCED CRYSTALLOGRAPHIC REFEREE.

No syntax errors found. CIF dictionary Interpreting this report

Datablock: sa657

```
Bond precision:  C-C = 0.0085 A          Wavelength=0.71073

Cell:            a=16.484(8)     b=8.058(4)          c=34.3607(18)
                 alpha=90        beta=96.057(5)      gamma=90
Temperature:     173 K
```

	Calculated	Reported
Volume	4539(3)	4538(3)
Space group	P 2/c	P 1 2/c 1
Hall group	-P 2yc	-P 2yc
Moiety formula	2(C48 H36 N12 O8), C2 H3 N	2(C48 H36 N12 O8), 0.5(C4 H6 N2)
Sum formula	C98 H75 N25 O16	C98 H75 N25 O16
Mr	1858.83	1858.83
Dx,g cm-3	1.360	1.360
Z	2	2
Mu (mm-1)	0.096	0.096
F000	1932.0	1932.0
F000’	1932.84	
h,k,lmax	19,9,40	19,9,40
Nref	8001	7963
Tmin,Tmax	0.984,0.990	0.783,1.000
Tmin’	0.984	

```
Correction method= # Reported T Limits: Tmin=0.783 Tmax=1.000
AbsCorr = MULTI-SCAN

Data completeness= 0.995          Theta(max)= 24.989

R(reflections)= 0.1249( 7410)     wR2(reflections)= 0.2888( 7963)

S = 1.288                   Npar= 725
```

The following ALERTS were generated. Each ALERT has the format
test-name_ALERT_alert-type_alert-level.
Click on the hyperlinks for more details of the test.

Alert level B

```
PLAT094_ALERT_2_B Ratio of Maximum / Minimum Residual Density ....       6.54 Report
PLAT097_ALERT_2_B Large Reported Max.  (Positive) Residual Density       1.52 eA-3
```

Alert level C

```
DIFMX02_ALERT_1_C  The maximum difference density is > 0.1*ZMAX*0.75
            The relevant atom site should be identified.
PLAT082_ALERT_2_C High R1 Value ..................................       0.12 Report
PLAT084_ALERT_3_C High wR2 Value (i.e. > 0.25) ...................       0.29 Report
PLAT244_ALERT_4_C Low    'Solvent' Ueq as Compared to Neighbors of        C50 Check
PLAT340_ALERT_3_C Low Bond Precision on  C-C Bonds ...............    0.00847 Ang.
PLAT906_ALERT_3_C Large K Value in the Analysis of Variance ......     31.583 Check
PLAT906_ALERT_3_C Large K Value in the Analysis of Variance ......      5.084 Check
PLAT906_ALERT_3_C Large K Value in the Analysis of Variance ......      2.334 Check
PLAT910_ALERT_3_C Missing # of FCF Reflection(s) Below Theta(Min).          8 Note
PLAT911_ALERT_3_C Missing FCF Refl Between Thmin & STh/L=    0.594         30 Report
PLAT918_ALERT_3_C Reflection(s) with I(obs) much Smaller I(calc) .          1 Check
PLAT971_ALERT_2_C Check Calcd Resid. Dens.  2.42A   From C42'            1.56 eA-3
```

Alert level G

```
PLAT002_ALERT_2_G Number of Distance or Angle Restraints on AtSite         20 Note
PLAT003_ALERT_2_G Number of Uiso or Uij Restrained non-H Atoms ...         20 Report
PLAT042_ALERT_1_G Calc. and Reported MoietyFormula Strings  Differ     Please Check
PLAT083_ALERT_2_G SHELXL Second Parameter in WGHT  Unusually Large      10.96 Why ?
PLAT172_ALERT_4_G The CIF-Embedded .res File Contains DFIX Records          1 Report
PLAT176_ALERT_4_G The CIF-Embedded .res File Contains SADI Records          4 Report
PLAT178_ALERT_4_G The CIF-Embedded .res File Contains SIMU Records          7 Report
PLAT186_ALERT_4_G The CIF-Embedded .res File Contains ISOR Records          2 Report
PLAT300_ALERT_4_G Atom Site Occupancy of H49A       Constrained at        0.5 Check
PLAT300_ALERT_4_G Atom Site Occupancy of H49B       Constrained at        0.5 Check
PLAT300_ALERT_4_G Atom Site Occupancy of H49C       Constrained at        0.5 Check
PLAT301_ALERT_3_G Main Residue  Disorder ..............(Resd  1  )        15% Note
PLAT432_ALERT_2_G Short Inter X...Y Contact  O4        ..C30             2.98 Ang.
                                                  -x,-y,1-z  =      3_556 Check
PLAT432_ALERT_2_G Short Inter X...Y Contact  O8        ..C31             2.93 Ang.
                                                    x,1+y,z  =      1_565 Check
PLAT720_ALERT_4_G Number of Unusual/Non-Standard Labels .........           2 Note
PLAT860_ALERT_3_G Number of Least-Squares Restraints ............         336 Note
PLAT909_ALERT_3_G Percentage of I>2sig(I) Data at Theta(Max) Still        81% Note
PLAT913_ALERT_3_G Missing # of Very Strong Reflections in FCF ....          1 Note
PLAT933_ALERT_2_G Number of OMIT Records in Embedded .res File ...         13 Note
PLAT978_ALERT_2_G Number C-C Bonds with Positive Residual Density.          3 Info
```

---

0 **ALERT level A** = Most likely a serious problem - resolve or explain
2 **ALERT level B** = A potentially serious problem, consider carefully
12 **ALERT level C** = Check. Ensure it is not caused by an omission or oversight
20 **ALERT level G** = General information/check it is not something unexpected

2 ALERT type 1 CIF construction/syntax error, inconsistent or missing data
11 ALERT type 2 Indicator that the structure model may be wrong or deficient
12 ALERT type 3 Indicator that the structure quality may be low
9 ALERT type 4 Improvement, methodology, query or suggestion
0 ALERT type 5 Informative message, check

It is advisable to attempt to resolve as many as possible of the alerts in all categories. Often the minor alerts point to easily fixed oversights, errors and omissions in your CIF or refinement strategy, so attention to these fine details can be worthwhile. In order to resolve some of the more serious problems it may be necessary to carry out additional measurements or structure refinements. However, the purpose of your study may justify the reported deviations and the more serious of these should normally be commented upon in the discussion or experimental section of a paper or in the "special_details" fields of the CIF. checkCIF was carefully designed to identify outliers and unusual parameters, but every test has its limitations and alerts that are not important in a particular case may appear. Conversely, the absence of alerts does not guarantee there are no aspects of the results needing attention. It is up to the individual to critically assess their own results and, if necessary, seek expert advice.

Publication of your CIF in IUCr journals

A basic structural check has been run on your CIF. These basic checks will be run on all CIFs submitted for publication in IUCr journals (*Acta Crystallographica*, *Journal of Applied Crystallography*, *Journal of Synchrotron Radiation*); however, if you intend to submit to *Acta Crystallographica Section C* or *E* or *IUCrData*, you should make sure that full publication checks are run on the final version of your CIF prior to submission.

Publication of your CIF in other journals

Please refer to the *Notes for Authors* of the relevant journal for any special instructions relating to CIF submission.

PLATON version of 03/05/2019; check.def file version of 29/04/2019

Datablock sa657 - ellipsoid plot

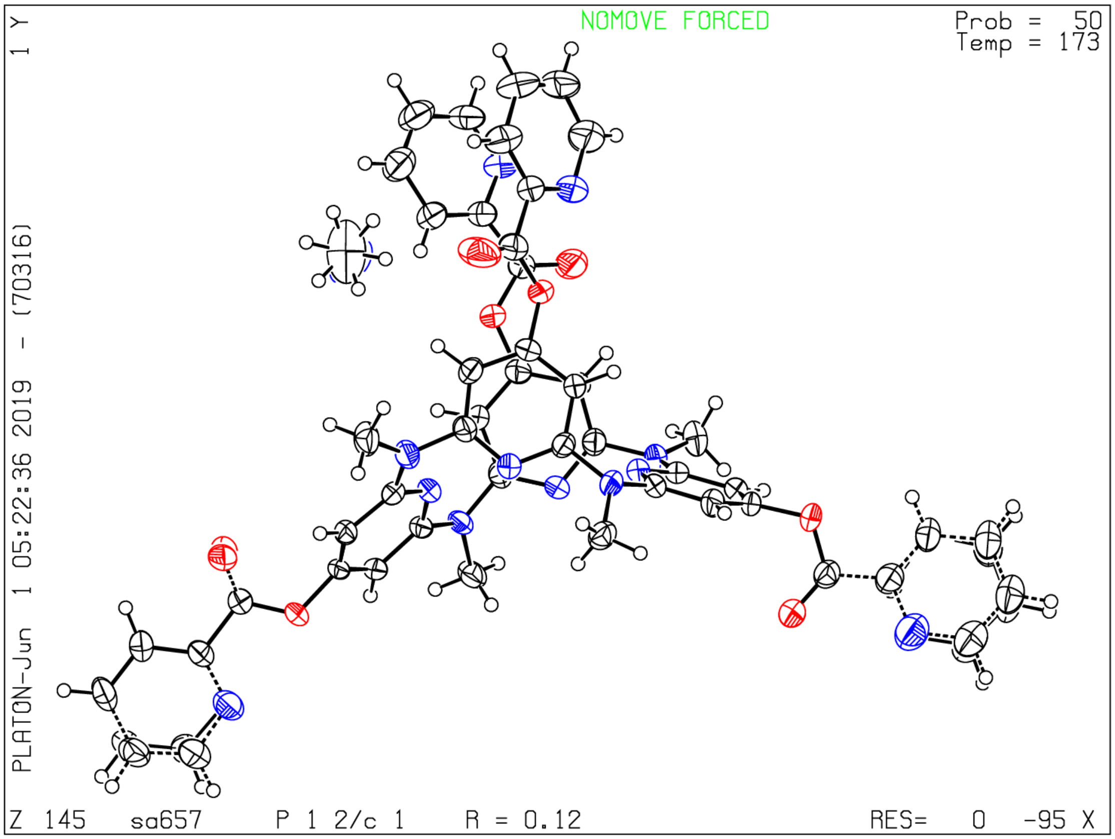

Supplement: Supplementary file 4 [file Data_Sheet_4.PDF]
