# Supplementary material for: Hydroxy-Substituted Azacalix[4]Pyridines: Synthesis, Structure, and Construction of Functional Architectures
Source: Front Chem. 2019 Aug 16;7:553. doi: 10.3389/fchem.2019.00553 (PMC6707087; doi:10.3389/fchem.2019.00553)

checkCIF/PLATON report

Structure factors have been supplied for datablock(s) sa670_sq

THIS REPORT IS FOR GUIDANCE ONLY. IF USED AS PART OF A REVIEW PROCEDURE FOR PUBLICATION, IT SHOULD NOT REPLACE THE EXPERTISE OF AN EXPERIENCED CRYSTALLOGRAPHIC REFEREE.

No syntax errors found. CIF dictionary Interpreting this report

Datablock: sa670_sq

```
Bond precision:  C-C = 0.0061 A          Wavelength=0.71073

Cell:             a=12.1150(6)      b=12.1150(6)      c=66.776(5)
                  alpha=90          beta=90           gamma=90
Temperature:      173 K

                Calculated                  Reported
Volume          9800.9(12)                  9800.9(12)
Space group     I 41/a                      I 41/a
Hall group      -I 4ad                      -I 4ad
Moiety formula  C48 H38 N12 O8 [+ solvent]  C48 H38 N12 O8
Sum formula     C48 H38 N12 O8 [+ solvent]  C48 H38 N12 O8
Mr              910.90                      910.90
Dx,g cm-3       1.235                       1.235
Z               8                           8
Mu (mm-1)       0.087                       0.087
F000            3792.0                      3792.0
F000’           3793.65
h,k,lmax        14,14,80                    14,14,80
Nref            4519                        4513
Tmin,Tmax       0.978,0.982                 0.765,1.000
Tmin’           0.962

Correction method= # Reported T Limits: Tmin=0.765 Tmax=1.000
AbsCorr = NUMERICAL

Data completeness= 0.999          Theta(max)= 25.332

R(reflections)= 0.1083( 4418)     wR2(reflections)= 0.2328( 4513)

S = 1.295                 Npar= 309
```

The following ALERTS were generated. Each ALERT has the format
test-name_ALERT_alert-type_alert-level.
Click on the hyperlinks for more details of the test.

Alert level C

```
PLAT082_ALERT_2_C High R1 Value .................................       0.11 Report
PLAT220_ALERT_2_C Non-Solvent  Resd 1  C   Ueq(max)/Ueq(min) Range       3.3 Ratio
PLAT220_ALERT_2_C Non-Solvent  Resd 1  N   Ueq(max)/Ueq(min) Range       3.5 Ratio
PLAT241_ALERT_2_C High   'MainMol' Ueq as Compared to Neighbors of       C23 Check
PLAT242_ALERT_2_C Low    'MainMol' Ueq as Compared to Neighbors of        N5 Check
PLAT340_ALERT_3_C Low Bond Precision on  C-C Bonds ...............   0.00611 Ang.
PLAT362_ALERT_2_C Short  C(sp3)-C(sp2) Bond  C1       - C5       .      1.40 Ang.
PLAT362_ALERT_2_C Short  C(sp3)-C(sp2) Bond  C4       - C5       .      1.39 Ang.
PLAT413_ALERT_2_C Short Inter XH3 .. XHn     H12C     ..H12C     .      2.11 Ang.
                                                2-x,3/2-y,z  =     6_664 Check
PLAT430_ALERT_2_C Short Inter D...A Contact  O1       ..O1       .      2.90 Ang.
                                                 3-x,1-y,-z  =     9_865 Check
PLAT906_ALERT_3_C Large K Value in the Analysis of Variance ......    17.217 Check
PLAT906_ALERT_3_C Large K Value in the Analysis of Variance ......     3.936 Check
PLAT906_ALERT_3_C Large K Value in the Analysis of Variance ......     2.117 Check
PLAT911_ALERT_3_C Missing FCF Refl Between Thmin & STh/L=    0.600         4 Report
PLAT918_ALERT_3_C Reflection(s) with I(obs) much Smaller I(calc) .         2 Check
PLAT977_ALERT_2_C Check Negative Difference Density on H5B             -0.39 eA-3
```

Alert level G

```
PLAT083_ALERT_2_G SHELXL Second Parameter in WGHT  Unusually Large     25.42 Why ?
PLAT605_ALERT_4_G Largest Solvent Accessible VOID in the Structure       296 A**3
PLAT868_ALERT_4_G ALERTS Due to the Use of _smtbx_masks Suppressed         ! Info
PLAT909_ALERT_3_G Percentage of I>2sig(I) Data at Theta(Max) Still       91% Note
PLAT910_ALERT_3_G Missing # of FCF Reflection(s) Below Theta(Min).         1 Note
PLAT933_ALERT_2_G Number of OMIT Records in Embedded .res File ...         2 Note
PLAT961_ALERT_5_G Dataset Contains no Negative Intensities .......    Please Check
PLAT978_ALERT_2_G Number C-C Bonds with Positive Residual Density.         2 Info
```

```
   0 ALERT level A = Most likely a serious problem - resolve or explain
   0 ALERT level B = A potentially serious problem, consider carefully
  16 ALERT level C = Check. Ensure it is not caused by an omission or oversight
   8 ALERT level G = General information/check it is not something unexpected

   0 ALERT type 1 CIF construction/syntax error, inconsistent or missing data
  13 ALERT type 2 Indicator that the structure model may be wrong or deficient
   8 ALERT type 3 Indicator that the structure quality may be low
   2 ALERT type 4 Improvement, methodology, query or suggestion
   1 ALERT type 5 Informative message, check
```

It is advisable to attempt to resolve as many as possible of the alerts in all categories. Often the minor alerts point to easily fixed oversights, errors and omissions in your CIF or refinement strategy, so attention to these fine details can be worthwhile. In order to resolve some of the more serious problems it may be necessary to carry out additional measurements or structure refinements. However, the purpose of your study may justify the reported deviations and the more serious of these should normally be commented upon in the discussion or experimental section of a paper or in the "special_details" fields of the CIF. checkCIF was carefully designed to identify outliers and unusual parameters, but every test has its limitations and alerts that are not important in a particular case may appear. Conversely, the absence of alerts does not guarantee there are no aspects of the results needing attention. It is up to the individual to critically assess their own results and, if necessary, seek expert advice.

Publication of your CIF in IUCr journals

A basic structural check has been run on your CIF. These basic checks will be run on all CIFs submitted for publication in IUCr journals (*Acta Crystallographica*, *Journal of Applied Crystallography*, *Journal of Synchrotron Radiation*); however, if you intend to submit to *Acta Crystallographica Section C* or *E* or *IUCrData*, you should make sure that full publication checks are run on the final version of your CIF prior to submission.

Publication of your CIF in other journals

Please refer to the *Notes for Authors* of the relevant journal for any special instructions relating to CIF submission.

PLATON version of 03/05/2019; check.def file version of 29/04/2019

Datablock sa670_sq - ellipsoid plot

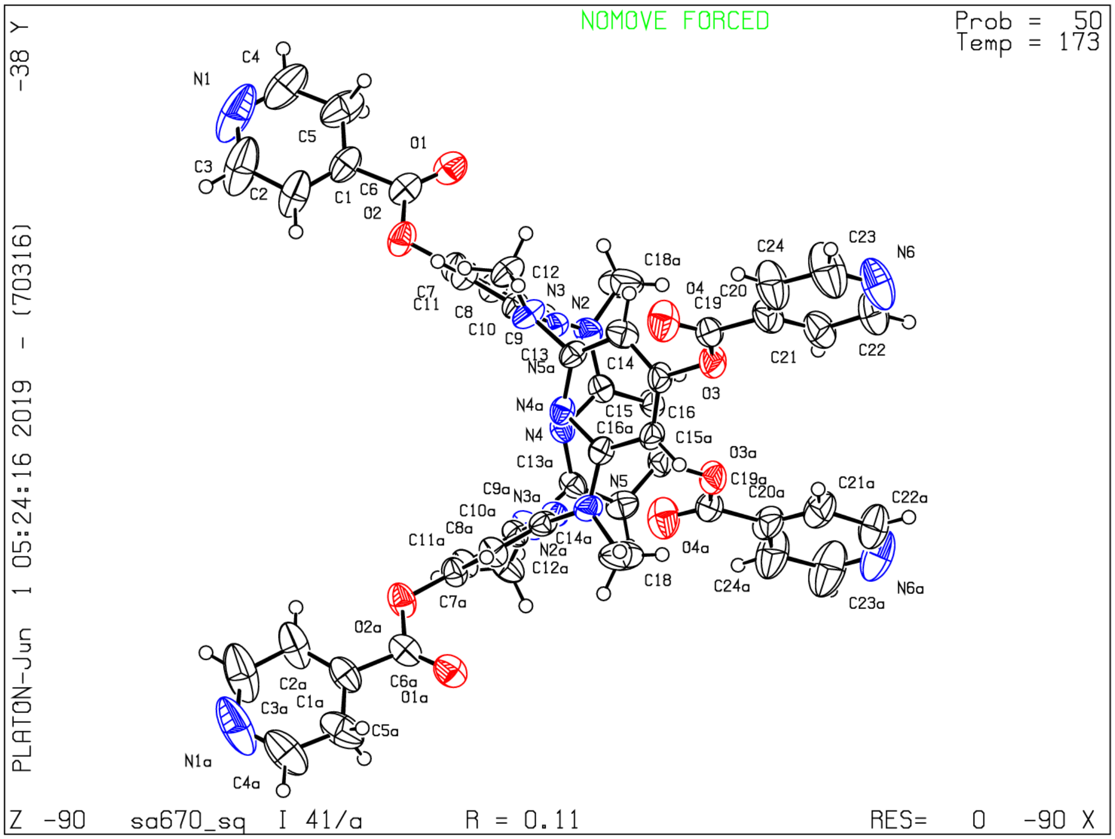

Supplement: Supplementary file 5 [file Data_Sheet_5.PDF]
